# Supplementary material for: Metagenomics of Parkinson’s disease implicates the gut microbiome in multiple disease mechanisms
Source: Nat Commun. 2022 Nov 15;13:6958. doi: 10.1038/s41467-022-34667-x (PMC9663292; doi:10.1038/s41467-022-34667-x)
Supplement: Supplementary file 3 — Description of Additional Supplementary Files [file 41467_2022_34667_MOESM3_ESM.pdf]

Description of Additional Supplementary Files

Metagenomics of Parkinson's disease implicates the gut microbiome in multiple disease mechanisms

Zachary D Wallen, Ayse Demirkan, Guy Twa, Gwendolyn Cohen, Marissa N Dean, David G Standaert, Timothy R Sampson, Haydeh Payami

File name: Supplementary Data
Description:

Supplementary Data 1. Full results of species-level metagenome wide association study (MWAS). Differential abundances of species in PD (N = 490) vs. NHC (N = 234) were tested using two methods, MaAsLin2 and ANCOM-BC, while adjusting for technical covariates (stool sample collection method and total sequence count per sample). Shown are all 719 species and the 'UNKNOWN' group that were detected by MetaPhlAn, however, only species that were present in at least 5% of samples (minimum analytic N=37 samples) were included in MWAS. Species that were detected by MetaPhlAn, but were not tested (NT), are also listed with their sample counts and mean relative abundances in PD and NHC samples. N PD and N NHC: number of PD and neurologically healthy control samples in which the species was detected; RA: mean relative abundance of species from MetaPhlAn, used as input to MaAsLin2; BC-OA: mean sampling bias-corrected observed abundance of species from ANCOM-BC; Beta: coefficient reported by MaAsLin2 or ANCOM-BC; SE: standard error of Beta; P: the uncorrected, two-sided P-value reported by MaAsLin2 or ANCOM-BC; FDR: false discovery rate, i.e., multiple-testing corrected significance q-value, calculated using the Benjamini-Hochberg method; FC: fold change in PD compared to NHC of the relative abundance (MaAsLin2) or of observed abundance (ANCOM-BC) of a species, calculated by taking exponent of Beta using base 2 (MaAsLin2) or natural base (ANCOM-BC); FC lower and upper: lower and upper bound of the 95% confidence interval for the FC; NT: not tested due to prevalence filter of 5% of subjects.

Supplementary Data 2. Full results of genus-level metagenome wide association study (MWAS). Differential abundances of genera in PD (N = 490) vs. NHC (N = 234) were tested using two methods, MaAsLin2 and ANCOM-BC, while adjusting for technical covariates (stool sample collection method and total sequence count per sample). Shown are all 224 genera and the 'UNKNOWN' group that were detected by MetaPhlAn, however, only genera that were present in at least 5% of samples (minimum analytic N=37 samples) were included in MWAS. Genera that were detected by MetaPhlAn, but were not tested (NT), are also listed with their sample counts and mean relative abundances in PD and NHC samples. N PD and N NHC: number of PD and neurologically healthy control samples in which the genus was detected; RA: mean relative abundance of genus from MetaPhlAn, used as input to MaAsLin2; BC-OA: mean sampling bias-corrected observed abundance of genus from ANCOM-BC; Beta: coefficient reported by MaAsLin2 or ANCOM-BC; SE: standard error of Beta; P: the uncorrected, two-sided P-value reported by MaAsLin2 or ANCOM-BC; FDR: false discovery rate, i.e., multiple-testing corrected significance q-value, calculated using the Benjamini-Hochberg method; FC: fold change in PD compared to NHC of the relative abundance (MaAsLin2), or of observed abundance (ANCOM-BC) of a genus, calculated by taking exponent of Beta using base 2 (MaAsLin2) or natural base (ANCOM-BC); FC lower and upper: lower and upper bound of the 95% confidence interval for the FC; NT: not tested due to prevalence filter of 5% of subjects.

Supplementary Data 3. Species-level MWAS adjusted for age and sex.
Analysis included 490 PD and 234 NHC. 84 species identified in MWAS were retested for association with PD using MaAsLin2 with age, sex, total sequence count, and stool collection method in the model. The results show the evidence for association with PD (case status) remains significant for all 84 species at 4E-8<FDR<0.1. Total number of subjects with complete data on all 5 variables was 490 PD and 234 NHC. Beta: coefficient reported by MaAsLin2; SE: standard error of Beta; P: the uncorrected, two-sided P-value reported by MaAsLin2; FDR: false discovery rate, i.e., multiple-testing corrected significance q-value, calculated using the Benjamini-Hochberg method; FC: fold change in relative abundance between groups for each variable calculated by taking the exponent of the Beta with base 2; FC lower and upper: lower and upper bound of the 95% confidence interval for the FC.

Supplementary Data 4. Analysis of confounders.
Analysis included subjects with complete data on all 9 variables, N=435 PD and N=219 NHC. Nine variables, including PD, total sequence count per sample, and 7 potential confounders, were included in a single model and tested simultaneously for association with relative abundance of the 84 species identified in MWAS. MaAsLin2 was used for testing. The variables included were case status (PD vs NHC), total sequence count per sample (continuous variable, standardized using scale function in R), and 7 extrinsic factors whose frequencies differed in PD vs NHC (Table 1), namely: alcohol consumption (yes vs no), use of antihistamines (yes vs no), taking medication for depression/anxiety/mood (yes vs no), taking pain medication (yes vs no), use of laxatives (yes vs no), taking probiotics (yes vs no), taking a sleep aid (yes vs no). Stool collection method was not included as a covariate because the five samples that used swabs did not have data for all of the needed metadata and were excluded. All samples included in confounding analysis were collected uniformly with the OMNIgene GUT kit. The results show the evidence of association of each variable with relative abundance of the species adjusted for the other 8 variables. For sample sizes of each variable see Table 1. Beta: coefficient reported by MaAsLin2; SE: standard error of Beta; P: the uncorrected, two-sided P-value reported by MaAsLin2; FDR: false discovery rate, i.e., multiple-testing corrected significance q-value, calculated using the Benjamini-Hochberg method; FC: fold change in relative abundance between groups for each variable calculated by taking the exponent of the Beta with base 2; FC lower and upper: lower and upper bound of the 95% confidence interval for the FC.

Supplementary Data 5. Pairwise correlation in abundances of species in PD metagenomes.
Analysis included 490 PD cases. All 697 species that were detected by MetaPhlAn in PD samples were tested for pairwise correlations using SparCC. Correlations that were statistically significant (SparCC permuted $P < 0.05$) are shown with the absolute correlation value |r|, whether the correlation was positive (increase together or decrease together) or negative (one going up, the other down), the uncorrected permuted P-value of the correlation, and whether either of the species in the pair was significantly associated with PD in MWAS. Permuted P-values were based on 1,000 permutations, therefore, the lowest P-value achievable was 0.001.

Supplementary Data 6. Pairwise correlation in abundances of species in NHC metagenomes.
Analysis included 234 NHC. All 499 species that were detected by MetaPhlAn in neurologically healthy control samples were tested for pairwise correlations using SparCC. Correlations that were statistically significant (SparCC permuted $P < 0.05$) are shown with the absolute correlation value |r|, whether the correlation was positive (increase together or decrease together) or negative (one going up, the other down), the uncorrected permuted P-value of the correlation, and whether either of the species in the pair was significantly associated with PD in MWAS. Permuted P-values were based on 1,000 permutations, therefore, the lowest P-value achievable was 0.001.

Supplementary Data 7. Clusters of species within the PD and NHC networks.
Analysis included 490 PD and 234 NHC cases. Polymicrobial clusters were defined using pairwise correlations between species (Supplementary Data 5 and 6). Here, we have listed all 719 species that were detected by MetaPhlAn and note for each species if it were elevated in PD, reduced in PD, not significantly different in PD vs. NHC, or not tested due to low prevalence. We then show a summary of network analysis results from PD and NHC metagenomes. For each species, we show the number of other species it correlated with and the cluster it mapped to.

Supplementary Data 8. Cluster #17, a polymicrobial cluster of rare opportunistic pathogens, is elevated in PD.
Network analysis revealed a polymicrobial cluster of opportunistic pathogens, which were also detected and found to be significantly elevated in PD at genus level in our previous dataset (PMID: 32566740). At species level, here, only one species in cluster #17 (Porphyromonas asaccharolytica) was prevalent enough to be tested in MWAS, and it was significantly elevated in PD. The remaining 18 species were individually rare and fell below the prevalence cut-off of 5% and were not tested. To test their collective effect, we collapsed all species into one group, "cluster #17", and tested their cumulative relative abundance in 490 PD vs. 234 NHC. We found cluster #17 significantly elevated in PD (P=2E-5), even after removing Porphyromonas asaccharolytica (P=7E-4). We note the number of correlations for each species in this group, and the average, in PD metagenome and NHC metagenome. The species in

cluster #17 were more tightly correlated in PD metagenome than in NHC metagenome, as depicted by the number of correlations and a species average of 5.4 correlations in PD vs. 1 in NHC. A PubMed search was performed with species name as search term, and as seen in prior dataset at genus level, species in cluster #17 are known as opportunistic pathogens in the literature. N PD and N NHC: number of PD and neurologically healthy control samples the species was detected in; RA: mean relative abundance of species; FC: fold change in the relative abundance in PD compared to NHC; FDR: false discovery rate, i.e., multiple-testing corrected significance q-value, calculated using the Benjamini-Hochberg method; N of correlations: number of species that the named species correlated with at $|r| > 0.2$ and permutation $P < 0.05$.

Supplementary Data 9. Full results of differential abundance analysis of KO (gene family) groups.
Differential abundances of KEGG ortholog (KO) groups in PD (N = 490) vs. NHC (N = 234) were tested using two methods, MaAsLin2 and ANCOM-BC, while adjusting for technical covariates (stool sample collection method and total sequence count per sample). Shown are all 8,528 KO groups that were detected by HUMAnN, however, only KO groups that were present in at least 5% of samples (minimum analytic N=37 samples) were included in differential abundance analysis. KO groups that were detected by HUMAnN, but were not tested (NT), are also listed with their sample counts and mean relative abundances in PD and NHC samples. N PD and N NHC: number of PD and neurologically healthy control samples in which the KO group was detected; RA: mean relative abundance of KO group, used as input to MaAsLin2; BC-OA: mean bias-corrected observed abundance of KO group from ANCOM-BC; Beta: coefficient reported by MaAsLin2 or ANCOM-BC; SE: standard error of Beta; P: the uncorrected, two-sided P-value reported by MaAsLin2 or ANCOM-BC; FDR: false discovery rate, i.e., multiple-testing corrected significance q-value, calculated using the Benjamini-Hochberg method; FC: fold change of the relative abundance (MaAsLin2) or bias-corrected observed abundance (ANCOM-BC) of a KO group in PD compared to NHC, calculated by taking exponent of Beta using base 2 (MaAsLin2) or natural base (ANCOM-BC); FC lower and upper: lower and upper bound of the 95% confidence interval for the FC; NT: not tested due to presence in <5% of subjects; * Labeled "uncharacterized" in HUMAnN v 3.0.0 reference file, but may be able to glean more information by searching KO ID in the KEGG Ortholog database (https://www.genome.jp/kegg/ko.html).

Supplementary Data 10. Full results of differential abundance analysis of pathways.
Differential abundance of MetaCyc pathways was tested in PD (N = 490) vs. NHC (N = 234) using MaAsLin2 and ANCOM-BC, adjusted for technical covariates (stool sample collection method and total sequence count per sample). Shown are all 511 pathways that were detected by HUMAnN, however, only pathways that were present in at least 5% of samples (minimum analytic N=37 samples) were included in differential abundance analysis. Pathways that were detected by HUMAnN, but were not tested (NT), are also listed with their sample counts and mean relative abundances in PD and NHC samples. N PD and N NHC: number of PD and neurologically healthy control samples in which the pathway was detected; RA: mean relative abundance of pathway, used as input to MaAsLin2; BC-OA: mean bias-corrected observed abundance of pathway from ANCOM-BC; Beta: coefficient reported by MaAsLin2 or ANCOM-BC; SE: standard error of Beta; P: the uncorrected, two-sided P-value reported by MaAsLin2 or ANCOM-BC; FDR: false discovery rate, i.e., multiple-testing corrected significance q-value, calculated using the Benjamini-Hochberg method; FC: fold change of the relative abundance (MaAsLin2) or bias-corrected observed abundance (ANCOM-BC) of a pathway in PD compared to NHC, calculated by taking exponent of Beta using base 2 (MaAsLin2) or natural base (ANCOM-BC); FC lower and upper: lower and upper bound of the 95% confidence interval for the FC; NT: not tested due to presence in <5% of subjects; *Pathway name not present in HUMAnN reference file, found by searching MetaCyc ID on the MetaCyc website (metacyc.org).

Supplementary Data 11. Publicly available software and reference databases used in study.

File name: Supplementary Code
Description: Zipped folder that contains an R markdown file and it’s rendered pdf file that documents the workflow and code used to perform bioinformatic processing of shotgun sequences and statistical analyses of microbial profiles and subject metadata.
