# Supplementary material for: Metagenomics of Parkinson’s disease implicates the gut microbiome in multiple disease mechanisms
Source: Nat Commun. 2022 Nov 15;13:6958. doi: 10.1038/s41467-022-34667-x (PMC9663292; doi:10.1038/s41467-022-34667-x)
Supplement: Supplementary file 5 — Supplementary Code [file 41467_2022_34667_MOESM5_ESM.zip › Supplementary_Code.pdf]

Metagenomics of Parkinson's disease implicates the gut microbiome in multiple disease mechanisms

Zachary D Wallen Ayse Demirkan Guy Twa Gwendolyn Cohen
Marissa N Dean David G Standaert Timothy Sampson Haydeh Payami

Contents

Introduction **2**

Bioinformatic processing of sequences **2**
Adapter trimming and quality trimming/filtering of sequences using BBDuk 2
Removal of human host sequence reads using BBSplit/BBMap 3
Removal of low complexity sequences using BBDuk 3
Post quality control exclusions 4
Taxonomic and functional profiling using MetaPhlAn3 and HUMAnN3 4

Setting up R environment for statistical analyses **6**
Create needed functions for analyses 6
Load required R packages 12
Create excel styles used for formatting output 13
Make directories for output 13

Subject characteristics: testing PD vs NHC **13**

Analyses of species and genera **55**
Preparing relative abundance and count data for downstream analyses 55
Principal Component Analysis 57
PERMANOVA and PERMDISP 58
Enterotype analysis: PD vs NHC 60
Differential abundance of species and genera 64
MWAS 64
MaAsLin2 and ANCOM-BC MWAS concordance 71
Genus heterogeneity 73
Species distributions and fold changes from MWAS 75
Sex, age, and confounder analysis 79
Correlation networks 83
SparCC 83
Community detection and creating node and edge files for network visualization 86

Analyses of gene families and pathways **89**
Preparing count data for downstream analyses 89
Differential abundance of gene families and pathways 89
MWAS 89
Sporulation KO group association with constipation 94
Gene family/pathway boxplots with fold changes from MWAS 97

Secondary analyses (replication driven) **116**
Replicating signal for SILVA v 132 Prevotella 116
Replicating signal for opportunitstic pathogen cluster 117

R session information **119**

Introduction

This file documents the workflow used for bioinformatics and code used to perform the statistical analyses detailed in the manuscript ***Metagenomics of Parkinson's disease implicates the gut microbiome in multiple disease mechanisms***. As a brief background, fecal samples from 492 PD and 242 control subjects were sent for shotgun metagenomic sequencing. Shotgun metagenomic sequences were acquired for all samples, quality controlled, decontaminated for human sequences, and filtered for low complexity sequences. Of these samples, 724 (490 PD and 234 controls) were taxonomically profiled using MetaPhlAn3, functionally profiled for gene families and pathways using HUMAnN3, and included in statistical analyses where subject meta-data is taken into account. Taxonomic profiling resulted in species relative abundances (i.e., the % each species makes up out of all species that were detected in a sample) and species counts (i.e., estimation of how many times a species was observed in a sample; calculated by multiplying the relative abundances by the total sequence count for each sample), and functional profiling resulted in gene family and pathway counts, which were used in the statistical analyses detailed below. Raw shotgun metagenomic sequences were uploaded to NCBI SRA where they were decontaminated for human sequences by SRA. Sequences can be accessed under BioProject PRJNA834801.

Bioinformatic processing of sequences

Shotgun sequences were bioinformatically processed from raw sequences to taxonomic and functional (gene families and pathway) profiles using a pipeline that involved sequence QC, decontamination, and low complexity sequence filtering with BBDuk and BBSplit, and taxonomic/functional profiling with MetaPhlAn3 and HUMAnN3. Below describes the steps of the pipeline in brief, along with the shell code used to perform each step.

Adapter trimming and quality trimming/filtering of sequences using BBDuk

Adapter (and PhiX) sequences were removed and quality trimming/filtering of sequence reads was performed using BBDuk with the following parameters:

- `ref=adapters,phix`: For removing common sequencing adapters and PhiX sequences.
- `ftm=5`: For trimming sequences to a length that is multiple of 5 (helps in removing extra base if one exists (i.e. if length of reads is 151 bp instead of 150 bp)).
- `tbo`: In addition to usual kmer adapter trimming, specifies to also trim adapters based on pair overlap detection using BBMerge.
- `tpe`: Specifies to make sure and trim both reads to the same length.
- `qtrim=rl`: Quality trim both 5' and 3' end of sequences.
- `trimq=25` : Quality score to trim up to on sequence ends.
- `minlen=50`: Filter out sequences that fall below 50 bp in length.

The script used to carry out the task above in the HPC environment is shown:

```
# perform adapter trimming and quality trimming/filtering for each sample
bbduk.sh \
in=${FILE_NAME}_R1_001.fastq.gz \
in2=${FILE_NAME}_R2_001.fastq.gz \
```

```
out=Quality_Controlled_Sequences/${FILE_NAME}_R1_001.fastq.gz \
out2=Quality_Controlled_Sequences/${FILE_NAME}_R2_001.fastq.gz \
stats=Quality_Controlled_Sequences/${FILE_NAME}_stats.txt \
ftm=5 tpe tbo qtrim=rl trimq=25 minlen=50 ref=adapters,phix \
-Xmx${MAX_MEM}g
```

Removal of human host sequence reads using BBSplit/BBMap

Human sequence reads were removed from each sequence file by aligning reads to the most recent human genome reference using BBSplit/BBMap.

- The most recent human genome reference (`GCA_000001405.28_GRCh38.p13_genomic.fna`) was downloaded from the NCBI FTP site.
- BBSplit was ran with default parameters for both indexing the human genome reference file and for mapping sequences.

The script used to carry out the task above in the HPC environment is shown:

```
# index reference genome file
bbsplit.sh \
ref=GCA_000001405.28_GRCh38.p13_genomic.fna \
path=Decontaminated_Sequences \
t=$CPU_REQUEST \
-Xmx${MAX_MEM}g

# perform decontamination for each sample
bbsplit.sh \
in1=Quality_Controlled_Sequences/${FILE_NAME}_R1_001.fastq.gz \
in2=Quality_Controlled_Sequences/${FILE_NAME}_R2_001.fastq.gz \
outu1=Decontaminated_Sequences/${FILE_NAME}_R1_001.fastq.gz \
outu2=Decontaminated_Sequences/${FILE_NAME}_R2_001.fastq.gz \
path=Decontaminated_Sequences \
basename=Decontaminated_Sequences/${FILE_NAME}.%_contam_#.fastq.gz \
refstats=Decontaminated_Sequences/${FILE_NAME}_refstats.log \
t=$CPU_REQUEST \
-Xmx${MAX_MEM}g
```

Removal of low complexity sequences using BBDuk

To remove low complexity sequences such as mononucleotide repeats, BBDuk entropy filtering was ran with following parameters:

- `entropy=0.01`: Entropy threshold for removing low complexity sequences. This value suggested by BBDuk author to remove only monomeric repeats (http://seqanswers.com/forums/showthread.php?t=42776&page=7).
- `entropywindow=50`: Calculate entropy using a sliding window of this length. Value is the default.
- `entropyk=5`: Calculate entropy using kmers of this length. Value is the default.

The script used to carry out the task above in the HPC environment is shown:

```
# concatenate paired sequence files for each sample
zcat Decontaminated_Sequences/${FILE_NAME}_R1_001.fastq.gz \
Decontaminated_Sequences/${FILE_NAME}_R2_001.fastq.gz \
> ${FILE_NAME}.fastq

```

```
# perform entropy filtering on concatenated paired sequence files
bbduk.sh \
in=${FILE_NAME}.fastq \
out=Low_Complexity_Filtered_Sequences/${FILE_NAME}.fastq.gz \
outm=Low_Complexity_Filtered_Sequences/Removed_Sequences/${FILE_NAME}.fastq.gz \
entropy=0.01 \
entropywindow=50 \
entropyk=5 \
-Xmx${MAX_MEM}g
```

Post quality control exclusions

- Duplicate PD sample, N=1 (not uploaded to SRA)
- NHC samples whose subjects reported to have a neurological condition, N=8 (not uploaded to SRA)
- PD sample with high human DNA contamination and low sequence count (< 10M) after QC, N=1.
  - Note: sequences for this sample were made available on SRA repository
- This made the final set of quality controlled sequences range from 12M - 285M sequences per sample for 490 PD and 234 NHC.

Taxonomic and functional profiling using MetaPhlAn3 and HUMAnN3

Quality controlled, decontaminated, and low complexity filtered sequences were profiled for taxonomic and functional content using `MetaPhlAn v 3.0.14` that performs marker gene based taxonomic profiling to get relative abundances of microbial clades present in each sample, then using `HUMAnN v 3.0.0` to determine the microbial gene family and metabolic pathway content present in each sample using the UniRef and MetaCyc databases.

- Taxonomic profiling with MetaPhlAn was performed once with default parameters, and then a second time adding the `--unknown_estimation` flag. This flag was enabled so the relative abundances of clades would take the unknown content of a samples metagenome (portion of sequenced metagenome that is not contained in the MetaPhlAn database) into account (important for calculating count data). When the second run of MetaPhlAn was complete, estimated counts for each clade were derived by multiplying the relative abundances with unknown estimation by the total read count reported by the bowtie2 intermediate file from running MetaPhlAn (formula: `(relative abundance / 100) x nread from bowtie2`).

The script used to carry out the task above in the HPC environment is shown:

```
# perform taxonomic profiling with default parameters for each sample
metaphlan \
Low_Complexity_Filtered_Sequences/${FILE_NAME}.fastq.gz \
--input_type fastq \
-t rel_ab \
--nproc $CPU_REQUEST \
--bowtie2out Taxonomic_Profiling/${FILE_NAME}_metaphlan_bowtie2.txt \
-o Taxonomic_Profiling/${FILE_NAME}_metaphlan_rel_ab.tsv

# perform taxonomic profiling adding unknown estimation for each sample
metaphlan \
Low_Complexity_Filtered_Sequences/${FILE_NAME}.fastq.gz \
--input_type fastq \
-t rel_ab \
--unknown_estimation \
--nproc $CPU_REQUEST \
```

```
--bowtie2out Taxonomic_Profiling/${FILE_NAME}_metaphlan_bowtie2.txt \
-o Taxonomic_Profiling/${FILE_NAME}_metaphlan_rel_ab_w_unknown.tsv

# calculate count data using relative abundances with unknown estimation
NREADS=$(grep '#nreads' Taxonomic_Profiling/${FILE_NAME}_metaphlan_bowtie2.txt | \
         awk '{print $2}')

grep -v '^#' Taxonomic_Profiling/${FILE_NAME}_metaphlan_rel_ab_w_unknown.tsv | \
awk -v nreads="$NREADS" '{OFMT="%f";print $1,$2,$3,($3/100)*nreads,$4}' OFS='\t' | \
cat <(grep '^#' Taxonomic_Profiling/${FILE_NAME}_metaphlan_rel_ab_w_unknown.tsv) - | \
sed 's/#clade_name\tNCBI_tax_id\trelative_abundance\tadditional_species/
#clade_name\tNCBI_tax_id\trelative_abundance\tcounts\tadditional_species/' \
> Taxonomic_Profiling/${FILE_NAME}_metaphlan_rel_ab_w_counts.tsv

# extract count data
awk -F'\t' '{print $1,$2,$4}' OFS='\t' \
Taxonomic_Profiling/${FILE_NAME}_metaphlan_rel_ab_w_counts.tsv \
> Taxonomic_Profiling/${FILE_NAME}_metaphlan_counts.tsv

# merge individual sample files
merge_metaphlan_tables.py \
Taxonomic_Profiling/*_metaphlan_rel_ab.tsv > Taxonomic_Profiling/metaphlan_rel_ab.tsv
sed -i '2s/_metaphlan_rel_ab//g' Taxonomic_Profiling/metaphlan_rel_ab.tsv

merge_metaphlan_tables.py \
Taxonomic_Profiling/*_metaphlan_counts.tsv > Taxonomic_Profiling/metaphlan_counts.tsv
sed -i '2s/_metaphlan_counts//g' Taxonomic_Profiling/metaphlan_counts.tsv
```

- Functional profiling with HUMAnN was performed with default parameters using the ChocoPhlAn database `full_chocophlan.v296_201901b` and UniRef database `uniref90_annotated_v201901b_full` with sequences grouped at 90% identity.

```
# perform functional profiling with default parameters for each sample
humann \
--input Low_Complexity_Filtered_Sequences/${FILE_NAME}.fastq.gz \
--output Functional_Profiling \
--output-basename $FILE_NAME \
--nucleotide-database full_chocophlan.v296_201901b \
--protein-database uniref90_annotated_v201901b_full \
--metaphlan-options '-t rel_ab' \
--prescreen-threshold 0.01 \
--threads $CPU_REQUEST \
--verbose

# merge per sample tables by code provided by developers
humann_join_tables \
--input Functional_Profiling/ \
--output Functional_Profiling/humann_genefamilies.tsv \
--file_name genefamilies.tsv

humann_join_tables \
--input Functional_Profiling/ \
--output Functional_Profiling/humann_pathabundance.tsv \
--file_name pathabundance.tsv
```

- To reduce the number of gene families being analyzed, default HUMAnN gene families (UniRef90) were converted to KEGG ortholog (KO) groups using the `humann_regroup_table` script packaged with HUMAnN3 specifying the mapping file to be `map_ko_uniref90.txt.gz`. This was performed on gene family tables for each sample.
  - Note: on average per sample, 4-7% of gene families were able to be regrouped into KO groups
  - Per sample KO group abundances were then merged to create one file for all samples

```
# convert UniRef90 gene families to KO groups for each sample
humann_regroup_table \
-i Functional_Profiling/${FILE_NAME}_genefamilies.tsv \
-c full_mapping_v201901b/map_ko_uniref90.txt.gz \
-o Functional_Profiling/${FILE_NAME}_humann_KO_group_counts.tsv

# merge per sample tables
humann_join_tables \
--input Functional_Profiling/ \
--output Functional_Profiling/humann_KO_group_counts.tsv \
--file_name humann_KO_group_counts.tsv
```

- Pathway and KO group abundance tables were outputted in stratified format (contains abundances broken up by contributing species), therefore, pathway and KO group abundance files were subsetted for only the community level abundances using the following commands:

```
# extract only community level abundances
grep -v '|' Functional_Profiling/humann_pathabundance.tsv \
> Functional_Profiling/humann_pathabundance.tsv
grep -v '|' Functional_Profiling/humann_KO_group_counts.tsv \
> Functional_Profiling/humann_KO_group_counts.tsv
```

- Then, more informative names were given to KO groups using the `humann_rename_table` script packaged with HUMAnN3 specifying the naming file to be `map_ko_name.txt.gz`.

```
# add KO group names to file
humann_rename_table \
-i Functional_Profiling/humann_KO_group_counts.tsv \
-c full_mapping_v201901b/map_ko_name.txt.gz \
-o Functional_Profiling/humann_KO_group_counts.tsv
```

- The above taxonomic and functional profiling resulted in 4 tables: microbial clade relative abundances and estimated counts, KO group RPK abundances, and pathway RPK abundances. These tables, along with subject metadata, were used as input for the statistical analyses and generating figures, and can be found in the Source Data file provided with the manuscript.

Setting up R environment for statistical analyses

Create needed functions for analyses

```
#### CREATE FUNCTIONS NEEDED FOR RUNNING CODE ####

# Function to supress warnings and messages
# parameters:
#     x - the command to supress messages/warnings of
suppress <- function(x){invisible(capture.output(suppressMessages(suppressWarnings(x))))}

# Function to perform ANCOM-BC and perform additional actions like grabbing summary stats
```

```r
# and calculating bias-corrected abundances. Note: this function should work with older
# versions of ANCOM-BC that used the 'zero_cut' parameter or newer versions that use the
# 'prv_cut' parameter.
# parameters:
#             ps - phyloseq object with otu_table() and sample_data() data
#        formula - formula for model to be tested by ANCOM-BC. make sure categorical
#                  variables have been dummy coded with 1 (for test group) and
#                  0 (reference group) or else the N calculations will not work
#                  correctly.
#   remaining parameters have been set to ANCOM-BC defaults (see ANCOM-BC documentation)
ANCOMBC.plus <- function(ps, formula,
                         p_adj_method="holm",
                         zero_cut=0.9,
                         lib_cut=0,
                         group=NULL,
                         struc_zero=FALSE,
                         neg_lb=FALSE,
                         tol=1E-5,
                         max_iter=100,
                         conserve=FALSE,
                         alpha=0.05,
                         global=FALSE){
  library(phyloseq)
  library(ANCOMBC)
  ci <- function(coef, se){
    lower.ci <- coef - 1.96*se
    upper.ci <- coef + 1.96*se
    return(c(lower.ci=lower.ci,upper.ci=upper.ci))
  }
  # make sure samples are rows and features are columns
  if (taxa_are_rows(ps)){
    ps <- phyloseq(t(otu_table(ps)), sample_data(ps))
  }
  # run ANCOM-BC
  if ('prv_cut' %in% names(as.list(args(ancombc)))){
    suppressWarnings(
    ancom.res <- ancombc(ps, formula, p_adj_method, 1-zero_cut, lib_cut,
                         group, struc_zero, neg_lb, tol, max_iter,
                         conserve, alpha, global)
    )
  }else{
    suppressWarnings(
    ancom.res <- ancombc(ps, formula, p_adj_method, zero_cut, lib_cut,
                         group, struc_zero, neg_lb, tol, max_iter,
                         conserve, alpha, global)
    )
  }

  # calculate bias-corrected abundances
  samp_frac <- ancom.res$samp_frac
  samp_frac[is.na(samp_frac)] <- 0
  ps.adj <- prune_taxa(rownames(ancom.res$feature_table), ps)
  otu_table(ps.adj) <- log(otu_table(ps.adj) + 1) - samp_frac
  # filter for samples with data for variables included in model
```

```
for (var in seq_len(ncol(ancom.res$res[[1]]))){
  ps <- phyloseq(otu_table(ps),
    sample_data(ps)[!is.na(sample_data(ps)[,strsplit(formula,
                                                     " \\+ ")[[1]][var]]),])
  ps.adj <- phyloseq(otu_table(ps.adj),
    sample_data(ps.adj)[!is.na(sample_data(ps.adj)[,strsplit(formula,
                                                             " \\+ ")[[1]][var]]),])
}
# get summary of results
res <- data.frame()
for (var in seq_len(ncol(ancom.res$res[[1]]))){
  # get variable name
  var.name1 <- strsplit(formula, " \\+ ")[[1]][var]
  var.name2 <- colnames(ancom.res$res[[1]])[var]
  # get N in each group
  if (length(table(sample_data(ps)[,var.name1])) == 2){
    group.1.index <- sample_data(ps)[,var.name1] ==
                     names(table(sample_data(ps)[,var.name1]))[2]
    group.1.index[is.na(group.1.index)] <- FALSE
    group.2.index <- sample_data(ps)[,var.name1] ==
                     names(table(sample_data(ps)[,var.name1]))[1]
    group.2.index[is.na(group.2.index)] <- FALSE
    n1 <- colSums(otu_table(ps)[group.1.index,] > 0)
    n2 <- colSums(otu_table(ps)[group.2.index,] > 0)
    mean1 <- exp(colMeans(otu_table(ps.adj)[group.1.index,]))
    mean2 <- exp(colMeans(otu_table(ps.adj)[group.2.index,]))
  }else{
    n1 <- rep(nsamples(ps), ntaxa(ps))
    names(n1) <- taxa_names(ps)
    n2 <- rep(NA, ntaxa(ps))
    names(n2) <- taxa_names(ps)
    mean1 <- exp(colMeans(otu_table(ps.adj)))
    mean2 <- rep(NA, ntaxa(ps.adj))
    names(mean2) <- taxa_names(ps.adj)
  }
  # calculate fold change and confidence interval of fold change
  if(length(table(sample_data(ps)[,var.name1])) == 2){
    FC <- exp(ancom.res$res[[1]][,var.name2])
    FC.lower <- c()
    FC.upper <- c()
    for (coef in seq_along(ancom.res$res[[1]][,var.name2])){
      FC.lower <- c(FC.lower, exp(ci(ancom.res$res[[1]][,var.name2][coef],
                                     ancom.res$res[[2]][,var.name2][coef])['lower.ci']))
      FC.upper <- c(FC.upper, exp(ci(ancom.res$res[[1]][,var.name2][coef],
                                     ancom.res$res[[2]][,var.name2][coef])['upper.ci']))
    }
  }else{
    FC <- NA
    FC.lower <- NA
    FC.upper <- NA
  }
  # summarize results for variable
  rvar <- data.frame(Variable=var.name1,
                     Feature=rownames(ancom.res$feature_table),
```

```
                    N1=n1[rownames(ancom.res$feature_table)],
                    N2=n2[rownames(ancom.res$feature_table)],
                    Mean1=mean1[rownames(ancom.res$feature_table)],
                    Mean2=mean2[rownames(ancom.res$feature_table)],
                    Beta=ancom.res$res[[1]][,var.name2],
                    SE=ancom.res$res[[2]][,var.name2],
                    P=ancom.res$res[[4]][,var.name2],
                    FDR=ancom.res$res[[5]][,var.name2],
                    FC=FC, FC_lower=FC.lower, FC_upper=FC.upper,
                    check.names=FALSE)
    res <- rbind(res, rvar[order(rvar$P),])
    # add untested features if they exist
    if (nrow(rvar) != ntaxa(ps)){
      res <- rbind(res,
             data.frame(Variable=var.name1,
                        Feature=taxa_names(ps)[!(taxa_names(ps) %in%
                                         rownames(ancom.res$feature_table))],
                        N1=n1[taxa_names(ps)[!(taxa_names(ps) %in%
                                       rownames(ancom.res$feature_table))]],
                        N2=n2[taxa_names(ps)[!(taxa_names(ps) %in%
                                       rownames(ancom.res$feature_table))]],
                        Mean1=NA, Mean2=NA, Beta=NA, SE=NA, P=NA, FDR=NA,
                        FC=NA, FC_lower=NA, FC_upper=NA,
                        check.names=FALSE))
    }
  }
  return(list(result.summary=res, ancom.output=ancom.res, bias.corrected.ps=ps.adj))
}

# Function to perform MaAsLin2 on phyloseq object and
# perform additional actions like grabbing summary stats
# parameters:
#             ps - phyloseq object with otu_table() and sample_data() data
#         output - path to directory for output
#       metadata - vector listing variable names in sample_data() to include in the model
# remaining parameters have been set to Maaslin2 defaults (see Maaslin2 documentation)
MaAsLin2.plus <- function(ps, output, metadata,
                          min_abundance=0,
                          min_prevalence=0.1,
                          min_variance=0,
                          normalization='TSS',
                          transform='LOG',
                          analysis_method='LM',
                          max_significance=0.25,
                          random_effects=NULL,
                          fixed_effects=NULL,
                          correction='BH',
                          standardize=TRUE,
                          cores=1,
                          plot_heatmap=TRUE,
                          plot_scatter=TRUE,
                          heatmap_first_n=50,
                          reference=NULL){
  library(phyloseq)
```

```
library(Maaslin2)
ci <- function(coef, se){
  lower.ci <- coef - 1.96*se
  upper.ci <- coef + 1.96*se
  return(c(lower.ci=lower.ci,upper.ci=upper.ci))
}
# make sure samples are rows and features are columns
if (taxa_are_rows(ps)){
  ps <- phyloseq(t(otu_table(ps)), sample_data(ps))
}
# make sure only LOG is chosen for MaAsLin transformation
if (!(transform == "LOG")){
  stop('function only supports LOG transformation at this time')
}
# run MaAsLin2
input_data <- data.frame(otu_table(ps))
input_metadata <- data.frame(sample_data(ps)[,colnames(sample_data(ps)) %in% metadata])
fits <- Maaslin2(input_data, input_metadata, output, min_abundance, min_prevalence,
                 min_variance,normalization, transform, analysis_method, max_significance,
                 random_effects, fixed_effects, correction, standardize, cores,
                 plot_heatmap, plot_scatter, heatmap_first_n, reference)
# put back original feature names
for (feat in seq_along(fits$results$feature)){
  fits$results$feature[feat] <- taxa_names(ps)[make.names(taxa_names(ps)) ==
                                                  fits$results$feature[feat]]
}
# filter for samples with data for variables included in model
for (var in seq_along(unique(fits$results$metadata))){
  ps <- phyloseq(otu_table(ps),
                 sample_data(ps)[!is.na(sample_data(ps)[,metadata[var]]),])
}
# get summary of results
res <- data.frame()
for (var in seq_along(unique(fits$results$metadata))){
  # get variable name
  var.name <- metadata[var]
  # get N in each group
  if (length(table(sample_data(ps)[,var.name])) == 2){
    group.1.index <- sample_data(ps)[,var.name] ==
                     names(table(sample_data(ps)[,var.name]))[2]
    group.1.index[is.na(group.1.index)] <- FALSE
    group.2.index <- sample_data(ps)[,var.name] ==
                     names(table(sample_data(ps)[,var.name]))[1]
    group.2.index[is.na(group.2.index)] <- FALSE
    n1 <- colSums(otu_table(ps)[group.1.index,] > 0)
    n2 <- colSums(otu_table(ps)[group.2.index,] > 0)
    mean1 <- colMeans(otu_table(ps)[group.1.index,])
    mean2 <- colMeans(otu_table(ps)[group.2.index,])
  }else{
    n1 <- rep(sum(table(sample_data(ps)[,var.name])), ntaxa(ps))
    names(n1) <- taxa_names(ps)
    n2 <- rep(NA, ntaxa(ps))
    names(n2) <- taxa_names(ps)
    mean1 <- colMeans(otu_table(ps))
```

```
      mean2 <- rep(NA, ntaxa(ps))
      names(mean2) <- taxa_names(ps)
    }
    # calculate fold change and confidence interval of fold change
    if(length(table(sample_data(ps)[,var.name])) == 2){
      FC <- 2^(fits$results$coef[fits$results$metadata == var.name])
      FC.lower <- c()
      FC.upper <- c()
      for (coef in seq_along(fits$results$coef[fits$results$metadata == var.name])){
        FC.lower <- c(FC.lower, 2^(ci(fits$results$coef[fits$results$metadata ==
                                                        var.name][coef],
                                     fits$results$stderr[fits$results$metadata ==
                                                          var.name][coef])['lower.ci']))
        FC.upper <- c(FC.upper, 2^(ci(fits$results$coef[fits$results$metadata ==
                                                        var.name][coef],
                                     fits$results$stderr[fits$results$metadata ==
                                                          var.name][coef])['upper.ci']))
      }
    }else{
      FC <- NA
      FC.lower <- NA
      FC.upper <- NA
    }
    # summarize results for variable
    rvar <- data.frame(Variable=var.name,
                       Feature=fits$results$feature[fits$results$metadata == var.name],
                       N1=n1[fits$results$feature[fits$results$metadata == var.name]],
                       N2=n2[fits$results$feature[fits$results$metadata == var.name]],
                       Mean1=mean1[fits$results$feature[fits$results$metadata == var.name]],
                       Mean2=mean2[fits$results$feature[fits$results$metadata == var.name]],
                       Beta=fits$results$coef[fits$results$metadata == var.name],
                       SE=fits$results$stderr[fits$results$metadata == var.name],
                       P=fits$results$pval[fits$results$metadata == var.name],
                       FDR=p.adjust(fits$results$pval[fits$results$metadata == var.name],
                                    method=correction),
                       FC=FC, FC_lower=FC.lower, FC_upper=FC.upper,
                       check.names=FALSE)
    res <- rbind(res, rvar[order(rvar$P),])
    # add untested features if they exist
    if (nrow(rvar) != ntaxa(ps)){
      res <- rbind(res,
             data.frame(Variable=var.name,
                        Feature=taxa_names(ps)[!(taxa_names(ps) %in%
                              fits$results$feature[fits$results$metadata == var.name])],
                        N1=n1[taxa_names(ps)[!(taxa_names(ps) %in%
                              fits$results$feature[fits$results$metadata == var.name])]],
                        N2=n2[taxa_names(ps)[!(taxa_names(ps) %in%
                              fits$results$feature[fits$results$metadata == var.name])]],
                        Mean1=mean1[taxa_names(ps)[!(taxa_names(ps) %in%
                              fits$results$feature[fits$results$metadata == var.name])]],
                        Mean2=mean2[taxa_names(ps)[!(taxa_names(ps) %in%
                              fits$results$feature[fits$results$metadata == var.name])]],
                        Beta=NA, SE=NA, P=NA, FDR=NA, FC=NA, FC_lower=NA, FC_upper=NA,
                        check.names=FALSE)
```

```r
                    )
    }
  }
return(list(result.summary=res, Maaslin2.output=fits))
}

# Function to compute log2 transform for transforming relative abundances
# parameters:
#    x - a vector or data.frame of relative abundances
log2.trans <- function(x) {
  y <- replace(x, x == 0, min(x[x>0]) / 2)
  return(log2(y))
}

# Function to convert numbers from strings back to numbers in excel output
# parameters:
#       df - the data.frame being outputted into excel
#       wb - the excel workbook object
#    sheet - the excel sheet name
# colnames - does df have column names, TRUE/FALSE
convertNum <- function(df, wb, sheet, colnames) {
  library(foreach)
  cn <- expand.grid(seq_len(ncol(df)), seq_len(nrow(df)))[,1]
  rn <- expand.grid(seq_len(ncol(df)), seq_len(nrow(df)))[,2]
  trash <- foreach(cn=cn, rn=rn) %dopar% {
    if (!is.numeric(df[rn,cn]) && !is.na(as.numeric(as.character(df[rn,cn])))) {
      row.offset <- ifelse(colnames, 1, 0)
      openxlsx::writeData(wb, sheet, as.numeric(as.character(df[rn,cn])),
                          startCol=cn, startRow=row.offset + rn)
    }
  }
}
```

Load required R packages

```r
#### LOAD REQUIRED R PACKAGES ####

# standard data manipulation R packages
suppress(library(dplyr))
suppress(library(reshape2))
suppress(library(readxl))
suppress(library(phyloseq))
suppress(library(tibble))
suppress(library(openxlsx))
suppress(library(foreach))
suppress(library(data.table))
suppress(library(gridExtra))
suppress(library(scales))
# R packages used in analysis or plotting
suppress(library(ggplot2))
suppress(library(ggh4x))
suppress(library(ggfortify))
suppress(library(ggvenn))
```

```
suppress(library(ggrepel))
suppress(library(vegan))
suppress(library(pairwiseCI))
suppress(library(vcd))
suppress(library(ANCOMBC))
suppress(library(Maaslin2))
suppress(library(igraph))
```

Create excel styles used for formatting output

```
#### CREATE EXCEL STYLES USED FOR FORMATTING OUTPUT ####

bold <- createStyle(textDecoration="bold")
center <- createStyle(halign="center", valign="center", wrapText=TRUE)
horizontal_border_med <- createStyle(border="top", borderStyle="medium")
horizontal_border_thin <- createStyle(border="top", borderStyle="thin")
```

Make directories for output

```
#### CREATE OUTPUT DIRECTORIES ####

system('mkdir PDShotgunAnalysis_out')
system('mkdir PDShotgunAnalysis_out/1.Metadata')
system('mkdir PDShotgunAnalysis_out/2.Gut_microbiome_composition')
system('mkdir PDShotgunAnalysis_out/3.Taxonomic_associations')
system('mkdir PDShotgunAnalysis_out/4.a.Network_analysis')
system('mkdir PDShotgunAnalysis_out/4.b.Gephi_network_plots')
system('mkdir PDShotgunAnalysis_out/5.Gene_pathway_associations')
system('mkdir PDShotgunAnalysis_out/6.Secondary_analyses')
```

Subject characteristics: testing PD vs NHC

To determine what subject metadata variables are significantly different between PD and NHC subjects, tested each variable for association with PD using Fisher's exact test (via `fisher.test` function) for categorical variables and Wilcoxon rank-sum test (via `wilcox.test` function) for quantitative variables. Odds ratios and confidence intervals were calculated via the `fisher.test` function. If any 2x2 tables of categorical variables contained 0, then the function `Prop.or` from the `pairwiseCI` R package specifying `CImethod='Woolf'` was used to calculate odds ratios and confidence intervals. Subject metadata can be found in the Source Data file included with the manuscript.

```
#### SUBJECT CHARACTERISTICS PD VS NHC ####

# read in metadata
metadata <- data.frame(read_xlsx('Source_Data.xlsx', sheet='subject_metadata'))
rownames(metadata) <- metadata$sample_name

# make new data.frame for metadata, so we do not alter original data.frame
subject.data <- metadata

# create result data.frame
results <- data.frame()
```

```r

# samples passing QC
results <- rbind(results,
                 data.frame(Category='',
                      Metadata="Number of subjects who passed sequence and metadata QC",
                      `PD N`=table(subject.data$Case_status)['PD'],
                      `PD summary stats`="-",
                      `NHC N`=table(subject.data$Case_status)['Control'],
                      `NHC summary stats`="-",
                      `Total N`=length(na.omit(subject.data$Case_status)),
                      P="-", `OR [95%CI]`="-", check.names=FALSE))
# age
P.t <- length(na.omit(subset(subject.data, Case_status == "PD")$Age_at_collection))
P.avg <- mean(na.omit(subset(subject.data, Case_status == "PD")$Age_at_collection))
P.sd <- sd(na.omit(subset(subject.data, Case_status == "PD")$Age_at_collection))
C.t <- length(na.omit(subset(subject.data, Case_status == "Control")$Age_at_collection))
C.avg <- mean(na.omit(subset(subject.data, Case_status == "Control")$Age_at_collection))
C.sd <- sd(na.omit(subset(subject.data, Case_status == "Control")$Age_at_collection))
p <- wilcox.test(subset(subject.data, Case_status == "PD")$Age_at_collection,
                 subset(subject.data, Case_status == "Control")$Age_at_collection)$p.value
results <- rbind(results, data.frame(Category='',
                                     Metadata ="Age",
                                     `PD N`=P.t,
                                     `PD summary stats`=paste(round(P.avg, 1),
                                                              round(P.sd, 1),
                                                              sep="±"),
                                     `NHC N`=C.t,
                                     `NHC summary stats`=paste(round(C.avg, 1),
                                                               round(C.sd, 1),
                                                               sep="±"),
                                     `Total N`=P.t+C.t,
                                     P=formatC(p, format="e", digits=1),
                                     `OR [95%CI]`="-",
                                     check.names=FALSE))
# sex
P.f <- table(subset(subject.data, Case_status == "PD")$Sex)['F']
P.m <- table(subset(subject.data, Case_status == "PD")$Sex)['M']
C.f <- table(subset(subject.data, Case_status == "Control")$Sex)['F']
C.m <- table(subset(subject.data, Case_status == "Control")$Sex)['M']
p <- fisher.test(matrix(c(P.m,P.f,C.m,C.f), nrow=2))$p.value
or <- fisher.test(matrix(c(P.m,P.f,C.m,C.f), nrow=2))$estimate
ci <- paste(round(fisher.test(matrix(c(P.m,P.f,C.m,C.f), nrow=2))$conf.int[1],1),
            round(fisher.test(matrix(c(P.m,P.f,C.m,C.f), nrow=2))$conf.int[2],1), sep='-')
results <- rbind(results, data.frame(Category='',
                                     Metadata ="Sex (N & % male)",
                                     `PD N`=P.f+P.m,
                                     `PD summary stats`=paste(P.m,
                                                              " ","(",
                                                              round(P.m/(P.f+P.m)*100, 0),
                                                              "%",")",
                                                              sep=""),
                                     `NHC N`=C.f+C.m,
                                     `NHC summary stats`=paste(C.m,
                                                               " ","(",
```

```
                                                                      round(C.m/(C.f+C.m)*100, 0),
                                                                      "%",")",
                                                                      sep=""),
                                    `Total N`=P.f+P.m+C.f+C.m,
                                    P=formatC(p, format="e", digits=1),
                                    `OR [95%CI]`=paste(round(or, 1),' [',ci,']',sep=''),
                                    check.names=FALSE))
# hispanic or latino
P.y <- table(subset(subject.data, Case_status == "PD")$Hispanic_or_Latino)['Y']
P.n <- table(subset(subject.data, Case_status == "PD")$Hispanic_or_Latino)['N']
C.y <- table(subset(subject.data, Case_status == "Control")$Hispanic_or_Latino)['Y']
C.n <- table(subset(subject.data, Case_status == "Control")$Hispanic_or_Latino)['N']
if (is.na(P.n)){P.n <- 0}
if (is.na(P.y)){P.y <- 0}
if (is.na(C.n)){C.n <- 0}
if (is.na(C.y)){C.y <- 0}
p <- fisher.test(matrix(c(P.y,P.n,C.y,C.n), nrow=2))$p.value
if (any(c(P.y, P.n, C.y, C.n)==0)){
  or <- Prop.or(x=c(P.y,P.n), y=c(C.y,C.n), CImethod='Woolf')$estimate
  ci <- paste(round(Prop.or(x=c(P.y,P.n), y=c(C.y,C.n),
                            CImethod='Woolf')$conf.int[1],1),
              round(Prop.or(x=c(P.y,P.n), y=c(C.y,C.n),
                            CImethod='Woolf')$conf.int[2],1), sep='-')
}else{
  or <- fisher.test(matrix(c(P.y,P.n,C.y,C.n), nrow=2))$estimate
  ci <- paste(round(fisher.test(matrix(c(P.y,P.n,C.y,C.n),
                                       nrow=2))$conf.int[1],1),
              round(fisher.test(matrix(c(P.y,P.n,C.y,C.n),
                                       nrow=2))$conf.int[2],1), sep='-')
}
results <- rbind(results, data.frame(Category='Ancestry',
                                     Metadata ="Hispanic or Latino",
                                     `PD N`=P.n+P.y,
                                     `PD summary stats`=paste(P.y,
                                                             " ","(",
                                                             round(P.y/(P.n+P.y)*100, 0),
                                                             "%",")",
                                                               sep=""),
                                     `NHC N`=C.n+C.y,
                                     `NHC summary stats`=paste(C.y,
                                                              " ","(",
                                                              round(C.y/(C.n+C.y)*100, 0),
                                                              "%",")",
                                                                sep=""),
                                     `Total N`=P.n+P.y+C.n+C.y,
                                     P=formatC(p, format="e", digits=1),
                                     `OR [95%CI]`=paste(round(or, 1),' [',ci,']',sep=''),
                                     check.names=FALSE))
# race
P.y <- table(subset(subject.data, Case_status == "PD")$Race)['White']
P.n <- sum(table(subset(subject.data, Case_status == "PD")$Race))-P.y
C.y <- table(subset(subject.data, Case_status == "Control")$Race)['White']
C.n <- sum(table(subset(subject.data, Case_status == "Control")$Race))-C.y
if (is.na(P.n)){P.n <- 0}
```

```
if (is.na(P.y)){P.y <- 0}
if (is.na(C.n)){C.n <- 0}
if (is.na(C.y)){C.y <- 0}
p <- fisher.test(matrix(c(P.y,P.n,C.y,C.n), nrow=2))$p.value
if (any(c(P.y, P.n, C.y, C.n)==0)){
  or <- Prop.or(x=c(P.y,P.n), y=c(C.y,C.n), CImethod='Woolf')$estimate
  ci <- paste(round(Prop.or(x=c(P.y,P.n), y=c(C.y,C.n),
                            CImethod='Woolf')$conf.int[1],1),
              round(Prop.or(x=c(P.y,P.n), y=c(C.y,C.n),
                            CImethod='Woolf')$conf.int[2],1), sep='-')
}else{
  or <- fisher.test(matrix(c(P.y,P.n,C.y,C.n), nrow=2))$estimate
  ci <- paste(round(fisher.test(matrix(c(P.y,P.n,C.y,C.n),
                                       nrow=2))$conf.int[1],1),
              round(fisher.test(matrix(c(P.y,P.n,C.y,C.n),
                                       nrow=2))$conf.int[2],1), sep='-')
}
results <- rbind(results, data.frame(Category='',
                                     Metadata ="Race (N & % White)",
                                     `PD N`=P.n+P.y,
                                     `PD summary stats`=paste(P.y,
                                                              " ","(",
                                                              round(P.y/(P.n+P.y)*100, 0),
                                                              "%",")",
                                                                sep=""),
                                     `NHC N`=C.n+C.y,
                                     `NHC summary stats`=paste(C.y,
                                                               " ","(",
                                                               round(C.y/(C.n+C.y)*100, 0),
                                                               "%",")",
                                                                 sep=""),
                                     `Total N`=P.n+P.y+C.n+C.y,
                                     P=formatC(p, format="e", digits=1),
                                     `OR [95%CI]`=paste(round(or, 1),' [',ci,']',sep=''),
                                     check.names=FALSE))
# jewish ancestry
P.y <- table(subset(subject.data, Case_status == "PD")$Jewish_ancestry)['Y']
P.n <- table(subset(subject.data, Case_status == "PD")$Jewish_ancestry)['N']
C.y <- table(subset(subject.data, Case_status == "Control")$Jewish_ancestry)['Y']
C.n <- table(subset(subject.data, Case_status == "Control")$Jewish_ancestry)['N']
if (is.na(P.n)){P.n <- 0}
if (is.na(P.y)){P.y <- 0}
if (is.na(C.n)){C.n <- 0}
if (is.na(C.y)){C.y <- 0}
p <- fisher.test(matrix(c(P.y,P.n,C.y,C.n), nrow=2))$p.value
if (any(c(P.y, P.n, C.y, C.n)==0)){
  or <- Prop.or(x=c(P.y,P.n), y=c(C.y,C.n), CImethod='Woolf')$estimate
  ci <- paste(round(Prop.or(x=c(P.y,P.n), y=c(C.y,C.n),
                            CImethod='Woolf')$conf.int[1],1),
              round(Prop.or(x=c(P.y,P.n), y=c(C.y,C.n),
                            CImethod='Woolf')$conf.int[2],1), sep='-')
}else{
  or <- fisher.test(matrix(c(P.y,P.n,C.y,C.n), nrow=2))$estimate
  ci <- paste(round(fisher.test(matrix(c(P.y,P.n,C.y,C.n),
```

```
                                    nrow=2))$conf.int[1],1),
              round(fisher.test(matrix(c(P.y,P.n,C.y,C.n),
                                    nrow=2))$conf.int[2],1), sep='-')
}
results <- rbind(results, data.frame(Category='',
                                    Metadata ="Jewish",
                                    `PD N`=P.n+P.y,
                                    `PD summary stats`=paste(P.y,
                                                         " ","(",
                                                         round(P.y/(P.n+P.y)*100, 0),
                                                         "%",")",
                                                          sep=""),
                                    `NHC N`=C.n+C.y,
                                    `NHC summary stats`=paste(C.y,
                                                          " ","(",
                                                          round(C.y/(C.n+C.y)*100, 0),
                                                          "%",")",
                                                           sep=""),
                                    `Total N`=P.n+P.y+C.n+C.y,
                                    P=formatC(p, format="e", digits=1),
                                    `OR [95%CI]`=paste(round(or, 1),' [',ci,']',sep=''),
                                    check.names=FALSE))
# bmi
P.t <- length(na.omit(subset(subject.data, Case_status == "PD")$BMI))
P.avg <- mean(na.omit(subset(subject.data, Case_status == "PD")$BMI))
P.sd <- sd(na.omit(subset(subject.data, Case_status == "PD")$BMI))
C.t <- length(na.omit(subset(subject.data, Case_status == "Control")$BMI))
C.avg <- mean(na.omit(subset(subject.data, Case_status == "Control")$BMI))
C.sd <- sd(na.omit(subset(subject.data, Case_status == "Control")$BMI))
p <- wilcox.test(subset(subject.data, Case_status == "PD")$BMI,
                 subset(subject.data, Case_status == "Control")$BMI)$p.value
results <- rbind(results, data.frame(Category='Weight',
                                    Metadata ="BMI",
                                    `PD N`=P.t,
                                    `PD summary stats`=paste(round(P.avg, 1),
                                                         round(P.sd, 1), sep="±"),
                                    `NHC N`=C.t,
                                    `NHC summary stats`=paste(round(C.avg, 1),
                                                          round(C.sd, 1), sep="±"),
                                    `Total N`=P.t+C.t,
                                    P=formatC(p, format="e", digits=1),
                                    `OR [95%CI]`="-",
                                    check.names=FALSE))
# lost 10lbs in past year
P.y <- table(subset(subject.data, Case_status == "PD")$Loss_10lbs_in_last_year)['Y']
P.n <- table(subset(subject.data, Case_status == "PD")$Loss_10lbs_in_last_year)['N']
C.y <- table(subset(subject.data, Case_status == "Control")$Loss_10lbs_in_last_year)['Y']
C.n <- table(subset(subject.data, Case_status == "Control")$Loss_10lbs_in_last_year)['N']
if (is.na(P.n)){P.n <- 0}
if (is.na(P.y)){P.y <- 0}
if (is.na(C.n)){C.n <- 0}
if (is.na(C.y)){C.y <- 0}
p <- fisher.test(matrix(c(P.y,P.n,C.y,C.n), nrow=2))$p.value
if (any(c(P.y, P.n, C.y, C.n)==0)){
```

```r
  or <- Prop.or(x=c(P.y,P.n), y=c(C.y,C.n), CImethod='Woolf')$estimate
  ci <- paste(round(Prop.or(x=c(P.y,P.n), y=c(C.y,C.n),
                          CImethod='Woolf')$conf.int[1],1),
              round(Prop.or(x=c(P.y,P.n), y=c(C.y,C.n),
                          CImethod='Woolf')$conf.int[2],1), sep='-')
}else{
  or <- fisher.test(matrix(c(P.y,P.n,C.y,C.n), nrow=2))$estimate
  ci <- paste(round(fisher.test(matrix(c(P.y,P.n,C.y,C.n),
                                     nrow=2))$conf.int[1],1),
              round(fisher.test(matrix(c(P.y,P.n,C.y,C.n),
                                     nrow=2))$conf.int[2],1), sep='-')
}
results <- rbind(results, data.frame(Category='',
                                     Metadata ="Lost >10 pounds in past year",
                                     `PD N`=P.n+P.y,
                                     `PD summary stats`=paste(P.y,
                                                         " ","(",
                                                         round(P.y/(P.n+P.y)*100, 0),
                                                         "%",")",
                                                           sep=""),
                                     `NHC N`=C.n+C.y,
                                     `NHC summary stats`=paste(C.y,
                                                          " ","(",
                                                          round(C.y/(C.n+C.y)*100, 0),
                                                          "%",")",
                                                            sep=""),
                                     `Total N`=P.n+P.y+C.n+C.y,
                                     P=formatC(p, format="e", digits=1),
                                     `OR [95%CI]`=paste(round(or, 1),' [',ci,']',sep=''),
                                     check.names=FALSE))
# gained 10lbs in past year
P.y <- table(subset(subject.data, Case_status == "PD")$Gained_10lbs_in_last_year)['Y']
P.n <- table(subset(subject.data, Case_status == "PD")$Gained_10lbs_in_last_year)['N']
C.y <- table(subset(subject.data, Case_status == "Control")$Gained_10lbs_in_last_year)['Y']
C.n <- table(subset(subject.data, Case_status == "Control")$Gained_10lbs_in_last_year)['N']
if (is.na(P.n)){P.n <- 0}
if (is.na(P.y)){P.y <- 0}
if (is.na(C.n)){C.n <- 0}
if (is.na(C.y)){C.y <- 0}
p <- fisher.test(matrix(c(P.y,P.n,C.y,C.n), nrow=2))$p.value
if (any(c(P.y, P.n, C.y, C.n)==0)){
  or <- Prop.or(x=c(P.y,P.n), y=c(C.y,C.n), CImethod='Woolf')$estimate
  ci <- paste(round(Prop.or(x=c(P.y,P.n), y=c(C.y,C.n),
                          CImethod='Woolf')$conf.int[1],1),
              round(Prop.or(x=c(P.y,P.n), y=c(C.y,C.n),
                          CImethod='Woolf')$conf.int[2],1), sep='-')
}else{
  or <- fisher.test(matrix(c(P.y,P.n,C.y,C.n), nrow=2))$estimate
  ci <- paste(round(fisher.test(matrix(c(P.y,P.n,C.y,C.n),
                                     nrow=2))$conf.int[1],1),
              round(fisher.test(matrix(c(P.y,P.n,C.y,C.n),
                                     nrow=2))$conf.int[2],1), sep='-')
}
results <- rbind(results, data.frame(Category='',
```

```
                                     Metadata ="Gained >10 pounds in past year",
                                     `PD N`=P.n+P.y,
                                     `PD summary stats`=paste(P.y,
                                                              " ","(",
                                                              round(P.y/(P.n+P.y)*100, 0),
                                                              "%",")",
                                                                sep=""),
                                     `NHC N`=C.n+C.y,
                                     `NHC summary stats`=paste(C.y,
                                                               " ","(",
                                                               round(C.y/(C.n+C.y)*100, 0),
                                                               "%",")",
                                                                 sep=""),
                                     `Total N`=P.n+P.y+C.n+C.y,
                                     P=formatC(p, format="e", digits=1),
                                     `OR [95%CI]`=paste(round(or, 1),' [',ci,']',sep=''),
                                     check.names=FALSE))
# fruits or vegetables daily
P.y <- table(subset(subject.data,
     Case_status == "PD")$How_often_do_you_eat_FRUITS_or_VEGETABLES)["At least once a day"]
P.n <- sum(table(subset(subject.data,
     Case_status == "PD")$How_often_do_you_eat_FRUITS_or_VEGETABLES))-P.y
C.y <- table(subset(subject.data,
     Case_status == "Control")$How_often_do_you_eat_FRUITS_or_VEGETABLES)["At least once a day"]
C.n <- sum(table(subset(subject.data,
     Case_status == "Control")$How_often_do_you_eat_FRUITS_or_VEGETABLES))-C.y
if (is.na(P.n)){P.n <- 0}
if (is.na(P.y)){P.y <- 0}
if (is.na(C.n)){C.n <- 0}
if (is.na(C.y)){C.y <- 0}
p <- fisher.test(matrix(c(P.y,P.n,C.y,C.n), nrow=2))$p.value
if (any(c(P.y, P.n, C.y, C.n)==0)){
  or <- Prop.or(x=c(P.y,P.n), y=c(C.y,C.n), CImethod='Woolf')$estimate
  ci <- paste(round(Prop.or(x=c(P.y,P.n), y=c(C.y,C.n),
                            CImethod='Woolf')$conf.int[1],1),
              round(Prop.or(x=c(P.y,P.n), y=c(C.y,C.n),
                            CImethod='Woolf')$conf.int[2],1), sep='-')
}else{
  or <- fisher.test(matrix(c(P.y,P.n,C.y,C.n), nrow=2))$estimate
  ci <- paste(round(fisher.test(matrix(c(P.y,P.n,C.y,C.n),
                                       nrow=2))$conf.int[1],1),
              round(fisher.test(matrix(c(P.y,P.n,C.y,C.n),
                                       nrow=2))$conf.int[2],1), sep='-')
}
results <- rbind(results, data.frame(Category='Diet',
                                     Metadata ="Fruits or vegetables daily",
                                     `PD N`=P.n+P.y,
                                     `PD summary stats`=paste(P.y,
                                                              " ","(",
                                                              round(P.y/(P.n+P.y)*100, 0),
                                                              "%",")",
                                                                sep=""),
                                     `NHC N`=C.n+C.y,
                                     `NHC summary stats`=paste(C.y,
```

```
                                                    " ","(",
                                                    round(C.y/(C.n+C.y)*100, 0),
                                                    "%",")",
                                                      sep=""),
                                    `Total N`=P.n+P.y+C.n+C.y,
                                    P=formatC(p, format="e", digits=1),
                                    `OR [95%CI]`=paste(round(or, 1),' [',ci,']',sep=''),
                                    check.names=FALSE))
# poultry, beef, pork, seafood, eggs daily
P.y <- table(subset(subject.data,
  Case_status == "PD")$How_often_do_you_eat_POULTRY_BEEF_PORK_SEAFOOD_EGGS)["At least once a day"]
P.n <- sum(table(subset(subject.data,
  Case_status == "PD")$How_often_do_you_eat_POULTRY_BEEF_PORK_SEAFOOD_EGGS))-P.y
C.y <- table(subset(subject.data,
  Case_status == "Control")$How_often_do_you_eat_POULTRY_BEEF_PORK_SEAFOOD_EGGS)["At least once a day"]
C.n <- sum(table(subset(subject.data,
  Case_status == "Control")$How_often_do_you_eat_POULTRY_BEEF_PORK_SEAFOOD_EGGS))-C.y
if (is.na(P.n)){P.n <- 0}
if (is.na(P.y)){P.y <- 0}
if (is.na(C.n)){C.n <- 0}
if (is.na(C.y)){C.y <- 0}
p <- fisher.test(matrix(c(P.y,P.n,C.y,C.n), nrow=2))$p.value
or <- fisher.test(matrix(c(P.y,P.n,C.y,C.n), nrow=2))$estimate
if (any(c(P.y, P.n, C.y, C.n)==0)){
  or <- Prop.or(x=c(P.y,P.n), y=c(C.y,C.n), CImethod='Woolf')$estimate
  ci <- paste(round(Prop.or(x=c(P.y,P.n), y=c(C.y,C.n),
                              CImethod='Woolf')$conf.int[1],1),
              round(Prop.or(x=c(P.y,P.n), y=c(C.y,C.n),
                              CImethod='Woolf')$conf.int[2],1), sep='-')
}else{
  or <- fisher.test(matrix(c(P.y,P.n,C.y,C.n), nrow=2))$estimate
  ci <- paste(round(fisher.test(matrix(c(P.y,P.n,C.y,C.n),
                                         nrow=2))$conf.int[1],1),
              round(fisher.test(matrix(c(P.y,P.n,C.y,C.n),
                                         nrow=2))$conf.int[2],1), sep='-')
}
results <- rbind(results, data.frame(Category='',
                                    Metadata ="Poultry, beef, pork, seafood, eggs daily",
                                    `PD N`=P.n+P.y,
                                    `PD summary stats`=paste(P.y,
                                                   " ","(",
                                                   round(P.y/(P.n+P.y)*100, 0),
                                                   "%",")",
                                                     sep=""),
                                    `NHC N`=C.n+C.y,
                                    `NHC summary stats`=paste(C.y,
                                                    " ","(",
                                                    round(C.y/(C.n+C.y)*100, 0),
                                                    "%",")",
                                                      sep=""),
                                    `Total N`=P.n+P.y+C.n+C.y,
                                    P=formatC(p, format="e", digits=1),
                                    `OR [95%CI]`=paste(round(or, 1),' [',ci,']',sep=''),
                                    check.names=FALSE))
```

```
# nuts daily
P.y <- table(subset(subject.data,
      Case_status == "PD")$How_often_do_you_eat_NUTS)["At least once a day"]
P.n <- sum(table(subset(subject.data,
      Case_status == "PD")$How_often_do_you_eat_NUTS))-P.y
C.y <- table(subset(subject.data,
      Case_status == "Control")$How_often_do_you_eat_NUTS)["At least once a day"]
C.n <- sum(table(subset(subject.data,
      Case_status == "Control")$How_often_do_you_eat_NUTS))-C.y
if (is.na(P.n)){P.n <- 0}
if (is.na(P.y)){P.y <- 0}
if (is.na(C.n)){C.n <- 0}
if (is.na(C.y)){C.y <- 0}
p <- fisher.test(matrix(c(P.y,P.n,C.y,C.n), nrow=2))$p.value
if (any(c(P.y, P.n, C.y, C.n)==0)){
  or <- Prop.or(x=c(P.y,P.n), y=c(C.y,C.n), CImethod='Woolf')$estimate
  ci <- paste(round(Prop.or(x=c(P.y,P.n), y=c(C.y,C.n),
                            CImethod='Woolf')$conf.int[1],1),
              round(Prop.or(x=c(P.y,P.n), y=c(C.y,C.n),
                            CImethod='Woolf')$conf.int[2],1), sep='-')
}else{
  or <- fisher.test(matrix(c(P.y,P.n,C.y,C.n), nrow=2))$estimate
  ci <- paste(round(fisher.test(matrix(c(P.y,P.n,C.y,C.n),
                                       nrow=2))$conf.int[1],1),
              round(fisher.test(matrix(c(P.y,P.n,C.y,C.n),
                                       nrow=2))$conf.int[2],1), sep='-')
}
results <- rbind(results, data.frame(Category='',
                                     Metadata ="Nuts daily",
                                     `PD N`=P.n+P.y,
                                     `PD summary stats`=paste(P.y,
                                                              " ","(",
                                                              round(P.y/(P.n+P.y)*100, 0),
                                                              "%",")",
                                                                sep=""),
                                     `NHC N`=C.n+C.y,
                                     `NHC summary stats`=paste(C.y,
                                                               " ","(",
                                                               round(C.y/(C.n+C.y)*100, 0),
                                                               "%",")",
                                                                 sep=""),
                                     `Total N`=P.n+P.y+C.n+C.y,
                                     P=formatC(p, format="e", digits=1),
                                     `OR [95%CI]`=paste(round(or, 1),' [',ci,']',sep=''),
                                     check.names=FALSE))
# yogurt at least a few times a week
P.y <- table(subset(subject.data,
      Case_status == "PD")$How_often_do_you_eat_YOGURT)["At least once a day"]+
        table(subset(subject.data,
      Case_status == "PD")$How_often_do_you_eat_YOGURT)["Few times a week"]
P.n <- sum(table(subset(subject.data,
      Case_status == "PD")$How_often_do_you_eat_YOGURT))-P.y
C.y <- table(subset(subject.data,
      Case_status == "Control")$How_often_do_you_eat_YOGURT)["At least once a day"]+
```

```
       table(subset(subject.data,
     Case_status == "Control")$How_often_do_you_eat_YOGURT)["Few times a week"]
C.n <- sum(table(subset(subject.data,
     Case_status == "Control")$How_often_do_you_eat_YOGURT))-C.y
if (is.na(P.n)){P.n <- 0}
if (is.na(P.y)){P.y <- 0}
if (is.na(C.n)){C.n <- 0}
if (is.na(C.y)){C.y <- 0}
p <- fisher.test(matrix(c(P.y,P.n,C.y,C.n), nrow=2))$p.value
if (any(c(P.y, P.n, C.y, C.n)==0)){
  or <- Prop.or(x=c(P.y,P.n), y=c(C.y,C.n), CImethod='Woolf')$estimate
  ci <- paste(round(Prop.or(x=c(P.y,P.n), y=c(C.y,C.n),
                     CImethod='Woolf')$conf.int[1],1),
              round(Prop.or(x=c(P.y,P.n), y=c(C.y,C.n),
                     CImethod='Woolf')$conf.int[2],1), sep='-')
}else{
  or <- fisher.test(matrix(c(P.y,P.n,C.y,C.n), nrow=2))$estimate
  ci <- paste(round(fisher.test(matrix(c(P.y,P.n,C.y,C.n),
                                nrow=2))$conf.int[1],1),
              round(fisher.test(matrix(c(P.y,P.n,C.y,C.n),
                                nrow=2))$conf.int[2],1), sep='-')
}
results <- rbind(results, data.frame(Category='',
                                     Metadata ="Yogurt at least a few times a week",
                                     `PD N`=P.n+P.y,
                                     `PD summary stats`=paste(P.y,
                                                              " ","(",
                                                              round(P.y/(P.n+P.y)*100, 0),
                                                              "%",")",
                                                                sep=""),
                                     `NHC N`=C.n+C.y,
                                     `NHC summary stats`=paste(C.y,
                                                               " ","(",
                                                               round(C.y/(C.n+C.y)*100, 0),
                                                               "%",")",
                                                                 sep=""),
                                     `Total N`=P.n+P.y+C.n+C.y,
                                     P=formatC(p, format="e", digits=1),
                                     `OR [95%CI]`=paste(round(or, 1),' [',ci,']',sep=''),
                                     check.names=FALSE))
# grains daily
P.y <- table(subset(subject.data,
     Case_status == "PD")$How_often_do_you_eat_GRAINS)["At least once a day"]
P.n <- sum(table(subset(subject.data,
     Case_status == "PD")$How_often_do_you_eat_GRAINS))-P.y
C.y <- table(subset(subject.data,
     Case_status == "Control")$How_often_do_you_eat_GRAINS)["At least once a day"]
C.n <- sum(table(subset(subject.data,
     Case_status == "Control")$How_often_do_you_eat_GRAINS))-C.y
if (is.na(P.n)){P.n <- 0}
if (is.na(P.y)){P.y <- 0}
if (is.na(C.n)){C.n <- 0}
if (is.na(C.y)){C.y <- 0}
p <- fisher.test(matrix(c(P.y,P.n,C.y,C.n), nrow=2))$p.value
```

```
if (any(c(P.y, P.n, C.y, C.n)==0)){
  or <- Prop.or(x=c(P.y,P.n), y=c(C.y,C.n), CImethod='Woolf')$estimate
  ci <- paste(round(Prop.or(x=c(P.y,P.n), y=c(C.y,C.n),
                            CImethod='Woolf')$conf.int[1],1),
              round(Prop.or(x=c(P.y,P.n), y=c(C.y,C.n),
                            CImethod='Woolf')$conf.int[2],1), sep='-')
}else{
  or <- fisher.test(matrix(c(P.y,P.n,C.y,C.n), nrow=2))$estimate
  ci <- paste(round(fisher.test(matrix(c(P.y,P.n,C.y,C.n),
                                       nrow=2))$conf.int[1],1),
              round(fisher.test(matrix(c(P.y,P.n,C.y,C.n),
                                       nrow=2))$conf.int[2],1), sep='-')
}
results <- rbind(results, data.frame(Category='',
                                     Metadata ="Grains daily",
                                     `PD N`=P.n+P.y,
                                     `PD summary stats`=paste(P.y,
                                                              " ","(",
                                                              round(P.y/(P.n+P.y)*100, 0),
                                                              "%",")",
                                                                sep=""),
                                     `NHC N`=C.n+C.y,
                                     `NHC summary stats`=paste(C.y,
                                                               " ","(",
                                                               round(C.y/(C.n+C.y)*100, 0),
                                                               "%",")",
                                                                 sep=""),
                                     `Total N`=P.n+P.y+C.n+C.y,
                                     P=formatC(p, format="e", digits=1),
                                     `OR [95%CI]`=paste(round(or, 1),' [',ci,']',sep=''),
                                     check.names=FALSE))
# alcohol
P.y <- table(subset(subject.data, Case_status == "PD")$Do_you_drink_alcohol)['Y']
P.n <- table(subset(subject.data, Case_status == "PD")$Do_you_drink_alcohol)['N']
C.y <- table(subset(subject.data, Case_status == "Control")$Do_you_drink_alcohol)['Y']
C.n <- table(subset(subject.data, Case_status == "Control")$Do_you_drink_alcohol)['N']
if (is.na(P.n)){P.n <- 0}
if (is.na(P.y)){P.y <- 0}
if (is.na(C.n)){C.n <- 0}
if (is.na(C.y)){C.y <- 0}
p <- fisher.test(matrix(c(P.y,P.n,C.y,C.n), nrow=2))$p.value
if (any(c(P.y, P.n, C.y, C.n)==0)){
  or <- Prop.or(x=c(P.y,P.n), y=c(C.y,C.n), CImethod='Woolf')$estimate
  ci <- paste(round(Prop.or(x=c(P.y,P.n), y=c(C.y,C.n),
                            CImethod='Woolf')$conf.int[1],1),
              round(Prop.or(x=c(P.y,P.n), y=c(C.y,C.n),
                            CImethod='Woolf')$conf.int[2],1), sep='-')
}else{
  or <- fisher.test(matrix(c(P.y,P.n,C.y,C.n), nrow=2))$estimate
  ci <- paste(round(fisher.test(matrix(c(P.y,P.n,C.y,C.n),
                                       nrow=2))$conf.int[1],1),
              round(fisher.test(matrix(c(P.y,P.n,C.y,C.n),
                                       nrow=2))$conf.int[2],1), sep='-')
}
```

```
results <- rbind(results, data.frame(Category='',
                                     Metadata ="Alcohol",
                                     `PD N`=P.n+P.y,
                                     `PD summary stats`=paste(P.y,
                                                              " ","(",
                                                              round(P.y/(P.n+P.y)*100, 0),
                                                              "%",")",
                                                                sep=""),
                                     `NHC N`=C.n+C.y,
                                     `NHC summary stats`=paste(C.y,
                                                               " ","(",
                                                               round(C.y/(C.n+C.y)*100, 0),
                                                               "%",")",
                                                                 sep=""),
                                     `Total N`=P.n+P.y+C.n+C.y,
                                     P=formatC(p, format="e", digits=1),
                                     `OR [95%CI]`=paste(round(or, 1),' [',ci,']',sep=''),
                                     check.names=FALSE))
# do you smoke
P.y <- table(subset(subject.data, Case_status == "PD")$Do_you_smoke)['Y']
P.n <- table(subset(subject.data, Case_status == "PD")$Do_you_smoke)['N']
C.y <- table(subset(subject.data, Case_status == "Control")$Do_you_smoke)['Y']
C.n <- table(subset(subject.data, Case_status == "Control")$Do_you_smoke)['N']
if (is.na(P.n)){P.n <- 0}
if (is.na(P.y)){P.y <- 0}
if (is.na(C.n)){C.n <- 0}
if (is.na(C.y)){C.y <- 0}
p <- fisher.test(matrix(c(P.y,P.n,C.y,C.n), nrow=2))$p.value
if (any(c(P.y, P.n, C.y, C.n)==0)){
  or <- Prop.or(x=c(P.y,P.n), y=c(C.y,C.n), CImethod='Woolf')$estimate
  ci <- paste(round(Prop.or(x=c(P.y,P.n), y=c(C.y,C.n),
                            CImethod='Woolf')$conf.int[1],1),
              round(Prop.or(x=c(P.y,P.n), y=c(C.y,C.n),
                            CImethod='Woolf')$conf.int[2],1), sep='-')
}else{
  or <- fisher.test(matrix(c(P.y,P.n,C.y,C.n), nrow=2))$estimate
  ci <- paste(round(fisher.test(matrix(c(P.y,P.n,C.y,C.n),
                                       nrow=2))$conf.int[1],1),
              round(fisher.test(matrix(c(P.y,P.n,C.y,C.n),
                                       nrow=2))$conf.int[2],1), sep='-')
}
results <- rbind(results, data.frame(Category='',
                                     Metadata ="Tobacco",
                                     `PD N`=P.n+P.y,
                                     `PD summary stats`=paste(P.y,
                                                              " ","(",
                                                              round(P.y/(P.n+P.y)*100, 0),
                                                              "%",")",
                                                                sep=""),
                                     `NHC N`=C.n+C.y,
                                     `NHC summary stats`=paste(C.y,
                                                               " ","(",
                                                               round(C.y/(C.n+C.y)*100, 0),
                                                               "%",")",
```

```
                                                        sep=""),
                                    `Total N`=P.n+P.y+C.n+C.y,
                                    P=formatC(p, format="e", digits=1),
                                    `OR [95%CI]`=paste(round(or, 1),' [',ci,']',sep=''),
                                    check.names=FALSE))
# caffeine
P.y <- table(subset(subject.data,
     Case_status == "PD")$Do_you_drink_caffeinated_beverages)['Y']
P.n <- table(subset(subject.data,
     Case_status == "PD")$Do_you_drink_caffeinated_beverages)['N']
C.y <- table(subset(subject.data,
     Case_status == "Control")$Do_you_drink_caffeinated_beverages)['Y']
C.n <- table(subset(subject.data,
     Case_status == "Control")$Do_you_drink_caffeinated_beverages)['N']
if (is.na(P.n)){P.n <- 0}
if (is.na(P.y)){P.y <- 0}
if (is.na(C.n)){C.n <- 0}
if (is.na(C.y)){C.y <- 0}
p <- fisher.test(matrix(c(P.y,P.n,C.y,C.n), nrow=2))$p.value
if (any(c(P.y, P.n, C.y, C.n)==0)){
  or <- Prop.or(x=c(P.y,P.n), y=c(C.y,C.n), CImethod='Woolf')$estimate
  ci <- paste(round(Prop.or(x=c(P.y,P.n), y=c(C.y,C.n),
                    CImethod='Woolf')$conf.int[1],1),
              round(Prop.or(x=c(P.y,P.n), y=c(C.y,C.n),
                    CImethod='Woolf')$conf.int[2],1), sep='-')
}else{
  or <- fisher.test(matrix(c(P.y,P.n,C.y,C.n), nrow=2))$estimate
  ci <- paste(round(fisher.test(matrix(c(P.y,P.n,C.y,C.n),
                               nrow=2))$conf.int[1],1),
              round(fisher.test(matrix(c(P.y,P.n,C.y,C.n),
                               nrow=2))$conf.int[2],1), sep='-')
}
results <- rbind(results, data.frame(Category='',
                                    Metadata ="Caffeine",
                                    `PD N`=P.n+P.y,
                                    `PD summary stats`=paste(P.y,
                                                       " ","(",
                                                       round(P.y/(P.n+P.y)*100, 0),
                                                       "%",")",
                                                         sep=""),
                                    `NHC N`=C.n+C.y,
                                    `NHC summary stats`=paste(C.y,
                                                        " ","(",
                                                        round(C.y/(C.n+C.y)*100, 0),
                                                        "%",")",
                                                          sep=""),
                                    `Total N`=P.n+P.y+C.n+C.y,
                                    P=formatC(p, format="e", digits=1),
                                    `OR [95%CI]`=paste(round(or, 1),' [',ci,']',sep=''),
                                    check.names=FALSE))
# constipation day of stool collection
P.y <- table(subset(subject.data,
     Case_status == "PD")$Day_of_stool_collection_constipation)['Y']
P.n <- table(subset(subject.data,
```

```
      Case_status == "PD")$Day_of_stool_collection_constipation)['N']
C.y <- table(subset(subject.data,
      Case_status == "Control")$Day_of_stool_collection_constipation)['Y']
C.n <- table(subset(subject.data,
      Case_status == "Control")$Day_of_stool_collection_constipation)['N']
if (is.na(P.n)){P.n <- 0}
if (is.na(P.y)){P.y <- 0}
if (is.na(C.n)){C.n <- 0}
if (is.na(C.y)){C.y <- 0}
p <- fisher.test(matrix(c(P.y,P.n,C.y,C.n), nrow=2))$p.value
if (any(c(P.y, P.n, C.y, C.n)==0)){
  or <- Prop.or(x=c(P.y,P.n), y=c(C.y,C.n), CImethod='Woolf')$estimate
  ci <- paste(round(Prop.or(x=c(P.y,P.n), y=c(C.y,C.n),
                            CImethod='Woolf')$conf.int[1],1),
              round(Prop.or(x=c(P.y,P.n), y=c(C.y,C.n),
                            CImethod='Woolf')$conf.int[2],1), sep='-')
}else{
  or <- fisher.test(matrix(c(P.y,P.n,C.y,C.n), nrow=2))$estimate
  ci <- paste(round(fisher.test(matrix(c(P.y,P.n,C.y,C.n),
                                       nrow=2))$conf.int[1],1),
              round(fisher.test(matrix(c(P.y,P.n,C.y,C.n),
                                       nrow=2))$conf.int[2],1), sep='-')
}
results <- rbind(results,
              data.frame(Category='GI health on day of stool collection',
                  Metadata ="Constipation (no bowel movement) in >=3 days prior to stool collection",
                     `PD N`=P.n+P.y,
                     `PD summary stats`=paste(P.y,
                                              " ","(",
                                              round(P.y/(P.n+P.y)*100, 0),
                                              "%",")",
                                              sep=""),
                     `NHC N`=C.n+C.y,
                     `NHC summary stats`=paste(C.y,
                                               " ","(",
                                               round(C.y/(C.n+C.y)*100, 0),
                                               "%",")",
                                               sep=""),
                      `Total N`=P.n+P.y+C.n+C.y,
                      P=formatC(p, format="e", digits=1),
                      `OR [95%CI]`=paste(round(or, 1),' [',ci,']',sep=''),
                      check.names=FALSE))
# diarrhea day of stool collection
P.y <- table(subset(subject.data,
      Case_status == "PD")$Day_of_stool_collection_diarrhea)['Y']
P.n <- table(subset(subject.data,
      Case_status == "PD")$Day_of_stool_collection_diarrhea)['N']
C.y <- table(subset(subject.data,
      Case_status == "Control")$Day_of_stool_collection_diarrhea)['Y']
C.n <- table(subset(subject.data,
      Case_status == "Control")$Day_of_stool_collection_diarrhea)['N']
if (is.na(P.n)){P.n <- 0}
if (is.na(P.y)){P.y <- 0}
if (is.na(C.n)){C.n <- 0}
```

```r
if (is.na(C.y)){C.y <- 0}
p <- fisher.test(matrix(c(P.y,P.n,C.y,C.n), nrow=2))$p.value
if (any(c(P.y, P.n, C.y, C.n)==0)){
  or <- Prop.or(x=c(P.y,P.n), y=c(C.y,C.n), CImethod='Woolf')$estimate
  ci <- paste(round(Prop.or(x=c(P.y,P.n), y=c(C.y,C.n),
                            CImethod='Woolf')$conf.int[1],1),
              round(Prop.or(x=c(P.y,P.n), y=c(C.y,C.n),
                            CImethod='Woolf')$conf.int[2],1), sep='-')
}else{
  or <- fisher.test(matrix(c(P.y,P.n,C.y,C.n), nrow=2))$estimate
  ci <- paste(round(fisher.test(matrix(c(P.y,P.n,C.y,C.n),
                                       nrow=2))$conf.int[1],1),
              round(fisher.test(matrix(c(P.y,P.n,C.y,C.n),
                                       nrow=2))$conf.int[2],1), sep='-')
}
results <- rbind(results, data.frame(Category='',
                                     Metadata ="Diarrhea",
                                     `PD N`=P.n+P.y,
                                     `PD summary stats`=paste(P.y,
                                                         " ","(",
                                                         round(P.y/(P.n+P.y)*100, 0),
                                                         "%",")",
                                                           sep=""),
                                     `NHC N`=C.n+C.y,
                                     `NHC summary stats`=paste(C.y,
                                                          " ","(",
                                                          round(C.y/(C.n+C.y)*100, 0),
                                                          "%",")",
                                                           sep=""),
                                     `Total N`=P.n+P.y+C.n+C.y,
                                     P=formatC(p, format="e", digits=1),
                                     `OR [95%CI]`=paste(round(or, 1),' [',ci,']',sep=''),
                                     check.names=FALSE))
# abdominal pain day of stool collection
P.y <- table(subset(subject.data,
     Case_status == "PD")$Day_of_stool_collection_abdominal_pain)['Y']
P.n <- table(subset(subject.data,
     Case_status == "PD")$Day_of_stool_collection_abdominal_pain)['N']
C.y <- table(subset(subject.data,
     Case_status == "Control")$Day_of_stool_collection_abdominal_pain)['Y']
C.n <- table(subset(subject.data,
     Case_status == "Control")$Day_of_stool_collection_abdominal_pain)['N']
if (is.na(P.n)){P.n <- 0}
if (is.na(P.y)){P.y <- 0}
if (is.na(C.n)){C.n <- 0}
if (is.na(C.y)){C.y <- 0}
p <- fisher.test(matrix(c(P.y,P.n,C.y,C.n), nrow=2))$p.value
if (any(c(P.y, P.n, C.y, C.n)==0)){
  or <- Prop.or(x=c(P.y,P.n), y=c(C.y,C.n), CImethod='Woolf')$estimate
  ci <- paste(round(Prop.or(x=c(P.y,P.n), y=c(C.y,C.n),
                            CImethod='Woolf')$conf.int[1],1),
              round(Prop.or(x=c(P.y,P.n), y=c(C.y,C.n),
                            CImethod='Woolf')$conf.int[2],1), sep='-')
}else{
```

```
  or <- fisher.test(matrix(c(P.y,P.n,C.y,C.n), nrow=2))$estimate
  ci <- paste(round(fisher.test(matrix(c(P.y,P.n,C.y,C.n),
                                    nrow=2))$conf.int[1],1),
              round(fisher.test(matrix(c(P.y,P.n,C.y,C.n),
                                    nrow=2))$conf.int[2],1), sep='-')
}
results <- rbind(results, data.frame(Category='',
                                     Metadata ="Abdominal pain",
                                     `PD N`=P.n+P.y,
                                     `PD summary stats`=paste(P.y,
                                                         " ","(",
                                                         round(P.y/(P.n+P.y)*100, 0),
                                                         "%",")",
                                                           sep=""),
                                     `NHC N`=C.n+C.y,
                                     `NHC summary stats`=paste(C.y,
                                                          " ","(",
                                                          round(C.y/(C.n+C.y)*100, 0),
                                                          "%",")",
                                                            sep=""),
                                     `Total N`=P.n+P.y+C.n+C.y,
                                     P=formatC(p, format="e", digits=1),
                                     `OR [95%CI]`=paste(round(or, 1),' [',ci,']',sep=''),
                                     check.names=FALSE))
# excess gas day of stool collection
P.y <- table(subset(subject.data,
     Case_status == "PD")$Day_of_stool_collection_excess_gas)['Y']
P.n <- table(subset(subject.data,
     Case_status == "PD")$Day_of_stool_collection_excess_gas)['N']
C.y <- table(subset(subject.data,
     Case_status == "Control")$Day_of_stool_collection_excess_gas)['Y']
C.n <- table(subset(subject.data,
     Case_status == "Control")$Day_of_stool_collection_excess_gas)['N']
if (is.na(P.n)){P.n <- 0}
if (is.na(P.y)){P.y <- 0}
if (is.na(C.n)){C.n <- 0}
if (is.na(C.y)){C.y <- 0}
p <- fisher.test(matrix(c(P.y,P.n,C.y,C.n), nrow=2))$p.value
if (any(c(P.y, P.n, C.y, C.n)==0)){
  or <- Prop.or(x=c(P.y,P.n), y=c(C.y,C.n), CImethod='Woolf')$estimate
  ci <- paste(round(Prop.or(x=c(P.y,P.n), y=c(C.y,C.n),
                         CImethod='Woolf')$conf.int[1],1),
              round(Prop.or(x=c(P.y,P.n), y=c(C.y,C.n),
                         CImethod='Woolf')$conf.int[2],1), sep='-')
}else{
  or <- fisher.test(matrix(c(P.y,P.n,C.y,C.n), nrow=2))$estimate
  ci <- paste(round(fisher.test(matrix(c(P.y,P.n,C.y,C.n),
                                    nrow=2))$conf.int[1],1),
              round(fisher.test(matrix(c(P.y,P.n,C.y,C.n),
                                    nrow=2))$conf.int[2],1), sep='-')
}
results <- rbind(results, data.frame(Category='',
                                     Metadata ="Excess gas",
                                     `PD N`=P.n+P.y,
```

```
                                     `PD summary stats`=paste(P.y,
                                                         " ","(",
                                                         round(P.y/(P.n+P.y)*100, 0),
                                                         "%",")",
                                                           sep=""),
                                     `NHC N`=C.n+C.y,
                                     `NHC summary stats`=paste(C.y,
                                                          " ","(",
                                                          round(C.y/(C.n+C.y)*100, 0),
                                                          "%",")",
                                                           sep=""),
                                     `Total N`=P.n+P.y+C.n+C.y,
                                     P=formatC(p, format="e", digits=1),
                                     `OR [95%CI]`=paste(round(or, 1),' [',ci,']',sep=''),
                                     check.names=FALSE))
# bloating day of stool collection
P.y <- table(subset(subject.data,
     Case_status == "PD")$Day_of_stool_collection_bloating)['Y']
P.n <- table(subset(subject.data,
     Case_status == "PD")$Day_of_stool_collection_bloating)['N']
C.y <- table(subset(subject.data,
     Case_status == "Control")$Day_of_stool_collection_bloating)['Y']
C.n <- table(subset(subject.data,
     Case_status == "Control")$Day_of_stool_collection_bloating)['N']
if (is.na(P.n)){P.n <- 0}
if (is.na(P.y)){P.y <- 0}
if (is.na(C.n)){C.n <- 0}
if (is.na(C.y)){C.y <- 0}
p <- fisher.test(matrix(c(P.y,P.n,C.y,C.n), nrow=2))$p.value
if (any(c(P.y, P.n, C.y, C.n)==0)){
  or <- Prop.or(x=c(P.y,P.n), y=c(C.y,C.n), CImethod='Woolf')$estimate
  ci <- paste(round(Prop.or(x=c(P.y,P.n), y=c(C.y,C.n),
                            CImethod='Woolf')$conf.int[1],1),
              round(Prop.or(x=c(P.y,P.n), y=c(C.y,C.n),
                            CImethod='Woolf')$conf.int[2],1), sep='-')
}else{
  or <- fisher.test(matrix(c(P.y,P.n,C.y,C.n), nrow=2))$estimate
  ci <- paste(round(fisher.test(matrix(c(P.y,P.n,C.y,C.n),
                                       nrow=2))$conf.int[1],1),
              round(fisher.test(matrix(c(P.y,P.n,C.y,C.n),
                                       nrow=2))$conf.int[2],1), sep='-')
}
results <- rbind(results, data.frame(Category='',
                                     Metadata ="Bloating",
                                     `PD N`=P.n+P.y,
                                     `PD summary stats`=paste(P.y,
                                                         " ","(",
                                                         round(P.y/(P.n+P.y)*100, 0),
                                                         "%",")",
                                                           sep=""),
                                     `NHC N`=C.n+C.y,
                                     `NHC summary stats`=paste(C.y,
                                                          " ","(",
                                                          round(C.y/(C.n+C.y)*100, 0),
```

```
                                                         "%",")",
                                                         sep=""),
                                         `Total N`=P.n+P.y+C.n+C.y,
                                         P=formatC(p, format="e", digits=1),
                                         `OR [95%CI]`=paste(round(or, 1),' [',ci,']',sep=''),
                                         check.names=FALSE))
# GI discomfort day of stool collection
P.y <- table(subset(subject.data,
     Case_status == "PD")$Day_of_stool_collection_digestion_issue)['Y']
P.n <- table(subset(subject.data,
     Case_status == "PD")$Day_of_stool_collection_digestion_issue)['N']
C.y <- table(subset(subject.data,
     Case_status == "Control")$Day_of_stool_collection_digestion_issue)['Y']
C.n <- table(subset(subject.data,
     Case_status == "Control")$Day_of_stool_collection_digestion_issue)['N']
if (is.na(P.n)){P.n <- 0}
if (is.na(P.y)){P.y <- 0}
if (is.na(C.n)){C.n <- 0}
if (is.na(C.y)){C.y <- 0}
p <- fisher.test(matrix(c(P.y,P.n,C.y,C.n), nrow=2))$p.value
if (any(c(P.y, P.n, C.y, C.n)==0)){
  or <- Prop.or(x=c(P.y,P.n), y=c(C.y,C.n), CImethod='Woolf')$estimate
  ci <- paste(round(Prop.or(x=c(P.y,P.n), y=c(C.y,C.n),
                          CImethod='Woolf')$conf.int[1],1),
              round(Prop.or(x=c(P.y,P.n), y=c(C.y,C.n),
                          CImethod='Woolf')$conf.int[2],1), sep='-')
}else{
  or <- fisher.test(matrix(c(P.y,P.n,C.y,C.n), nrow=2))$estimate
  ci <- paste(round(fisher.test(matrix(c(P.y,P.n,C.y,C.n),
                                     nrow=2))$conf.int[1],1),
              round(fisher.test(matrix(c(P.y,P.n,C.y,C.n),
                                     nrow=2))$conf.int[2],1), sep='-')
}
results <- rbind(results,
    data.frame(Category='',
        Metadata ="GI discomfort on day of stool collection (yes to any of the five items)",
        `PD N`=P.n+P.y,
        `PD summary stats`=paste(P.y,
                                " ","(",
                                round(P.y/(P.n+P.y)*100, 0),
                                "%",")",
                                sep=""),
        `NHC N`=C.n+C.y,
        `NHC summary stats`=paste(C.y,
                                 " ","(",
                                 round(C.y/(C.n+C.y)*100, 0),
                                 "%",")",
                                 sep=""),
        `Total N`=P.n+P.y+C.n+C.y,
        P=formatC(p, format="e", digits=1),
                  `OR [95%CI]`=paste(round(or, 1),' [',ci,']',sep=''),
        check.names=FALSE))
# bristol stool chart
P.t <- length(na.omit(subset(subject.data, Case_status == "PD")$Bristol_stool_chart))
```

```
P.avg <- mean(na.omit(subset(subject.data, Case_status == "PD")$Bristol_stool_chart))
P.sd <- sd(na.omit(subset(subject.data, Case_status == "PD")$Bristol_stool_chart))
C.t <- length(na.omit(subset(subject.data, Case_status == "Control")$Bristol_stool_chart))
C.avg <- mean(na.omit(subset(subject.data, Case_status == "Control")$Bristol_stool_chart))
C.sd <- sd(na.omit(subset(subject.data, Case_status == "Control")$Bristol_stool_chart))
p <- wilcox.test(subset(subject.data, Case_status == "PD")$Bristol_stool_chart,
                 subset(subject.data, Case_status == "Control")$Bristol_stool_chart)$p.value
results <- rbind(results, data.frame(Category='',
                                     Metadata ="Bristol stool chart",
                                     `PD N`=P.t,
                                     `PD summary stats`=paste(round(P.avg, 1),
                                                              round(P.sd, 1), sep="±"),
                                     `NHC N`=C.t,
                                     `NHC summary stats`=paste(round(C.avg, 1),
                                                               round(C.sd, 1), sep="±"),
                                     `Total N`=P.t+C.t,
                                     P=formatC(p, format="e", digits=1),
                                     `OR [95%CI]`="-", check.names=FALSE))
# constipation in the past 3 months
P.y <- table(subset(subject.data, Case_status == "PD")$Constipation)['Y']
P.n <- table(subset(subject.data, Case_status == "PD")$Constipation)['N']
C.y <- table(subset(subject.data, Case_status == "Control")$Constipation)['Y']
C.n <- table(subset(subject.data, Case_status == "Control")$Constipation)['N']
if (is.na(P.n)){P.n <- 0}
if (is.na(P.y)){P.y <- 0}
if (is.na(C.n)){C.n <- 0}
if (is.na(C.y)){C.y <- 0}
p <- fisher.test(matrix(c(P.y,P.n,C.y,C.n), nrow=2))$p.value
if (any(c(P.y, P.n, C.y, C.n)==0)){
  or <- Prop.or(x=c(P.y,P.n), y=c(C.y,C.n), CImethod='Woolf')$estimate
  ci <- paste(round(Prop.or(x=c(P.y,P.n), y=c(C.y,C.n),
                            CImethod='Woolf')$conf.int[1],1),
              round(Prop.or(x=c(P.y,P.n), y=c(C.y,C.n),
                            CImethod='Woolf')$conf.int[2],1), sep='-')
}else{
  or <- fisher.test(matrix(c(P.y,P.n,C.y,C.n), nrow=2))$estimate
  ci <- paste(round(fisher.test(matrix(c(P.y,P.n,C.y,C.n),
                                       nrow=2))$conf.int[1],1),
              round(fisher.test(matrix(c(P.y,P.n,C.y,C.n),
                                       nrow=2))$conf.int[2],1), sep='-')
}
results <- rbind(results,
                 data.frame(Category='GI health in 3 months prior to stool collection',
                            Metadata ="Constipation (< 3 bowel movements per week)",
                            `PD N`=P.n+P.y,
                            `PD summary stats`=paste(P.y,
                                                     " ","(",
                                                     round(P.y/(P.n+P.y)*100, 0),
                                                     "%",")",
                                                     sep=""),
                            `NHC N`=C.n+C.y,
                            `NHC summary stats`=paste(C.y,
                                                      " ","(",
                                                      round(C.y/(C.n+C.y)*100, 0),
```

```
                                                     "%",")",
                                                     sep=""),
                                 `Total N`=P.n+P.y+C.n+C.y,
                                 P=formatC(p, format="e", digits=1),
                                 `OR [95%CI]`=paste(round(or, 1),' [',ci,']',sep=''),
                                 check.names=FALSE))
# diarrhea (once a week or more)
P.y <- table(subset(subject.data, Case_status == "PD")$Diarrhea)['Y']
P.n <- table(subset(subject.data, Case_status == "PD")$Diarrhea)['N']
C.y <- table(subset(subject.data, Case_status == "Control")$Diarrhea)['Y']
C.n <- table(subset(subject.data, Case_status == "Control")$Diarrhea)['N']
if (is.na(P.n)){P.n <- 0}
if (is.na(P.y)){P.y <- 0}
if (is.na(C.n)){C.n <- 0}
if (is.na(C.y)){C.y <- 0}
p <- fisher.test(matrix(c(P.y,P.n,C.y,C.n), nrow=2))$p.value
if (any(c(P.y, P.n, C.y, C.n)==0)){
  or <- Prop.or(x=c(P.y,P.n), y=c(C.y,C.n), CImethod='Woolf')$estimate
  ci <- paste(round(Prop.or(x=c(P.y,P.n), y=c(C.y,C.n),
                    CImethod='Woolf')$conf.int[1],1),
              round(Prop.or(x=c(P.y,P.n), y=c(C.y,C.n),
                    CImethod='Woolf')$conf.int[2],1), sep='-')
}else{
  or <- fisher.test(matrix(c(P.y,P.n,C.y,C.n), nrow=2))$estimate
  ci <- paste(round(fisher.test(matrix(c(P.y,P.n,C.y,C.n),
                               nrow=2))$conf.int[1],1),
              round(fisher.test(matrix(c(P.y,P.n,C.y,C.n),
                               nrow=2))$conf.int[2],1), sep='-')
}
results <- rbind(results, data.frame(Category='',
                                     Metadata ="Diarrhea (once a week or more)",
                                     `PD N`=P.n+P.y,
                                     `PD summary stats`=paste(P.y,
                                                              " ","(",
                                                              round(P.y/(P.n+P.y)*100, 0),
                                                              "%",")",
                                                                sep=""),
                                     `NHC N`=C.n+C.y,
                                     `NHC summary stats`=paste(C.y,
                                                               " ","(",
                                                               round(C.y/(C.n+C.y)*100, 0),
                                                               "%",")",
                                                                sep=""),
                                     `Total N`=P.n+P.y+C.n+C.y,
                                     P=formatC(p, format="e", digits=1),
                                     `OR [95%CI]`=paste(round(or, 1),' [',ci,']',sep=''),
                                     check.names=FALSE))
# colitis
P.y <- table(subset(subject.data, Case_status == "PD")$Colitis)['Y']
P.n <- table(subset(subject.data, Case_status == "PD")$Colitis)['N']
C.y <- table(subset(subject.data, Case_status == "Control")$Colitis)['Y']
C.n <- table(subset(subject.data, Case_status == "Control")$Colitis)['N']
if (is.na(P.n)){P.n <- 0}
if (is.na(P.y)){P.y <- 0}
```

```
if (is.na(C.n)){C.n <- 0}
if (is.na(C.y)){C.y <- 0}
p <- fisher.test(matrix(c(P.y,P.n,C.y,C.n), nrow=2))$p.value
if (any(c(P.y, P.n, C.y, C.n)==0)){
  or <- Prop.or(x=c(P.y,P.n), y=c(C.y,C.n), CImethod='Woolf')$estimate
  ci <- paste(round(Prop.or(x=c(P.y,P.n), y=c(C.y,C.n),
                    CImethod='Woolf')$conf.int[1],1),
              round(Prop.or(x=c(P.y,P.n), y=c(C.y,C.n),
                    CImethod='Woolf')$conf.int[2],1), sep='-')
}else{
  or <- fisher.test(matrix(c(P.y,P.n,C.y,C.n), nrow=2))$estimate
  ci <- paste(round(fisher.test(matrix(c(P.y,P.n,C.y,C.n),
                               nrow=2))$conf.int[1],1),
              round(fisher.test(matrix(c(P.y,P.n,C.y,C.n),
                               nrow=2))$conf.int[2],1), sep='-')
}
results <- rbind(results, data.frame(Category='GI disease',
                                     Metadata ="Colitis",
                                     `PD N`=P.n+P.y,
                                     `PD summary stats`=paste(P.y,
                                                         " ","(",
                                                         round(P.y/(P.n+P.y)*100, 0),
                                                         "%",")",
                                                           sep=""),
                                     `NHC N`=C.n+C.y,
                                     `NHC summary stats`=paste(C.y,
                                                          " ","(",
                                                          round(C.y/(C.n+C.y)*100, 0),
                                                          "%",")",
                                                            sep=""),
                                     `Total N`=P.n+P.y+C.n+C.y,
                                     P=formatC(p, format="e", digits=1),
                                     `OR [95%CI]`=paste(round(or, 1),' [',ci,']',sep=''),
                                     check.names=FALSE))
# IBS
P.y <- table(subset(subject.data, Case_status == "PD")$IBS)['Y']
P.n <- table(subset(subject.data, Case_status == "PD")$IBS)['N']
C.y <- table(subset(subject.data, Case_status == "Control")$IBS)['Y']
C.n <- table(subset(subject.data, Case_status == "Control")$IBS)['N']
if (is.na(P.n)){P.n <- 0}
if (is.na(P.y)){P.y <- 0}
if (is.na(C.n)){C.n <- 0}
if (is.na(C.y)){C.y <- 0}
p <- fisher.test(matrix(c(P.y,P.n,C.y,C.n), nrow=2))$p.value
if (any(c(P.y, P.n, C.y, C.n)==0)){
  or <- Prop.or(x=c(P.y,P.n), y=c(C.y,C.n), CImethod='Woolf')$estimate
  ci <- paste(round(Prop.or(x=c(P.y,P.n), y=c(C.y,C.n),
                    CImethod='Woolf')$conf.int[1],1),
              round(Prop.or(x=c(P.y,P.n), y=c(C.y,C.n),
                    CImethod='Woolf')$conf.int[2],1), sep='-')
}else{
  or <- fisher.test(matrix(c(P.y,P.n,C.y,C.n), nrow=2))$estimate
  ci <- paste(round(fisher.test(matrix(c(P.y,P.n,C.y,C.n),
                               nrow=2))$conf.int[1],1),
```

```
                round(fisher.test(matrix(c(P.y,P.n,C.y,C.n),
                                    nrow=2))$conf.int[2],1), sep='-')
}
results <- rbind(results, data.frame(Category='',
                                     Metadata ="Irritable bowel syndrome",
                                     `PD N`=P.n+P.y,
                                     `PD summary stats`=paste(P.y,
                                                         " ","(",
                                                         round(P.y/(P.n+P.y)*100, 0),
                                                         "%",")",
                                                           sep=""),
                                     `NHC N`=C.n+C.y,
                                     `NHC summary stats`=paste(C.y,
                                                          " ","(",
                                                          round(C.y/(C.n+C.y)*100, 0),
                                                          "%",")",
                                                            sep=""),
                                     `Total N`=P.n+P.y+C.n+C.y,
                                     P=formatC(p, format="e", digits=1),
                                     `OR [95%CI]`=paste(round(or, 1),' [',ci,']',sep=''),
                                     check.names=FALSE))
# Crohn's disease
P.y <- table(subset(subject.data, Case_status == "PD")$Crohns_disease)['Y']
P.n <- table(subset(subject.data, Case_status == "PD")$Crohns_disease)['N']
C.y <- table(subset(subject.data, Case_status == "Control")$Crohns_disease)['Y']
C.n <- table(subset(subject.data, Case_status == "Control")$Crohns_disease)['N']
if (is.na(P.n)){P.n <- 0}
if (is.na(P.y)){P.y <- 0}
if (is.na(C.n)){C.n <- 0}
if (is.na(C.y)){C.y <- 0}
p <- fisher.test(matrix(c(P.y,P.n,C.y,C.n), nrow=2))$p.value
if (any(c(P.y, P.n, C.y, C.n)==0)){
  or <- Prop.or(x=c(P.y,P.n), y=c(C.y,C.n), CImethod='Woolf')$estimate
  ci <- paste(round(Prop.or(x=c(P.y,P.n), y=c(C.y,C.n),
                            CImethod='Woolf')$conf.int[1],1),
              round(Prop.or(x=c(P.y,P.n), y=c(C.y,C.n),
                            CImethod='Woolf')$conf.int[2],1), sep='-')
}else{
  or <- fisher.test(matrix(c(P.y,P.n,C.y,C.n), nrow=2))$estimate
  ci <- paste(round(fisher.test(matrix(c(P.y,P.n,C.y,C.n),
                                    nrow=2))$conf.int[1],1),
              round(fisher.test(matrix(c(P.y,P.n,C.y,C.n),
                                    nrow=2))$conf.int[2],1), sep='-')
}
results <- rbind(results, data.frame(Category='',
                                     Metadata ="Crohn's disease",
                                     `PD N`=P.n+P.y,
                                     `PD summary stats`=paste(P.y,
                                                         " ","(",
                                                         round(P.y/(P.n+P.y)*100, 1),
                                                         "%",")",
                                                         sep=""),
                                     `NHC N`=C.n+C.y,
                                     `NHC summary stats`=paste(C.y,
```

```
                                                               " ","(",
                                                               round(C.y/(C.n+C.y)*100, 1),
                                                               "%",")",
                                                               sep=""),
                                     `Total N`=P.n+P.y+C.n+C.y,
                                     P=formatC(p, format="e", digits=1),
                                     `OR [95%CI]`=paste(round(or, 1),' [',ci,']',sep=''),
                                     check.names=FALSE))
# IBD
P.y <- table(subset(subject.data, Case_status == "PD")$IBD)['Y']
P.n <- table(subset(subject.data, Case_status == "PD")$IBD)['N']
C.y <- table(subset(subject.data, Case_status == "Control")$IBD)['Y']
C.n <- table(subset(subject.data, Case_status == "Control")$IBD)['N']
if (is.na(P.n)){P.n <- 0}
if (is.na(P.y)){P.y <- 0}
if (is.na(C.n)){C.n <- 0}
if (is.na(C.y)){C.y <- 0}
p <- fisher.test(matrix(c(P.y,P.n,C.y,C.n), nrow=2))$p.value
if (any(c(P.y, P.n, C.y, C.n)==0)){
  or <- Prop.or(x=c(P.y,P.n), y=c(C.y,C.n), CImethod='Woolf')$estimate
  ci <- paste(round(Prop.or(x=c(P.y,P.n), y=c(C.y,C.n),
                            CImethod='Woolf')$conf.int[1],1),
              round(Prop.or(x=c(P.y,P.n), y=c(C.y,C.n),
                            CImethod='Woolf')$conf.int[2],1), sep='-')
}else{
  or <- fisher.test(matrix(c(P.y,P.n,C.y,C.n), nrow=2))$estimate
  ci <- paste(round(fisher.test(matrix(c(P.y,P.n,C.y,C.n),
                                       nrow=2))$conf.int[1],1),
              round(fisher.test(matrix(c(P.y,P.n,C.y,C.n),
                                       nrow=2))$conf.int[2],1), sep='-')
}
results <- rbind(results, data.frame(Category='',
                                     Metadata ="Inflammatory bowel disease",
                                     `PD N`=P.n+P.y,
                                     `PD summary stats`=paste(P.y,
                                                              " ","(",
                                                              round(P.y/(P.n+P.y)*100, 0),
                                                              "%",")",
                                                                sep=""),
                                     `NHC N`=C.n+C.y,
                                     `NHC summary stats`=paste(C.y,
                                                               " ","(",
                                                               round(C.y/(C.n+C.y)*100, 0),
                                                               "%",")",
                                                                 sep=""),
                                     `Total N`=P.n+P.y+C.n+C.y,
                                     P=formatC(p, format="e", digits=1),
                                     `OR [95%CI]`=paste(round(or, 1),' [',ci,']',sep=''),
                                     check.names=FALSE))
# ulcers in past 3 months
P.y <- table(subset(subject.data, Case_status == "PD")$Ulcer_past_3_months)['Y']
P.n <- table(subset(subject.data, Case_status == "PD")$Ulcer_past_3_months)['N']
C.y <- table(subset(subject.data, Case_status == "Control")$Ulcer_past_3_months)['Y']
C.n <- table(subset(subject.data, Case_status == "Control")$Ulcer_past_3_months)['N']
```

```
if (is.na(P.n)){P.n <- 0}
if (is.na(P.y)){P.y <- 0}
if (is.na(C.n)){C.n <- 0}
if (is.na(C.y)){C.y <- 0}
p <- fisher.test(matrix(c(P.y,P.n,C.y,C.n), nrow=2))$p.value
if (any(c(P.y, P.n, C.y, C.n)==0)){
  or <- Prop.or(x=c(P.y,P.n), y=c(C.y,C.n), CImethod='Woolf')$estimate
  ci <- paste(round(Prop.or(x=c(P.y,P.n), y=c(C.y,C.n),
                            CImethod='Woolf')$conf.int[1],1),
              round(Prop.or(x=c(P.y,P.n), y=c(C.y,C.n),
                            CImethod='Woolf')$conf.int[2],1), sep='-')
}else{
  or <- fisher.test(matrix(c(P.y,P.n,C.y,C.n), nrow=2))$estimate
  ci <- paste(round(fisher.test(matrix(c(P.y,P.n,C.y,C.n),
                                       nrow=2))$conf.int[1],1),
              round(fisher.test(matrix(c(P.y,P.n,C.y,C.n),
                                       nrow=2))$conf.int[2],1), sep='-')
}
results <- rbind(results, data.frame(Category='',
                                     Metadata ="Ulcers in the past 3 months",
                                     `PD N`=P.n+P.y,
                                     `PD summary stats`=paste(P.y,
                                                              " ","(",
                                                              round(P.y/(P.n+P.y)*100, 0),
                                                              "%",")",
                                                                sep=""),
                                     `NHC N`=C.n+C.y,
                                     `NHC summary stats`=paste(C.y,
                                                               " ","(",
                                                               round(C.y/(C.n+C.y)*100, 1),
                                                               "%",")",
                                                                sep=""),
                                     `Total N`=P.n+P.y+C.n+C.y,
                                     P=formatC(p, format="e", digits=1),
                                     `OR [95%CI]`=paste(round(or, 1),' [',ci,']',sep=''),
                                     check.names=FALSE))
# SIBO
P.y <- table(subset(subject.data, Case_status == "PD")$SIBO)['Y']
P.n <- table(subset(subject.data, Case_status == "PD")$SIBO)['N']
C.y <- table(subset(subject.data, Case_status == "Control")$SIBO)['Y']
C.n <- table(subset(subject.data, Case_status == "Control")$SIBO)['N']
if (is.na(P.n)){P.n <- 0}
if (is.na(P.y)){P.y <- 0}
if (is.na(C.n)){C.n <- 0}
if (is.na(C.y)){C.y <- 0}
p <- fisher.test(matrix(c(P.y,P.n,C.y,C.n), nrow=2))$p.value
if (any(c(P.y, P.n, C.y, C.n)==0)){
  or <- Prop.or(x=c(P.y,P.n), y=c(C.y,C.n), CImethod='Woolf')$estimate
  ci <- paste(round(Prop.or(x=c(P.y,P.n), y=c(C.y,C.n),
                            CImethod='Woolf')$conf.int[1],1),
              round(Prop.or(x=c(P.y,P.n), y=c(C.y,C.n),
                            CImethod='Woolf')$conf.int[2],1), sep='-')
}else{
  or <- fisher.test(matrix(c(P.y,P.n,C.y,C.n), nrow=2))$estimate
```

```
  ci <- paste(round(fisher.test(matrix(c(P.y,P.n,C.y,C.n),
                                       nrow=2))$conf.int[1],1),
              round(fisher.test(matrix(c(P.y,P.n,C.y,C.n),
                                       nrow=2))$conf.int[2],1), sep='-')
}
results <- rbind(results, data.frame(Category='',
                                     Metadata ="Small instestinal bacterial overgrowth",
                                     `PD N`=P.n+P.y,
                                     `PD summary stats`=paste(P.y,
                                                              " ","(",
                                                              round(P.y/(P.n+P.y)*100, 1),
                                                              "%",")",
                                                                sep=""),
                                     `NHC N`=C.n+C.y,
                                     `NHC summary stats`=paste(C.y,
                                                               " ","(",
                                                               round(C.y/(C.n+C.y)*100, 0),
                                                               "%",")",
                                                                 sep=""),
                                     `Total N`=P.n+P.y+C.n+C.y,
                                     P=formatC(p, format="e", digits=1),
                                     `OR [95%CI]`=paste(round(or, 1),' [',ci,']',sep=''),
                                     check.names=FALSE))
# Celiac disease
P.y <- table(subset(subject.data, Case_status == "PD")$Celiac_disease)['Y']
P.n <- table(subset(subject.data, Case_status == "PD")$Celiac_disease)['N']
C.y <- table(subset(subject.data, Case_status == "Control")$Celiac_disease)['Y']
C.n <- table(subset(subject.data, Case_status == "Control")$Celiac_disease)['N']
if (is.na(P.n)){P.n <- 0}
if (is.na(P.y)){P.y <- 0}
if (is.na(C.n)){C.n <- 0}
if (is.na(C.y)){C.y <- 0}
p <- fisher.test(matrix(c(P.y,P.n,C.y,C.n), nrow=2))$p.value
if (any(c(P.y, P.n, C.y, C.n)==0)){
  or <- Prop.or(x=c(P.y,P.n), y=c(C.y,C.n), CImethod='Woolf')$estimate
  ci <- paste(round(Prop.or(x=c(P.y,P.n), y=c(C.y,C.n),
                            CImethod='Woolf')$conf.int[1],1),
              round(Prop.or(x=c(P.y,P.n), y=c(C.y,C.n),
                            CImethod='Woolf')$conf.int[2],1), sep='-')
}else{
  or <- fisher.test(matrix(c(P.y,P.n,C.y,C.n), nrow=2))$estimate
  ci <- paste(round(fisher.test(matrix(c(P.y,P.n,C.y,C.n),
                                       nrow=2))$conf.int[1],1),
              round(fisher.test(matrix(c(P.y,P.n,C.y,C.n),
                                       nrow=2))$conf.int[2],1), sep='-')
}
results <- rbind(results, data.frame(Category='',
                                     Metadata ="Celiac disease",
                                     `PD N`=P.n+P.y,
                                     `PD summary stats`=paste(P.y,
                                                              " ","(",
                                                              round(P.y/(P.n+P.y)*100, 1),
                                                              "%",")",
                                                                sep=""),
```

```r
                                        `NHC N`=C.n+C.y,
                                        `NHC summary stats`=paste(C.y,
                                                            " ","(",
                                                            round(C.y/(C.n+C.y)*100, 0),
                                                            "%",")",
                                                              sep=""),
                                        `Total N`=P.n+P.y+C.n+C.y,
                                        P=formatC(p, format="e", digits=1),
                                        `OR [95%CI]`=paste(round(or, 1),' [',ci,']',sep=''),
                                        check.names=FALSE))
# GI cancer in past 3 months
P.y <- table(subset(subject.data, Case_status == "PD")$GI_cancer_past_3_months)['Y']
P.n <- table(subset(subject.data, Case_status == "PD")$GI_cancer_past_3_months)['N']
C.y <- table(subset(subject.data, Case_status == "Control")$GI_cancer_past_3_months)['Y']
C.n <- table(subset(subject.data, Case_status == "Control")$GI_cancer_past_3_months)['N']
if (is.na(P.n)){P.n <- 0}
if (is.na(P.y)){P.y <- 0}
if (is.na(C.n)){C.n <- 0}
if (is.na(C.y)){C.y <- 0}
p <- fisher.test(matrix(c(P.y,P.n,C.y,C.n), nrow=2))$p.value
if (any(c(P.y, P.n, C.y, C.n)==0)){
  or <- Prop.or(x=c(P.y,P.n), y=c(C.y,C.n), CImethod='Woolf')$estimate
  ci <- paste(round(Prop.or(x=c(P.y,P.n), y=c(C.y,C.n),
                            CImethod='Woolf')$conf.int[1],1),
              round(Prop.or(x=c(P.y,P.n), y=c(C.y,C.n),
                            CImethod='Woolf')$conf.int[2],1), sep='-')
}else{
  or <- fisher.test(matrix(c(P.y,P.n,C.y,C.n), nrow=2))$estimate
  ci <- paste(round(fisher.test(matrix(c(P.y,P.n,C.y,C.n),
                                       nrow=2))$conf.int[1],1),
              round(fisher.test(matrix(c(P.y,P.n,C.y,C.n),
                                       nrow=2))$conf.int[2],1), sep='-')
}
results <- rbind(results, data.frame(Category='',
                                        Metadata ="GI cancer in the last 3 months",
                                        `PD N`=P.n+P.y,
                                        `PD summary stats`=paste(P.y,
                                                           " ","(",
                                                           round(P.y/(P.n+P.y)*100, 1),
                                                           "%",")",
                                                             sep=""),
                                        `NHC N`=C.n+C.y,
                                        `NHC summary stats`=paste(C.y,
                                                            " ","(",
                                                            round(C.y/(C.n+C.y)*100, 0),
                                                            "%",")",
                                                              sep=""),
                                        `Total N`=P.n+P.y+C.n+C.y,
                                        P=formatC(p, format="e", digits=1),
                                        `OR [95%CI]`=paste(round(or, 1),' [',ci,']',sep=''),
                                        check.names=FALSE))
# intestinal disease
P.y <- table(subset(subject.data, Case_status == "PD")$Intestinal_disease)['Y']
P.n <- table(subset(subject.data, Case_status == "PD")$Intestinal_disease)['N']
```

```
C.y <- table(subset(subject.data, Case_status == "Control")$Intestinal_disease)['Y']
C.n <- table(subset(subject.data, Case_status == "Control")$Intestinal_disease)['N']
if (is.na(P.n)){P.n <- 0}
if (is.na(P.y)){P.y <- 0}
if (is.na(C.n)){C.n <- 0}
if (is.na(C.y)){C.y <- 0}
p <- fisher.test(matrix(c(P.y,P.n,C.y,C.n), nrow=2))$p.value
if (any(c(P.y, P.n, C.y, C.n)==0)){
  or <- Prop.or(x=c(P.y,P.n), y=c(C.y,C.n), CImethod='Woolf')$estimate
  ci <- paste(round(Prop.or(x=c(P.y,P.n), y=c(C.y,C.n),
                    CImethod='Woolf')$conf.int[1],1),
              round(Prop.or(x=c(P.y,P.n), y=c(C.y,C.n),
                    CImethod='Woolf')$conf.int[2],1), sep='-')
}else{
  or <- fisher.test(matrix(c(P.y,P.n,C.y,C.n), nrow=2))$estimate
  ci <- paste(round(fisher.test(matrix(c(P.y,P.n,C.y,C.n),
                               nrow=2))$conf.int[1],1),
              round(fisher.test(matrix(c(P.y,P.n,C.y,C.n),
                               nrow=2))$conf.int[2],1), sep='-')
}
results <- rbind(results, data.frame(Category='',
                                     Metadata ="GI disease (yes to any of the eight items)",
                                     `PD N`=P.n+P.y,
                                     `PD summary stats`=paste(P.y,
                                                              " ","(",
                                                              round(P.y/(P.n+P.y)*100, 0),
                                                              "%",")",
                                                               sep=""),
                                     `NHC N`=C.n+C.y,
                                     `NHC summary stats`=paste(C.y,
                                                               " ","(",
                                                               round(C.y/(C.n+C.y)*100, 0),
                                                               "%",")",
                                                                sep=""),
                                     `Total N`=P.n+P.y+C.n+C.y,
                                     P=formatC(p, format="e", digits=1),
                                     `OR [95%CI]`=paste(round(or, 1),' [',ci,']',sep=''),
                                     check.names=FALSE))
```

```
# indigestion drugs
P.y <- table(subset(subject.data, Case_status == "PD")$Indigestion_drugs)['Y']
P.n <- table(subset(subject.data, Case_status == "PD")$Indigestion_drugs)['N']
C.y <- table(subset(subject.data, Case_status == "Control")$Indigestion_drugs)['Y']
C.n <- table(subset(subject.data, Case_status == "Control")$Indigestion_drugs)['N']
if (is.na(P.n)){P.n <- 0}
if (is.na(P.y)){P.y <- 0}
if (is.na(C.n)){C.n <- 0}
if (is.na(C.y)){C.y <- 0}
p <- fisher.test(matrix(c(P.y,P.n,C.y,C.n), nrow=2))$p.value
if (any(c(P.y, P.n, C.y, C.n)==0)){
  or <- Prop.or(x=c(P.y,P.n), y=c(C.y,C.n), CImethod='Woolf')$estimate
  ci <- paste(round(Prop.or(x=c(P.y,P.n), y=c(C.y,C.n),
                    CImethod='Woolf')$conf.int[1],1),
              round(Prop.or(x=c(P.y,P.n), y=c(C.y,C.n),
```

```
                            CImethod='Woolf')$conf.int[2],1), sep='-')
}else{
  or <- fisher.test(matrix(c(P.y,P.n,C.y,C.n), nrow=2))$estimate
  ci <- paste(round(fisher.test(matrix(c(P.y,P.n,C.y,C.n),
                                       nrow=2))$conf.int[1],1),
              round(fisher.test(matrix(c(P.y,P.n,C.y,C.n),
                                       nrow=2))$conf.int[2],1), sep='-')
}
results <- rbind(results,
 data.frame(Category='Medications (currently taking at time of stool collection, unless noted)',
            Metadata ="Indigestion drugs",
            `PD N`=P.n+P.y,
            `PD summary stats`=paste(P.y,
                                     " ","(",
                                     round(P.y/(P.n+P.y)*100, 0),
                                     "%",")",
                                        sep=""),
            `NHC N`=C.n+C.y,
            `NHC summary stats`=paste(C.y,
                                      " ","(",
                                      round(C.y/(C.n+C.y)*100, 0),
                                      "%",")",
                                       sep=""),
            `Total N`=P.n+P.y+C.n+C.y,
            P=formatC(p, format="e", digits=1),
            `OR [95%CI]`=paste(round(or, 1),' [',ci,']',sep=''),
            check.names=FALSE))
# antibiotics
P.y <- table(subset(subject.data, Case_status == "PD")$Antibiotics_current)['Y']
P.n <- table(subset(subject.data, Case_status == "PD")$Antibiotics_current)['N']
C.y <- table(subset(subject.data, Case_status == "Control")$Antibiotics_current)['Y']
C.n <- table(subset(subject.data, Case_status == "Control")$Antibiotics_current)['N']
if (is.na(P.n)){P.n <- 0}
if (is.na(P.y)){P.y <- 0}
if (is.na(C.n)){C.n <- 0}
if (is.na(C.y)){C.y <- 0}
p <- fisher.test(matrix(c(P.y,P.n,C.y,C.n), nrow=2))$p.value
if (any(c(P.y, P.n, C.y, C.n)==0)){
  or <- Prop.or(x=c(P.y,P.n), y=c(C.y,C.n), CImethod='Woolf')$estimate
  ci <- paste(round(Prop.or(x=c(P.y,P.n), y=c(C.y,C.n),
                            CImethod='Woolf')$conf.int[1],1),
              round(Prop.or(x=c(P.y,P.n), y=c(C.y,C.n),
                            CImethod='Woolf')$conf.int[2],1), sep='-')
}else{
  or <- fisher.test(matrix(c(P.y,P.n,C.y,C.n), nrow=2))$estimate
  ci <- paste(round(fisher.test(matrix(c(P.y,P.n,C.y,C.n),
                                       nrow=2))$conf.int[1],1),
              round(fisher.test(matrix(c(P.y,P.n,C.y,C.n),
                                       nrow=2))$conf.int[2],1), sep='-')
}
results <- rbind(results, data.frame(Category='',
                                     Metadata ="Antibiotics",
                                     `PD N`=P.n+P.y,
                                     `PD summary stats`=paste(P.y,
```

```
                                                              " ","(",
                                                              round(P.y/(P.n+P.y)*100, 0),
                                                              "%",")",
                                                                sep=""),
                                     `NHC N`=C.n+C.y,
                                     `NHC summary stats`=paste(C.y,
                                                               " ","(",
                                                               round(C.y/(C.n+C.y)*100, 0),
                                                               "%",")",
                                                                 sep=""),
                                     `Total N`=P.n+P.y+C.n+C.y,
                                     P=formatC(p, format="e", digits=1),
                                     `OR [95%CI]`=paste(round(or, 1),' [',ci,']',sep=''),
                                     check.names=FALSE))
# antibiotics in past 3 months
P.y <- table(subset(subject.data, Case_status == "PD")$Antibiotics_past_3_months)['Y']
P.n <- table(subset(subject.data, Case_status == "PD")$Antibiotics_past_3_months)['N']
C.y <- table(subset(subject.data, Case_status == "Control")$Antibiotics_past_3_months)['Y']
C.n <- table(subset(subject.data, Case_status == "Control")$Antibiotics_past_3_months)['N']
if (is.na(P.n)){P.n <- 0}
if (is.na(P.y)){P.y <- 0}
if (is.na(C.n)){C.n <- 0}
if (is.na(C.y)){C.y <- 0}
p <- fisher.test(matrix(c(P.y,P.n,C.y,C.n), nrow=2))$p.value
if (any(c(P.y, P.n, C.y, C.n)==0)){
  or <- Prop.or(x=c(P.y,P.n), y=c(C.y,C.n), CImethod='Woolf')$estimate
  ci <- paste(round(Prop.or(x=c(P.y,P.n), y=c(C.y,C.n),
                            CImethod='Woolf')$conf.int[1],1),
              round(Prop.or(x=c(P.y,P.n), y=c(C.y,C.n),
                            CImethod='Woolf')$conf.int[2],1), sep='-')
}else{
  or <- fisher.test(matrix(c(P.y,P.n,C.y,C.n), nrow=2))$estimate
  ci <- paste(round(fisher.test(matrix(c(P.y,P.n,C.y,C.n),
                                       nrow=2))$conf.int[1],1),
              round(fisher.test(matrix(c(P.y,P.n,C.y,C.n),
                                       nrow=2))$conf.int[2],1), sep='-')
}
results <- rbind(results, data.frame(Category='',
                                     Metadata ="Antibiotics in past 3 months",
                                     `PD N`=P.n+P.y,
                                     `PD summary stats`=paste(P.y,
                                                              " ","(",
                                                              round(P.y/(P.n+P.y)*100, 0),
                                                              "%",")",
                                                                sep=""),
                                     `NHC N`=C.n+C.y,
                                     `NHC summary stats`=paste(C.y,
                                                               " ","(",
                                                               round(C.y/(C.n+C.y)*100, 0),
                                                               "%",")",
                                                                 sep=""),
                                     `Total N`=P.n+P.y+C.n+C.y,
                                     P=formatC(p, format="e", digits=1),
                                     `OR [95%CI]`=paste(round(or, 1),' [',ci,']',sep=''),
```

```
                                     check.names=FALSE))
# laxatives
P.y <- table(subset(subject.data, Case_status == "PD")$Laxatives)['Y']
P.n <- table(subset(subject.data, Case_status == "PD")$Laxatives)['N']
C.y <- table(subset(subject.data, Case_status == "Control")$Laxatives)['Y']
C.n <- table(subset(subject.data, Case_status == "Control")$Laxatives)['N']
if (is.na(P.n)){P.n <- 0}
if (is.na(P.y)){P.y <- 0}
if (is.na(C.n)){C.n <- 0}
if (is.na(C.y)){C.y <- 0}
p <- fisher.test(matrix(c(P.y,P.n,C.y,C.n), nrow=2))$p.value
if (any(c(P.y, P.n, C.y, C.n)==0)){
  or <- Prop.or(x=c(P.y,P.n), y=c(C.y,C.n), CImethod='Woolf')$estimate
  ci <- paste(round(Prop.or(x=c(P.y,P.n), y=c(C.y,C.n),
                            CImethod='Woolf')$conf.int[1],1),
              round(Prop.or(x=c(P.y,P.n), y=c(C.y,C.n),
                            CImethod='Woolf')$conf.int[2],1), sep='-')
}else{
  or <- fisher.test(matrix(c(P.y,P.n,C.y,C.n), nrow=2))$estimate
  ci <- paste(round(fisher.test(matrix(c(P.y,P.n,C.y,C.n),
                                       nrow=2))$conf.int[1],1),
              round(fisher.test(matrix(c(P.y,P.n,C.y,C.n),
                                       nrow=2))$conf.int[2],1), sep='-')
}
results <- rbind(results, data.frame(Category='',
                                     Metadata ="Laxatives",
                                     `PD N`=P.n+P.y,
                                     `PD summary stats`=paste(P.y,
                                                              " ","(",
                                                              round(P.y/(P.n+P.y)*100, 0),
                                                              "%",")",
                                                                sep=""),
                                     `NHC N`=C.n+C.y,
                                     `NHC summary stats`=paste(C.y,
                                                               " ","(",
                                                               round(C.y/(C.n+C.y)*100, 0),
                                                               "%",")",
                                                                 sep=""),
                                     `Total N`=P.n+P.y+C.n+C.y,
                                     P=formatC(p, format="e", digits=1),
                                     `OR [95%CI]`=paste(round(or, 1),' [',ci,']',sep=''),
                                     check.names=FALSE))
# anti-inflammatory drugs
P.y <- table(subset(subject.data, Case_status == "PD")$Anti_inflammatory_drugs)['Y']
P.n <- table(subset(subject.data, Case_status == "PD")$Anti_inflammatory_drugs)['N']
C.y <- table(subset(subject.data, Case_status == "Control")$Anti_inflammatory_drugs)['Y']
C.n <- table(subset(subject.data, Case_status == "Control")$Anti_inflammatory_drugs)['N']
if (is.na(P.n)){P.n <- 0}
if (is.na(P.y)){P.y <- 0}
if (is.na(C.n)){C.n <- 0}
if (is.na(C.y)){C.y <- 0}
p <- fisher.test(matrix(c(P.y,P.n,C.y,C.n), nrow=2))$p.value
if (any(c(P.y, P.n, C.y, C.n)==0)){
  or <- Prop.or(x=c(P.y,P.n), y=c(C.y,C.n), CImethod='Woolf')$estimate
```

```
  ci <- paste(round(Prop.or(x=c(P.y,P.n), y=c(C.y,C.n),
                           CImethod='Woolf')$conf.int[1],1),
              round(Prop.or(x=c(P.y,P.n), y=c(C.y,C.n),
                           CImethod='Woolf')$conf.int[2],1), sep='-')
}else{
  or <- fisher.test(matrix(c(P.y,P.n,C.y,C.n), nrow=2))$estimate
  ci <- paste(round(fisher.test(matrix(c(P.y,P.n,C.y,C.n),
                                      nrow=2))$conf.int[1],1),
              round(fisher.test(matrix(c(P.y,P.n,C.y,C.n),
                                      nrow=2))$conf.int[2],1), sep='-')
}
results <- rbind(results, data.frame(Category='',
                                     Metadata ="Anti-inflammatory drugs",
                                     `PD N`=P.n+P.y,
                                     `PD summary stats`=paste(P.y,
                                                         " ","(",
                                                         round(P.y/(P.n+P.y)*100, 0),
                                                         "%",")",
                                                           sep=""),
                                     `NHC N`=C.n+C.y,
                                     `NHC summary stats`=paste(C.y,
                                                          " ","(",
                                                          round(C.y/(C.n+C.y)*100, 0),
                                                          "%",")",
                                                            sep=""),
                                     `Total N`=P.n+P.y+C.n+C.y,
                                     P=formatC(p, format="e", digits=1),
                                     `OR [95%CI]`=paste(round(or, 1),' [',ci,']',sep=''),
                                     check.names=FALSE))
# probiotics
P.y <- table(subset(subject.data, Case_status == "PD")$Probiotic)['Y']
P.n <- table(subset(subject.data, Case_status == "PD")$Probiotic)['N']
C.y <- table(subset(subject.data, Case_status == "Control")$Probiotic)['Y']
C.n <- table(subset(subject.data, Case_status == "Control")$Probiotic)['N']
if (is.na(P.n)){P.n <- 0}
if (is.na(P.y)){P.y <- 0}
if (is.na(C.n)){C.n <- 0}
if (is.na(C.y)){C.y <- 0}
p <- fisher.test(matrix(c(P.y,P.n,C.y,C.n), nrow=2))$p.value
if (any(c(P.y, P.n, C.y, C.n)==0)){
  or <- Prop.or(x=c(P.y,P.n), y=c(C.y,C.n), CImethod='Woolf')$estimate
  ci <- paste(round(Prop.or(x=c(P.y,P.n), y=c(C.y,C.n),
                           CImethod='Woolf')$conf.int[1],1),
              round(Prop.or(x=c(P.y,P.n), y=c(C.y,C.n),
                           CImethod='Woolf')$conf.int[2],1), sep='-')
}else{
  or <- fisher.test(matrix(c(P.y,P.n,C.y,C.n), nrow=2))$estimate
  ci <- paste(round(fisher.test(matrix(c(P.y,P.n,C.y,C.n),
                                      nrow=2))$conf.int[1],1),
              round(fisher.test(matrix(c(P.y,P.n,C.y,C.n),
                                      nrow=2))$conf.int[2],1), sep='-')
}
results <- rbind(results, data.frame(Category='',
                                     Metadata ="Probiotics",
```

```
                                    `PD N`=P.n+P.y,
                                    `PD summary stats`=paste(P.y,
                                                        " ","(",
                                                        round(P.y/(P.n+P.y)*100, 0),
                                                        "%",")",
                                                          sep=""),
                                    `NHC N`=C.n+C.y,
                                    `NHC summary stats`=paste(C.y,
                                                         " ","(",
                                                         round(C.y/(C.n+C.y)*100, 0),
                                                         "%",")",
                                                           sep=""),
                                    `Total N`=P.n+P.y+C.n+C.y,
                                    P=formatC(p, format="e", digits=1),
                                    `OR [95%CI]`=paste(round(or, 1),' [',ci,']',sep=''),
                                    check.names=FALSE))
# radiation or chemotherapy
P.y <- table(subset(subject.data, Case_status == "PD")$Radiation_Chemo)['Y']
P.n <- table(subset(subject.data, Case_status == "PD")$Radiation_Chemo)['N']
C.y <- table(subset(subject.data, Case_status == "Control")$Radiation_Chemo)['Y']
C.n <- table(subset(subject.data, Case_status == "Control")$Radiation_Chemo)['N']
if (is.na(P.n)){P.n <- 0}
if (is.na(P.y)){P.y <- 0}
if (is.na(C.n)){C.n <- 0}
if (is.na(C.y)){C.y <- 0}
p <- fisher.test(matrix(c(P.y,P.n,C.y,C.n), nrow=2))$p.value
if (any(c(P.y, P.n, C.y, C.n)==0)){
  or <- Prop.or(x=c(P.y,P.n), y=c(C.y,C.n), CImethod='Woolf')$estimate
  ci <- paste(round(Prop.or(x=c(P.y,P.n), y=c(C.y,C.n),
                            CImethod='Woolf')$conf.int[1],1),
              round(Prop.or(x=c(P.y,P.n), y=c(C.y,C.n),
                            CImethod='Woolf')$conf.int[2],1), sep='-')
}else{
  or <- fisher.test(matrix(c(P.y,P.n,C.y,C.n), nrow=2))$estimate
  ci <- paste(round(fisher.test(matrix(c(P.y,P.n,C.y,C.n),
                                       nrow=2))$conf.int[1],1),
              round(fisher.test(matrix(c(P.y,P.n,C.y,C.n),
                                       nrow=2))$conf.int[2],1), sep='-')
}
results <- rbind(results, data.frame(Category='',
                                    Metadata ="Radiation or chemotherapy",
                                    `PD N`=P.n+P.y,
                                    `PD summary stats`=paste(P.y,
                                                        " ","(",
                                                        round(P.y/(P.n+P.y)*100, 0),
                                                        "%",")",
                                                          sep=""),
                                    `NHC N`=C.n+C.y,
                                    `NHC summary stats`=paste(C.y,
                                                         " ","(",
                                                         round(C.y/(C.n+C.y)*100, 0),
                                                         "%",")",
                                                           sep=""),
                                    `Total N`=P.n+P.y+C.n+C.y,
```

```
                                     P=formatC(p, format="e", digits=1),
                                     `OR [95%CI]`=paste(round(or, 1),' [',ci,']',sep=''),
                                     check.names=FALSE))
# blood thinners
P.y <- table(subset(subject.data, Case_status == "PD")$Blood_thinners)['Y']
P.n <- table(subset(subject.data, Case_status == "PD")$Blood_thinners)['N']
C.y <- table(subset(subject.data, Case_status == "Control")$Blood_thinners)['Y']
C.n <- table(subset(subject.data, Case_status == "Control")$Blood_thinners)['N']
if (is.na(P.n)){P.n <- 0}
if (is.na(P.y)){P.y <- 0}
if (is.na(C.n)){C.n <- 0}
if (is.na(C.y)){C.y <- 0}
p <- fisher.test(matrix(c(P.y,P.n,C.y,C.n), nrow=2))$p.value
if (any(c(P.y, P.n, C.y, C.n)==0)){
  or <- Prop.or(x=c(P.y,P.n), y=c(C.y,C.n), CImethod='Woolf')$estimate
  ci <- paste(round(Prop.or(x=c(P.y,P.n), y=c(C.y,C.n),
                            CImethod='Woolf')$conf.int[1],1),
              round(Prop.or(x=c(P.y,P.n), y=c(C.y,C.n),
                            CImethod='Woolf')$conf.int[2],1), sep='-')
}else{
  or <- fisher.test(matrix(c(P.y,P.n,C.y,C.n), nrow=2))$estimate
  ci <- paste(round(fisher.test(matrix(c(P.y,P.n,C.y,C.n),
                                       nrow=2))$conf.int[1],1),
              round(fisher.test(matrix(c(P.y,P.n,C.y,C.n),
                                       nrow=2))$conf.int[2],1), sep='-')
}
results <- rbind(results, data.frame(Category='',
                                     Metadata ="Blood thinners",
                                     `PD N`=P.n+P.y,
                                     `PD summary stats`=paste(P.y,
                                                              " ","(",
                                                              round(P.y/(P.n+P.y)*100, 0),
                                                              "%",")",
                                                                sep=""),
                                     `NHC N`=C.n+C.y,
                                     `NHC summary stats`=paste(C.y,
                                                               " ","(",
                                                               round(C.y/(C.n+C.y)*100, 0),
                                                               "%",")",
                                                                 sep=""),
                                     `Total N`=P.n+P.y+C.n+C.y,
                                     P=formatC(p, format="e", digits=1),
                                     `OR [95%CI]`=paste(round(or, 1),' [',ci,']',sep=''),
                                     check.names=FALSE))
# cholesterol medication
P.y <- table(subset(subject.data, Case_status == "PD")$Cholesterol_med)['Y']
P.n <- table(subset(subject.data, Case_status == "PD")$Cholesterol_med)['N']
C.y <- table(subset(subject.data, Case_status == "Control")$Cholesterol_med)['Y']
C.n <- table(subset(subject.data, Case_status == "Control")$Cholesterol_med)['N']
if (is.na(P.n)){P.n <- 0}
if (is.na(P.y)){P.y <- 0}
if (is.na(C.n)){C.n <- 0}
if (is.na(C.y)){C.y <- 0}
p <- fisher.test(matrix(c(P.y,P.n,C.y,C.n), nrow=2))$p.value
```

```
if (any(c(P.y, P.n, C.y, C.n)==0)){
  or <- Prop.or(x=c(P.y,P.n), y=c(C.y,C.n), CImethod='Woolf')$estimate
  ci <- paste(round(Prop.or(x=c(P.y,P.n), y=c(C.y,C.n),
                            CImethod='Woolf')$conf.int[1],1),
              round(Prop.or(x=c(P.y,P.n), y=c(C.y,C.n),
                            CImethod='Woolf')$conf.int[2],1), sep='-')
}else{
  or <- fisher.test(matrix(c(P.y,P.n,C.y,C.n), nrow=2))$estimate
  ci <- paste(round(fisher.test(matrix(c(P.y,P.n,C.y,C.n),
                                       nrow=2))$conf.int[1],1),
              round(fisher.test(matrix(c(P.y,P.n,C.y,C.n),
                                       nrow=2))$conf.int[2],1), sep='-')
}
results <- rbind(results, data.frame(Category='',
                                     Metadata ="Cholesterol medication",
                                     `PD N`=P.n+P.y,
                                     `PD summary stats`=paste(P.y,
                                                              " ","(",
                                                              round(P.y/(P.n+P.y)*100, 0),
                                                              "%",")",
                                                                sep=""),
                                     `NHC N`=C.n+C.y,
                                     `NHC summary stats`=paste(C.y,
                                                               " ","(",
                                                               round(C.y/(C.n+C.y)*100, 0),
                                                               "%",")",
                                                                 sep=""),
                                     `Total N`=P.n+P.y+C.n+C.y,
                                     P=formatC(p, format="e", digits=1),
                                     `OR [95%CI]`=paste(round(or, 1),' [',ci,']',sep=''),
                                     check.names=FALSE))
# blood pressure medication
P.y <- table(subset(subject.data, Case_status == "PD")$Blood_pressure_med)['Y']
P.n <- table(subset(subject.data, Case_status == "PD")$Blood_pressure_med)['N']
C.y <- table(subset(subject.data, Case_status == "Control")$Blood_pressure_med)['Y']
C.n <- table(subset(subject.data, Case_status == "Control")$Blood_pressure_med)['N']
if (is.na(P.n)){P.n <- 0}
if (is.na(P.y)){P.y <- 0}
if (is.na(C.n)){C.n <- 0}
if (is.na(C.y)){C.y <- 0}
p <- fisher.test(matrix(c(P.y,P.n,C.y,C.n), nrow=2))$p.value
if (any(c(P.y, P.n, C.y, C.n)==0)){
  or <- Prop.or(x=c(P.y,P.n), y=c(C.y,C.n), CImethod='Woolf')$estimate
  ci <- paste(round(Prop.or(x=c(P.y,P.n), y=c(C.y,C.n),
                            CImethod='Woolf')$conf.int[1],1),
              round(Prop.or(x=c(P.y,P.n), y=c(C.y,C.n),
                            CImethod='Woolf')$conf.int[2],1), sep='-')
}else{
  or <- fisher.test(matrix(c(P.y,P.n,C.y,C.n), nrow=2))$estimate
  ci <- paste(round(fisher.test(matrix(c(P.y,P.n,C.y,C.n),
                                       nrow=2))$conf.int[1],1),
              round(fisher.test(matrix(c(P.y,P.n,C.y,C.n),
                                       nrow=2))$conf.int[2],1), sep='-')
}
```

```r
results <- rbind(results, data.frame(Category='',
                                     Metadata ="Blood pressure medication",
                                     `PD N`=P.n+P.y,
                                     `PD summary stats`=paste(P.y,
                                                              " ","(",
                                                              round(P.y/(P.n+P.y)*100, 0),
                                                              "%",")",
                                                                sep=""),
                                     `NHC N`=C.n+C.y,
                                     `NHC summary stats`=paste(C.y,
                                                               " ","(",
                                                               round(C.y/(C.n+C.y)*100, 0),
                                                               "%",")",
                                                                 sep=""),
                                     `Total N`=P.n+P.y+C.n+C.y,
                                     P=formatC(p, format="e", digits=1),
                                     `OR [95%CI]`=paste(round(or, 1),' [',ci,']',sep=''),
                                     check.names=FALSE))
# thyroid medication
P.y <- table(subset(subject.data, Case_status == "PD")$Thyroid_med)['Y']
P.n <- table(subset(subject.data, Case_status == "PD")$Thyroid_med)['N']
C.y <- table(subset(subject.data, Case_status == "Control")$Thyroid_med)['Y']
C.n <- table(subset(subject.data, Case_status == "Control")$Thyroid_med)['N']
if (is.na(P.n)){P.n <- 0}
if (is.na(P.y)){P.y <- 0}
if (is.na(C.n)){C.n <- 0}
if (is.na(C.y)){C.y <- 0}
p <- fisher.test(matrix(c(P.y,P.n,C.y,C.n), nrow=2))$p.value
if (any(c(P.y, P.n, C.y, C.n)==0)){
  or <- Prop.or(x=c(P.y,P.n), y=c(C.y,C.n), CImethod='Woolf')$estimate
  ci <- paste(round(Prop.or(x=c(P.y,P.n), y=c(C.y,C.n),
                            CImethod='Woolf')$conf.int[1],1),
              round(Prop.or(x=c(P.y,P.n), y=c(C.y,C.n),
                            CImethod='Woolf')$conf.int[2],1), sep='-')
}else{
  or <- fisher.test(matrix(c(P.y,P.n,C.y,C.n), nrow=2))$estimate
  ci <- paste(round(fisher.test(matrix(c(P.y,P.n,C.y,C.n),
                                       nrow=2))$conf.int[1],1),
              round(fisher.test(matrix(c(P.y,P.n,C.y,C.n),
                                       nrow=2))$conf.int[2],1), sep='-')
}
results <- rbind(results, data.frame(Category='',
                                     Metadata ="Thyroid medication",
                                     `PD N`=P.n+P.y,
                                     `PD summary stats`=paste(P.y,
                                                              " ","(",
                                                              round(P.y/(P.n+P.y)*100, 0),
                                                              "%",")",
                                                                sep=""),
                                     `NHC N`=C.n+C.y,
                                     `NHC summary stats`=paste(C.y,
                                                               " ","(",
                                                               round(C.y/(C.n+C.y)*100, 0),
                                                               "%",")",
```

```
                                                  sep=""),
                                    `Total N`=P.n+P.y+C.n+C.y,
                                    P=formatC(p, format="e", digits=1),
                                    `OR [95%CI]`=paste(round(or, 1),' [',ci,']',sep=''),
                                    check.names=FALSE))
# asthma or COPD medication
P.y <- table(subset(subject.data, Case_status == "PD")$Asthma_or_COPD_med)['Y']
P.n <- table(subset(subject.data, Case_status == "PD")$Asthma_or_COPD_med)['N']
C.y <- table(subset(subject.data, Case_status == "Control")$Asthma_or_COPD_med)['Y']
C.n <- table(subset(subject.data, Case_status == "Control")$Asthma_or_COPD_med)['N']
if (is.na(P.n)){P.n <- 0}
if (is.na(P.y)){P.y <- 0}
if (is.na(C.n)){C.n <- 0}
if (is.na(C.y)){C.y <- 0}
p <- fisher.test(matrix(c(P.y,P.n,C.y,C.n), nrow=2))$p.value
if (any(c(P.y, P.n, C.y, C.n)==0)){
  or <- Prop.or(x=c(P.y,P.n), y=c(C.y,C.n), CImethod='Woolf')$estimate
  ci <- paste(round(Prop.or(x=c(P.y,P.n), y=c(C.y,C.n),
                    CImethod='Woolf')$conf.int[1],1),
              round(Prop.or(x=c(P.y,P.n), y=c(C.y,C.n),
                    CImethod='Woolf')$conf.int[2],1), sep='-')
}else{
  or <- fisher.test(matrix(c(P.y,P.n,C.y,C.n), nrow=2))$estimate
  ci <- paste(round(fisher.test(matrix(c(P.y,P.n,C.y,C.n),
                         nrow=2))$conf.int[1],1),
              round(fisher.test(matrix(c(P.y,P.n,C.y,C.n),
                         nrow=2))$conf.int[2],1), sep='-')
}
results <- rbind(results, data.frame(Category='',
                                    Metadata ="Asthma or COPD medication",
                                    `PD N`=P.n+P.y,
                                    `PD summary stats`=paste(P.y,
                                                 " ","(",
                                                 round(P.y/(P.n+P.y)*100, 0),
                                                 "%",")",
                                                   sep=""),
                                    `NHC N`=C.n+C.y,
                                    `NHC summary stats`=paste(C.y,
                                                  " ","(",
                                                  round(C.y/(C.n+C.y)*100, 0),
                                                  "%",")",
                                                    sep=""),
                                    `Total N`=P.n+P.y+C.n+C.y,
                                    P=formatC(p, format="e", digits=1),
                                    `OR [95%CI]`=paste(round(or, 1),' [',ci,']',sep=''),
                                    check.names=FALSE))
# diabetes medication
P.y <- table(subset(subject.data, Case_status == "PD")$Diabetes_med)['Y']
P.n <- table(subset(subject.data, Case_status == "PD")$Diabetes_med)['N']
C.y <- table(subset(subject.data, Case_status == "Control")$Diabetes_med)['Y']
C.n <- table(subset(subject.data, Case_status == "Control")$Diabetes_med)['N']
if (is.na(P.n)){P.n <- 0}
if (is.na(P.y)){P.y <- 0}
if (is.na(C.n)){C.n <- 0}
```

```
if (is.na(C.y)){C.y <- 0}
p <- fisher.test(matrix(c(P.y,P.n,C.y,C.n), nrow=2))$p.value
if (any(c(P.y, P.n, C.y, C.n)==0)){
  or <- Prop.or(x=c(P.y,P.n), y=c(C.y,C.n), CImethod='Woolf')$estimate
  ci <- paste(round(Prop.or(x=c(P.y,P.n), y=c(C.y,C.n),
                    CImethod='Woolf')$conf.int[1],1),
              round(Prop.or(x=c(P.y,P.n), y=c(C.y,C.n),
                    CImethod='Woolf')$conf.int[2],1), sep='-')
}else{
  or <- fisher.test(matrix(c(P.y,P.n,C.y,C.n), nrow=2))$estimate
  ci <- paste(round(fisher.test(matrix(c(P.y,P.n,C.y,C.n),
                               nrow=2))$conf.int[1],1),
              round(fisher.test(matrix(c(P.y,P.n,C.y,C.n),
                               nrow=2))$conf.int[2],1), sep='-')
}
results <- rbind(results, data.frame(Category='',
                                     Metadata ="Diabetes medication",
                                     `PD N`=P.n+P.y,
                                     `PD summary stats`=paste(P.y,
                                                         " ","(",
                                                         round(P.y/(P.n+P.y)*100, 0),
                                                         "%",")",
                                                           sep=""),
                                     `NHC N`=C.n+C.y,
                                     `NHC summary stats`=paste(C.y,
                                                          " ","(",
                                                          round(C.y/(C.n+C.y)*100, 0),
                                                          "%",")",
                                                            sep=""),
                                     `Total N`=P.n+P.y+C.n+C.y,
                                     P=formatC(p, format="e", digits=1),
                                     `OR [95%CI]`=paste(round(or, 1),' [',ci,']',sep=''),
                                     check.names=FALSE))
# pain medication
P.y <- table(subset(subject.data, Case_status == "PD")$Pain_med)['Y']
P.n <- table(subset(subject.data, Case_status == "PD")$Pain_med)['N']
C.y <- table(subset(subject.data, Case_status == "Control")$Pain_med)['Y']
C.n <- table(subset(subject.data, Case_status == "Control")$Pain_med)['N']
if (is.na(P.n)){P.n <- 0}
if (is.na(P.y)){P.y <- 0}
if (is.na(C.n)){C.n <- 0}
if (is.na(C.y)){C.y <- 0}
p <- fisher.test(matrix(c(P.y,P.n,C.y,C.n), nrow=2))$p.value
if (any(c(P.y, P.n, C.y, C.n)==0)){
  or <- Prop.or(x=c(P.y,P.n), y=c(C.y,C.n), CImethod='Woolf')$estimate
  ci <- paste(round(Prop.or(x=c(P.y,P.n), y=c(C.y,C.n),
                    CImethod='Woolf')$conf.int[1],1),
              round(Prop.or(x=c(P.y,P.n), y=c(C.y,C.n),
                    CImethod='Woolf')$conf.int[2],1), sep='-')
}else{
  or <- fisher.test(matrix(c(P.y,P.n,C.y,C.n), nrow=2))$estimate
  ci <- paste(round(fisher.test(matrix(c(P.y,P.n,C.y,C.n),
                               nrow=2))$conf.int[1],1),
              round(fisher.test(matrix(c(P.y,P.n,C.y,C.n),
```

```
                                        nrow=2))$conf.int[2],1), sep='-')
}
results <- rbind(results, data.frame(Category='',
                                      Metadata ="Pain medication",
                                      `PD N`=P.n+P.y,
                                      `PD summary stats`=paste(P.y,
                                                               " ","(",
                                                               round(P.y/(P.n+P.y)*100, 0),
                                                               "%",")",
                                                                 sep=""),
                                      `NHC N`=C.n+C.y,
                                      `NHC summary stats`=paste(C.y,
                                                                " ","(",
                                                                round(C.y/(C.n+C.y)*100, 0),
                                                                "%",")",
                                                                  sep=""),
                                      `Total N`=P.n+P.y+C.n+C.y,
                                      P=formatC(p, format="e", digits=1),
                                      `OR [95%CI]`=paste(round(or, 1),' [',ci,']',sep=''),
                                      check.names=FALSE))
# depression, anxiety, mood medication
P.y <- table(subset(subject.data, Case_status == "PD")$Depression_anxiety_mood_med)['Y']
P.n <- table(subset(subject.data, Case_status == "PD")$Depression_anxiety_mood_med)['N']
C.y <- table(subset(subject.data, Case_status == "Control")$Depression_anxiety_mood_med)['Y']
C.n <- table(subset(subject.data, Case_status == "Control")$Depression_anxiety_mood_med)['N']
if (is.na(P.n)){P.n <- 0}
if (is.na(P.y)){P.y <- 0}
if (is.na(C.n)){C.n <- 0}
if (is.na(C.y)){C.y <- 0}
p <- fisher.test(matrix(c(P.y,P.n,C.y,C.n), nrow=2))$p.value
if (any(c(P.y, P.n, C.y, C.n)==0)){
  or <- Prop.or(x=c(P.y,P.n), y=c(C.y,C.n), CImethod='Woolf')$estimate
  ci <- paste(round(Prop.or(x=c(P.y,P.n), y=c(C.y,C.n),
                            CImethod='Woolf')$conf.int[1],1),
              round(Prop.or(x=c(P.y,P.n), y=c(C.y,C.n),
                            CImethod='Woolf')$conf.int[2],1), sep='-')
}else{
  or <- fisher.test(matrix(c(P.y,P.n,C.y,C.n), nrow=2))$estimate
  ci <- paste(round(fisher.test(matrix(c(P.y,P.n,C.y,C.n),
                                        nrow=2))$conf.int[1],1),
              round(fisher.test(matrix(c(P.y,P.n,C.y,C.n),
                                        nrow=2))$conf.int[2],1), sep='-')
}
results <- rbind(results, data.frame(Category='',
                                      Metadata ="Depression, anxiety, mood medication",
                                      `PD N`=P.n+P.y,
                                      `PD summary stats`=paste(P.y,
                                                               " ","(",
                                                               round(P.y/(P.n+P.y)*100, 0),
                                                               "%",")",
                                                                 sep=""),
                                      `NHC N`=C.n+C.y,
                                      `NHC summary stats`=paste(C.y,
                                                                " ","(",
```

```
                                                                          round(C.y/(C.n+C.y)*100, 0),
                                                                          "%",")",
                                                                            sep=""),
                                                   `Total N`=P.n+P.y+C.n+C.y,
                                                   P=formatC(p, format="e", digits=1),
                                                   `OR [95%CI]`=paste(round(or, 1),' [',ci,']',sep=''),
                                                   check.names=FALSE))
# birth control or estrogen (females only)
P.y <- table(subset(subject.data,
     Case_status == "PD" & Sex == "F")$Birth_control_or_estrogen)['Y']
P.n <- table(subset(subject.data,
     Case_status == "PD" & Sex == "F")$Birth_control_or_estrogen)['N']
C.y <- table(subset(subject.data,
     Case_status == "Control" & Sex == "F")$Birth_control_or_estrogen)['Y']
C.n <- table(subset(subject.data,
     Case_status == "Control" & Sex == "F")$Birth_control_or_estrogen)['N']
if (is.na(P.n)){P.n <- 0}
if (is.na(P.y)){P.y <- 0}
if (is.na(C.n)){C.n <- 0}
if (is.na(C.y)){C.y <- 0}
p <- fisher.test(matrix(c(P.y,P.n,C.y,C.n), nrow=2))$p.value
if (any(c(P.y, P.n, C.y, C.n)==0)){
  or <- Prop.or(x=c(P.y,P.n), y=c(C.y,C.n), CImethod='Woolf')$estimate
  ci <- paste(round(Prop.or(x=c(P.y,P.n), y=c(C.y,C.n),
                          CImethod='Woolf')$conf.int[1],1),
              round(Prop.or(x=c(P.y,P.n), y=c(C.y,C.n),
                          CImethod='Woolf')$conf.int[2],1), sep='-')
}else{
  or <- fisher.test(matrix(c(P.y,P.n,C.y,C.n), nrow=2))$estimate
  ci <- paste(round(fisher.test(matrix(c(P.y,P.n,C.y,C.n),
                                     nrow=2))$conf.int[1],1),
              round(fisher.test(matrix(c(P.y,P.n,C.y,C.n),
                                     nrow=2))$conf.int[2],1), sep='-')
}
results <- rbind(results, data.frame(Category='',
                                     Metadata ="Birth control or estrogen (females)",
                                     `PD N`=P.n+P.y,
                                     `PD summary stats`=paste(P.y,
                                                         " ","(",
                                                         round(P.y/(P.n+P.y)*100, 0),
                                                         "%",")",
                                                           sep=""),
                                     `NHC N`=C.n+C.y,
                                     `NHC summary stats`=paste(C.y,
                                                          " ","(",
                                                          round(C.y/(C.n+C.y)*100, 0),
                                                          "%",")",
                                                            sep=""),
                                     `Total N`=P.n+P.y+C.n+C.y,
                                     P=formatC(p, format="e", digits=1),
                                     `OR [95%CI]`=paste(round(or, 1),' [',ci,']',sep=''),
                                     check.names=FALSE))
# antihistamines
P.y <- table(subset(subject.data, Case_status == "PD")$Antihistamines)['Y']
```

```
P.n <- table(subset(subject.data, Case_status == "PD")$Antihistamines)['N']
C.y <- table(subset(subject.data, Case_status == "Control")$Antihistamines)['Y']
C.n <- table(subset(subject.data, Case_status == "Control")$Antihistamines)['N']
if (is.na(P.n)){P.n <- 0}
if (is.na(P.y)){P.y <- 0}
if (is.na(C.n)){C.n <- 0}
if (is.na(C.y)){C.y <- 0}
p <- fisher.test(matrix(c(P.y,P.n,C.y,C.n), nrow=2))$p.value
if (any(c(P.y, P.n, C.y, C.n)==0)){
  or <- Prop.or(x=c(P.y,P.n), y=c(C.y,C.n), CImethod='Woolf')$estimate
  ci <- paste(round(Prop.or(x=c(P.y,P.n), y=c(C.y,C.n),
                        CImethod='Woolf')$conf.int[1],1),
              round(Prop.or(x=c(P.y,P.n), y=c(C.y,C.n),
                        CImethod='Woolf')$conf.int[2],1), sep='-')
}else{
  or <- fisher.test(matrix(c(P.y,P.n,C.y,C.n), nrow=2))$estimate
  ci <- paste(round(fisher.test(matrix(c(P.y,P.n,C.y,C.n),
                                   nrow=2))$conf.int[1],1),
              round(fisher.test(matrix(c(P.y,P.n,C.y,C.n),
                                   nrow=2))$conf.int[2],1), sep='-')
}
results <- rbind(results, data.frame(Category='',
                                     Metadata ="Antihistamines",
                                     `PD N`=P.n+P.y,
                                     `PD summary stats`=paste(P.y,
                                                         " ","(",
                                                         round(P.y/(P.n+P.y)*100, 0),
                                                         "%",")",
                                                           sep=""),
                                     `NHC N`=C.n+C.y,
                                     `NHC summary stats`=paste(C.y,
                                                          " ","(",
                                                          round(C.y/(C.n+C.y)*100, 0),
                                                          "%",")",
                                                            sep=""),
                                     `Total N`=P.n+P.y+C.n+C.y,
                                     P=formatC(p, format="e", digits=1),
                                     `OR [95%CI]`=paste(round(or, 1),' [',ci,']',sep=''),
                                     check.names=FALSE))
# Co Q 10
P.y <- table(subset(subject.data, Case_status == "PD")$Co_Q_10)['Y']
P.n <- table(subset(subject.data, Case_status == "PD")$Co_Q_10)['N']
C.y <- table(subset(subject.data, Case_status == "Control")$Co_Q_10)['Y']
C.n <- table(subset(subject.data, Case_status == "Control")$Co_Q_10)['N']
if (is.na(P.n)){P.n <- 0}
if (is.na(P.y)){P.y <- 0}
if (is.na(C.n)){C.n <- 0}
if (is.na(C.y)){C.y <- 0}
p <- fisher.test(matrix(c(P.y,P.n,C.y,C.n), nrow=2))$p.value
if (any(c(P.y, P.n, C.y, C.n)==0)){
  or <- Prop.or(x=c(P.y,P.n), y=c(C.y,C.n), CImethod='Woolf')$estimate
  ci <- paste(round(Prop.or(x=c(P.y,P.n), y=c(C.y,C.n),
                        CImethod='Woolf')$conf.int[1],1),
              round(Prop.or(x=c(P.y,P.n), y=c(C.y,C.n),
```

```
                            CImethod='Woolf')$conf.int[2],1), sep='-')
}else{
  or <- fisher.test(matrix(c(P.y,P.n,C.y,C.n), nrow=2))$estimate
  ci <- paste(round(fisher.test(matrix(c(P.y,P.n,C.y,C.n),
                                       nrow=2))$conf.int[1],1),
              round(fisher.test(matrix(c(P.y,P.n,C.y,C.n),
                                       nrow=2))$conf.int[2],1), sep='-')
}
results <- rbind(results, data.frame(Category='',
                                     Metadata ="Co-Q 10",
                                     `PD N`=P.n+P.y,
                                     `PD summary stats`=paste(P.y,
                                                              " ","(",
                                                              round(P.y/(P.n+P.y)*100, 0),
                                                              "%",")",
                                                                sep=""),
                                     `NHC N`=C.n+C.y,
                                     `NHC summary stats`=paste(C.y,
                                                               " ","(",
                                                               round(C.y/(C.n+C.y)*100, 0),
                                                               "%",")",
                                                                 sep=""),
                                     `Total N`=P.n+P.y+C.n+C.y,
                                     P=formatC(p, format="e", digits=1),
                                     `OR [95%CI]`=paste(round(or, 1),' [',ci,']',sep=''),
                                     check.names=FALSE))
# sleep aid
P.y <- table(subset(subject.data, Case_status == "PD")$Sleep_aid)['Y']
P.n <- table(subset(subject.data, Case_status == "PD")$Sleep_aid)['N']
C.y <- table(subset(subject.data, Case_status == "Control")$Sleep_aid)['Y']
C.n <- table(subset(subject.data, Case_status == "Control")$Sleep_aid)['N']
if (is.na(P.n)){P.n <- 0}
if (is.na(P.y)){P.y <- 0}
if (is.na(C.n)){C.n <- 0}
if (is.na(C.y)){C.y <- 0}
p <- fisher.test(matrix(c(P.y,P.n,C.y,C.n), nrow=2))$p.value
if (any(c(P.y, P.n, C.y, C.n)==0)){
  or <- Prop.or(x=c(P.y,P.n), y=c(C.y,C.n), CImethod='Woolf')$estimate
  ci <- paste(round(Prop.or(x=c(P.y,P.n), y=c(C.y,C.n),
                            CImethod='Woolf')$conf.int[1],1),
              round(Prop.or(x=c(P.y,P.n), y=c(C.y,C.n),
                            CImethod='Woolf')$conf.int[2],1), sep='-')
}else{
  or <- fisher.test(matrix(c(P.y,P.n,C.y,C.n), nrow=2))$estimate
  ci <- paste(round(fisher.test(matrix(c(P.y,P.n,C.y,C.n),
                                       nrow=2))$conf.int[1],1),
              round(fisher.test(matrix(c(P.y,P.n,C.y,C.n),
                                       nrow=2))$conf.int[2],1), sep='-')
}
results <- rbind(results, data.frame(Category='',
                                     Metadata ="Sleep aid",
                                     `PD N`=P.n+P.y,
                                     `PD summary stats`=paste(P.y,
                                                              " ","(",
```

```
                                                              round(P.y/(P.n+P.y)*100, 0),
                                                              "%",")",
                                                                sep=""),
                                     `NHC N`=C.n+C.y,
                                     `NHC summary stats`=paste(C.y,
                                                               " ","(",
                                                               round(C.y/(C.n+C.y)*100, 0),
                                                               "%",")",
                                                                 sep=""),
                                     `Total N`=P.n+P.y+C.n+C.y,
                                     P=formatC(p, format="e", digits=1),
                                     `OR [95%CI]`=paste(round(or, 1),' [',ci,']',sep=''),
                                     check.names=FALSE))
# add variable numbers
results <- data.frame(Index=c('',1:(nrow(results)-1)), results, check.names=FALSE)

# write out results
# create workbook
wb <- createWorkbook()
# add worksheet, write data, and format output
addWorksheet(wb, 'Subject characteristics')
writeData(wb, 'Subject characteristics', results, keepNA=TRUE)
setColWidths(wb, 'Subject characteristics', cols=seq_len(ncol(results)),
             widths=c(10,20,55,10,15,10,15,10,10,12)) ### format cells
addStyle(wb, 'Subject characteristics', cols=seq_len(ncol(results)),
         rows=1:(nrow(results)+1), gridExpand=TRUE, style=center, stack=TRUE)
mergeCells(wb, 'Subject characteristics', cols=2, rows=2:4)
mergeCells(wb, 'Subject characteristics', cols=2, rows=5:7)
mergeCells(wb, 'Subject characteristics', cols=2, rows=8:10)
mergeCells(wb, 'Subject characteristics', cols=2, rows=11:18)
mergeCells(wb, 'Subject characteristics', cols=2, rows=19:25)
mergeCells(wb, 'Subject characteristics', cols=2, rows=26:27)
mergeCells(wb, 'Subject characteristics', cols=2, rows=28:36)
mergeCells(wb, 'Subject characteristics', cols=2, rows=37:55)
addStyle(wb, 'Subject characteristics', cols=seq_len(ncol(results)),
         rows=1, style=bold, stack=TRUE) ### font
addStyle(wb, 'Subject characteristics', cols=seq_len(ncol(results)),
         rows=c(1,2,(nrow(results)+2)), ### borders
         gridExpand=TRUE, style=horizontal_border_med, stack=TRUE)
addStyle(wb, 'Subject characteristics', cols=seq_len(ncol(results)),
         rows=c(5,8,11,19,26,28,37), gridExpand=TRUE,
         style=horizontal_border_thin, stack=TRUE)
# convert numbers from strings back to numbers
convertNum(results, wb, 'Subject characteristics', TRUE)
# save workbook
saveWorkbook(wb, 'PDShotgunAnalysis_out/1.Metadata/Subject_characteristics_PDvsNHC.xlsx',
             overwrite=TRUE)
```

Analyses of species and genera

Preparing relative abundance and count data for downstream analyses

```r
#### PREPARE RELATIVE ABUNDANCE AND COUNT DATA ####

# read in metadata
metadata <- data.frame(read_xlsx('Source_Data.xlsx', sheet='subject_metadata'))
rownames(metadata) <- metadata$sample_name

# read in tables that were previously generated by taxonomic profiling
abun <- data.frame(read_xlsx('Source_Data.xlsx', sheet='metaphlan_counts'))

ra <- data.frame(read_xlsx('Source_Data.xlsx', sheet='metaphlan_rel_ab'))

# order same as metadata
abun <- abun[,c('clade_name',metadata$sample_name)]

ra <- ra[,c('clade_name',metadata$sample_name)]

# make table sample x feature
rownames(abun) <- abun$clade_name
abun <- data.frame(t(abun[,-1]), check.names=FALSE)

rownames(ra) <- ra$clade_name
ra <- data.frame(t(ra[,-1]), check.names=FALSE)

# compile count data into phyloseq objects for species and genus taxonomic levels
abun.sub <- abun[,grep("s__|UNKNOWN", colnames(abun))]
abun.ps.s <- phyloseq(otu_table(as.matrix(abun.sub), taxa_are_rows=FALSE),
                      sample_data(metadata),
                      tax_table(as.matrix(
                      data.frame(Kingdom=sapply(strsplit(colnames(abun.sub), "\\|"),
                                                function(x){x[1]}),
                                 Phylum=sapply(strsplit(colnames(abun.sub), "\\|"),
                                                function(x){x[2]}),
                                 Class=sapply(strsplit(colnames(abun.sub), "\\|"),
                                                function(x){x[3]}),
                                 Order=sapply(strsplit(colnames(abun.sub), "\\|"),
                                                function(x){x[4]}),
                                 Family=sapply(strsplit(colnames(abun.sub), "\\|"),
                                                function(x){x[5]}),
                                 Genus=sapply(strsplit(colnames(abun.sub), "\\|"),
                                                function(x){x[6]}),
                                 Species=sapply(strsplit(colnames(abun.sub), "\\|"),
                                                function(x){x[7]}),
                                 check.names=FALSE, row.names=colnames(abun.sub)))))
abun.sub <- abun[,intersect(grep("s__", colnames(abun), invert=TRUE),
                            grep("g__|UNKNOWN", colnames(abun)))]
abun.ps.g <- phyloseq(otu_table(as.matrix(abun.sub), taxa_are_rows=FALSE),
                      sample_data(metadata),
                      tax_table(as.matrix(
                      data.frame(Kingdom=sapply(strsplit(colnames(abun.sub), "\\|"),
```

```
                                                function(x){x[1]}),
                                 Phylum=sapply(strsplit(colnames(abun.sub), "\\|"),
                                                function(x){x[2]}),
                                 Class=sapply(strsplit(colnames(abun.sub), "\\|"),
                                                function(x){x[3]}),
                                 Order=sapply(strsplit(colnames(abun.sub), "\\|"),
                                                function(x){x[4]}),
                                 Family=sapply(strsplit(colnames(abun.sub), "\\|"),
                                                function(x){x[5]}),
                                 Genus=sapply(strsplit(colnames(abun.sub), "\\|"),
                                                function(x){x[6]}),
                                 Species=sapply(strsplit(colnames(abun.sub), "\\|"),
                                                function(x){x[7]}),
                                 check.names=FALSE, row.names=colnames(abun.sub)))))

# compile relative abundance data into phyloseq objects for species and
# genus taxonomic levels
ra.sub <- ra[,grep("s__|UNKNOWN", colnames(ra))]
ra.ps.s <- phyloseq(otu_table(as.matrix(ra.sub), taxa_are_rows=FALSE),
                      sample_data(metadata),
                      tax_table(as.matrix(
                      data.frame(Kingdom=sapply(strsplit(colnames(ra.sub), "\\|"),
                                                function(x){x[1]}),
                                 Phylum=sapply(strsplit(colnames(ra.sub), "\\|"),
                                                function(x){x[2]}),
                                 Class=sapply(strsplit(colnames(ra.sub), "\\|"),
                                                function(x){x[3]}),
                                 Order=sapply(strsplit(colnames(ra.sub), "\\|"),
                                                function(x){x[4]}),
                                 Family=sapply(strsplit(colnames(ra.sub), "\\|"),
                                                function(x){x[5]}),
                                 Genus=sapply(strsplit(colnames(ra.sub), "\\|"),
                                                function(x){x[6]}),
                                 Species=sapply(strsplit(colnames(ra.sub), "\\|"),
                                                function(x){x[7]}),
                                 check.names=FALSE, row.names=colnames(ra.sub)))))
ra.sub <- ra[,intersect(grep("s__", colnames(ra), invert=TRUE),
                        grep("g__|UNKNOWN", colnames(ra)))]
ra.ps.g <- phyloseq(otu_table(as.matrix(ra.sub), taxa_are_rows=FALSE),
                      sample_data(metadata),
                      tax_table(as.matrix(
                      data.frame(Kingdom=sapply(strsplit(colnames(ra.sub), "\\|"),
                                                function(x){x[1]}),
                                 Phylum=sapply(strsplit(colnames(ra.sub), "\\|"),
                                                function(x){x[2]}),
                                 Class=sapply(strsplit(colnames(ra.sub), "\\|"),
                                                function(x){x[3]}),
                                 Order=sapply(strsplit(colnames(ra.sub), "\\|"),
                                                function(x){x[4]}),
                                 Family=sapply(strsplit(colnames(ra.sub), "\\|"),
                                                function(x){x[5]}),
                                 Genus=sapply(strsplit(colnames(ra.sub), "\\|"),
                                                function(x){x[6]}),
                                 Species=sapply(strsplit(colnames(ra.sub), "\\|"),
```

```
                                                    function(x){x[7]}),
                                       check.names=FALSE, row.names=colnames(ra.sub)))))
```

Principal Component Analysis

To observe inter-sample differences in gut microbiome compositions (beta-diversity), principal component analysis (PCA) was performed to visually inspect differences in microbiome compositions between samples.

- To perform the PCA, species counts from MetaPhlAn were transformed using the clr transformation (formula: `log(x+1)-mean(log(x+1))` where `x` is a vector of all the species counts for a sample). PCA was then performed using the `prcomp` function with default parameters. PC1 and PC2 were then plotted with convex hull areas for each group using the `autoplot` function from `ggfortify`.

```
#### PERFORM PCA ####

# make sure taxa that are all 0 are removed, then transform abundances to clr
abun.clr <- transform_sample_counts(filter_taxa(abun.ps.s, function(x){sum(x>0)>0}, TRUE),
                                    function(x){log(x+1)-mean(log(x+1))})
abun.clr <- prune_taxa(taxa_names(abun.clr)[taxa_names(abun.clr) != 'UNKNOWN'], abun.clr)

# perform PCA
pca <- prcomp(otu_table(abun.clr))

# plot PC1 and PC2 coloring by case status
suppress(
g <- autoplot(pca, data=data.frame(sample_data(abun.clr)), colour='Case_status',
              shape='Case_status', scale=FALSE, frame=TRUE) +
  theme_bw() +
  scale_colour_manual(labels=c('NHC','PD'), values=c("#E69F00", "#00BFC4")) +
  scale_fill_manual(labels=c('NHC','PD'), values=c("#E69F00", "#00BFC4")) +
  scale_shape_manual(labels=c('NHC','PD'), values=c(17,16)) +
  labs(fill="Case status", color="Case status", shape="Case status")
)
ggsave('PDShotgunAnalysis_out/2.Gut_microbiome_composition/PCA_case_status.pdf',
       g, device='pdf', width=5, height=5)
```

- To observe the influence of rarer species on the PCA, PCA was performed and PC 1 and 2 plotted again after excluding species that were detected in <5% of samples.

```
#### PERFORM PCA EXCLUDING RARE SPECIES ####

# remove taxa that are found in <5% of samples, then transform abundances to clr
abun.clr.filt <- transform_sample_counts(
                    filter_taxa(abun.ps.s,
                                function(x){sum(x>0)>(0.05*nsamples(abun.ps.s))}, TRUE),
                    function(x){log(x+1)-mean(log(x+1))})
abun.clr.filt <- prune_taxa(
                    taxa_names(abun.clr.filt)[taxa_names(abun.clr.filt) != 'UNKNOWN'],
                    abun.clr.filt)

# perform PCA
pca <- prcomp(otu_table(abun.clr.filt))

# plot PC1 and PC2 coloring by case status
suppress(
```

```
g <- autoplot(pca, data=data.frame(sample_data(abun.clr.filt)), colour='Case_status',
              shape='Case_status', scale=FALSE, frame=TRUE) +
  theme_bw() +
  scale_colour_manual(labels=c('NHC','PD'), values=c("#E69F00", "#00BFC4")) +
  scale_fill_manual(labels=c('NHC','PD'), values=c("#E69F00", "#00BFC4")) +
  scale_shape_manual(labels=c('NHC','PD'), values=c(17,16)) +
  labs(fill="Case status", color="Case status", shape="Case status")
)
ggsave('PDShotgunAnalysis_out/2.Gut_microbiome_composition/PCA_case_status_filtered.pdf',
       g, device='pdf', width=5, height=5)
```

PERMANOVA and PERMDISP

To test if case status significantly associates with inter-sample variation in microbiome compositions (beta-diversity), permutational multivariate analysis of variance (PERMANOVA) was performed.

- PERMANOVA was performed using the function `adonis2` from `vegan` adjusting for stool sampling method, and total sequence count per sample (standardized using the `scale` function). All variables were adjusted for one another in a marginal model by setting `by='margin'`.
- To test if significant results of PERMANOVA were due to differences in heterogeneity of dispersions between groups, a permutation-based test of multivariate homogeneity of group dispersions (PERMDISP) was performed using the `betadisper` (setting `type='median'`) and `permutest` functions from `vegan` to perform the test.
- Aitchison distance (Euclidean distance of clr transformed data) was used as the distance matrix outcome for both PERMANOVA and PERMDISP (calculated using `vegdist` from `vegan` specifying `method='euclidean'`).
- Significance of permutational tests were determined using 9999 permutations.
- PERMANOVA and PERMDISP were performed once with all species and again for species found in >5% of samples.

```
#### PERFORM PERMANOVA & PERMDISP ####

# standardize total sequence count
sample_data(abun.clr)$seqs_scaled <- scale(sample_data(abun.clr)$total_sequences)

# calculate euclidean distances on clr transformed data (Aitchison distances)
aitch.dist <- vegdist(otu_table(abun.clr), method='euclidean')

# run adonis2 (PERMANOVA) marginal model and 9,999 permutations
set.seed(1234)
fit <- adonis2(aitch.dist ~ Case_status + seqs_scaled + collection_method,
               data=data.frame(sample_data(abun.clr)), by='margin', perm=9999)

# run betadisper and permutest (PERMDISP) with euclidean distance
set.seed(1234)
disp <- permutest(betadisper(aitch.dist,
                             sample_data(abun.clr)$Case_status,
                             type='median'),
                  permutations=9999)
disp.r2 <- disp$tab['Groups','Sum Sq']/(disp$tab['Groups','Sum Sq']+
                                        disp$tab['Residuals','Sum Sq'])

#### PERFORM PERMANOVA & PERMDISP EXCLUDING RARE SPECIES ####

```

```
# standardize total sequence count
sample_data(abun.clr.filt)$seqs_scaled <- scale(sample_data(abun.clr.filt)$total_sequences)

# calculate euclidean distances on clr transformed data (Aitchison distances)
aitch.dist <- vegdist(otu_table(abun.clr.filt), method='euclidean')

# run adonis2 (PERMANOVA) marginal model and 9,999 permutations
set.seed(1234)
fit.filt <- adonis2(aitch.dist ~ Case_status + seqs_scaled + collection_method,
                    data=data.frame(sample_data(abun.clr.filt)), by='margin', perm=9999)

# run betadisper and permutest (PERMDISP) with euclidean distance
set.seed(1234)
disp.filt <- permutest(betadisper(aitch.dist,
                                  sample_data(abun.clr.filt)$Case_status,
                                  type='median'),
                       permutations=9999)
disp.r2.filt <- disp.filt$tab['Groups','Sum Sq']/(disp.filt$tab['Groups','Sum Sq']+
                                                  disp.filt$tab['Residuals','Sum Sq'])

# coalesce the results
results <- data.frame(
  `Included species`=c('all detected species','',''),
  Variable=c('case status','sequence depth (standardized)','collection method'),
  `PERMANOVA Results`=c(paste('R2=',round(fit$R2[1],3),', P<',
                              formatC(fit$`Pr(>F)`[1],
                                      format='e',digits=0),sep=''),
                        paste('R2=',round(fit$R2[2],3),', P<',
                              formatC(fit$`Pr(>F)`[2],
                                      format='e',digits=0),sep=''),
                        paste('R2=',round(fit$R2[3],3),', P=',
                              formatC(fit$`Pr(>F)`[3],
                                      format='e',digits=0),sep='')),
  `PERMDISP Results`=c(paste('R2=',round(disp.r2,3),', P<',
                             formatC(disp$tab$`Pr(>F)`[1],
                                     format='e',digits=0),sep=''),
                       '-','-'),
  check.names=FALSE)

results <- rbind(results, data.frame(
  `Included species`=c('species in >5% samples','',''),
  Variable=c('case status','sequence depth (standardized)','collection method'),
  `PERMANOVA Results`=c(paste('R2=',round(fit.filt$R2[1],3),', P<',
                              formatC(fit.filt$`Pr(>F)`[1],
                                      format='e',digits=0),sep=''),
                        paste('R2=',round(fit.filt$R2[2],3),', P<',
                              formatC(fit.filt$`Pr(>F)`[2],
                                      format='e',digits=0),sep=''),
                        paste('R2=',round(fit.filt$R2[3],3),', P=',
                              round(fit.filt$`Pr(>F)`[3],2),sep='')),
  `PERMDISP Results`=c(paste('R2=',round(disp.r2.filt,3),', P<',
                             formatC(disp.filt$tab$`Pr(>F)`[1],
                                     format='e',digits=0),sep=''),
                       '-','-'),
```

```
  check.names=FALSE))

# write results
# create worbook
wb <- createWorkbook()
# add worksheet, write data, and format output
addWorksheet(wb, 'PERMANOVA PERMDISP')
writeData(wb, 'PERMANOVA PERMDISP', results, keepNA=TRUE)
setColWidths(wb, 'PERMANOVA PERMDISP', cols=seq_len(ncol(results)),
             widths=rep(20,ncol(results))) ### format cells
addStyle(wb, 'PERMANOVA PERMDISP', cols=seq_len(ncol(results)),
         rows=1:(nrow(results)+1), gridExpand=TRUE, style=center, stack=TRUE)
addStyle(wb, 'PERMANOVA PERMDISP', cols=seq_len(ncol(results)),
         rows=1, style=bold, stack=TRUE) ### font
addStyle(wb, 'PERMANOVA PERMDISP', cols=seq_len(ncol(results)),
         rows=c(1,2,(nrow(results)+2)), ### borders
         gridExpand=TRUE, style=horizontal_border_med, stack=TRUE)
# save workbook
saveWorkbook(wb,
   'PDShotgunAnalysis_out/2.Gut_microbiome_composition/PERMANOVA_PERMDISP_PDvsNHC.xlsx',
   overwrite=TRUE)
```

Enterotype analysis: PD vs NHC

To determine if PD patients had a different distribution of enterotype frequencies than NHC, enterotype profiling was performed using the web-based EMBL enterotype classifier, then differences in overall enterotype frequencies between PD and NHC were tested. The enterotype classifier uses the orignal entertoype definitions, classifying each sample as the enterotype *Bacteroides*, *Firmicutes*, or *Prevotella*.

- To perform enterotype profiling, the MetaPhlAn relative abundance file was subsetted for only genus level entries, then uploaded to the web-based EMBL enterotype classifier. The raw results were downloaded (see Source Data file for enterotype designations) and used to perform analyses, and generate a mosaic plot. Only subjects that were detected by the EMBL classifier as compositionally similar to the training data used to build the classifier (`Within_ET_space` is `TRUE`) were used for analyses (N = 450; 284 PD, 166 NHC).
- Differences in enterotype frequencies between PD and NHC were tested using the `chisq.test` function to perform Pearson's Chi-squared test.
- Relative predispositional effect (RPE) of enterotypes was then investigated to determine what enterotype(s) were driving the difference between PD vs NHC.
- Odds ratios and corresponding significance for the effect driving enterotype(s) were calculated using Fisher's exact test via the `fisher.test` function.

```
#### ENTEROTYPE ANALYSIS ####

# read in enterotype profiles
et <- data.frame(read_xlsx('Source_Data.xlsx', sheet='enterotypes', skip=2))

# make a quick column for case status
et$case_status <- NA
et$case_status[grep('P', et$sample_name)] <- "PD"
et$case_status[grep('C', et$sample_name)] <- "NHC"

# subset for samples with 'Within_ET_space' equal to TRUE
et <- et[et$Within_ET_space == TRUE,]
```

```r

# perform chi-squared test for overall difference in enterotype distribution
x2.test.res <- chisq.test(table(et$case_status, et$ET))

#### RPE ####

### round 1 ###
# calculate chi-squared statistics for each PD enterotype using
# NHC frequencies to calculate expected values
O <- table(et$case_status, et$ET)['PD','ET_B']
E <- sum(table(et$case_status, et$ET)['PD',])*
     (table(et$case_status, et$ET)['NHC','ET_B']/
      sum(table(et$case_status, et$ET)['NHC',]))
x2.ET_B <- (O - E)^2/E

O <- table(et$case_status, et$ET)['PD','ET_F']
E <- sum(table(et$case_status, et$ET)['PD',])*
     (table(et$case_status, et$ET)['NHC','ET_F']/
      sum(table(et$case_status, et$ET)['NHC',]))
x2.ET_F <- (O - E)^2/E

O <- table(et$case_status, et$ET)['PD','ET_P']
E <- sum(table(et$case_status, et$ET)['PD',])*
     (table(et$case_status, et$ET)['NHC','ET_P']/
      sum(table(et$case_status, et$ET)['NHC',]))
x2.ET_P <- (O - E)^2/E

x2.list <- c(x2.ET_B, x2.ET_F, x2.ET_P)

# calculate total chi-squared statistic and p-value
x2.1 <- sum(x2.list)
x2.p1 <- pchisq(q=x2.1, df=length(x2.list)-1, lower.tail=FALSE)

### round 2 ###
# calculate chi-squared statistics for each PD enterotype using
# NHC frequencies to calculate expected values (after removing Firmicutes
# enterotype that had max chi-squared statistic from last round)
O <- table(et$case_status, et$ET)['PD','ET_B']
E <- sum(table(et$case_status, et$ET)['PD',c('ET_B','ET_P')])*
     (table(et$case_status, et$ET)['NHC','ET_B']/
      sum(table(et$case_status, et$ET)['NHC',c('ET_B','ET_P')]))
x2.ET_B <- (O - E)^2/E

O <- table(et$case_status, et$ET)['PD','ET_P']
E <- sum(table(et$case_status, et$ET)['PD',c('ET_B','ET_P')])*
     (table(et$case_status, et$ET)['NHC','ET_P']/
      sum(table(et$case_status, et$ET)['NHC',c('ET_B','ET_P')]))
x2.ET_P <- (O - E)^2/E

x2.list <- c(x2.ET_B, x2.ET_P)

# calculate total chi-squared statistic and p-value
x2.2 <- sum(x2.list, na.rm=TRUE)
x2.p2 <- pchisq(q=x2.2, df=length(x2.list)-1, lower.tail=FALSE)
```

```r

#### RPE END ####

# calculate odds ratio and significance for individual enterotypes
fisher.ET_F <- fisher.test(table(et$case_status,
                                 dplyr::recode(et$ET, ET_F='1', ET_B='0', ET_P='0')))
fisher.ET_P <- fisher.test(table(et$case_status,
                                 dplyr::recode(et$ET, ET_F='0', ET_P='1', ET_B='0')))
fisher.ET_P2 <- fisher.test(table(et$case_status[et$ET != 'ET_F'],
                                  dplyr::recode(et$ET[et$ET != 'ET_F'], ET_P='1', ET_B='0')))

# coalesce results
results <- data.frame(`Case status`=names(rev(table(et$case_status))),
                      `N Total`=as.vector(rev(table(et$case_status))),
                      `N Bacteroides`=c(paste(table(et$case_status, et$ET)[2,1],' (',
                                              round(table(et$case_status, et$ET)[2,1]/
                                                    sum(table(et$case_status, et$ET)[2,])*100,0),
                                              '%)', sep=''),
                                        paste(table(et$case_status, et$ET)[1,1],' (',
                                              round(table(et$case_status, et$ET)[1,1]/
                                                    sum(table(et$case_status, et$ET)[1,])*100,0),
                                              '%)', sep='')),
                      `N Firmicutes`=c(paste(table(et$case_status, et$ET)[2,2],' (',
                                             round(table(et$case_status, et$ET)[2,2]/
                                                   sum(table(et$case_status, et$ET)[2,])*100,0),
                                             '%)', sep=''),
                                       paste(table(et$case_status, et$ET)[1,2],' (',
                                             round(table(et$case_status, et$ET)[1,2]/
                                                   sum(table(et$case_status, et$ET)[1,])*100,0),
                                             '%)', sep='')),
                      `N Prevotella`=c(paste(table(et$case_status, et$ET)[2,3],' (',
                                             round(table(et$case_status, et$ET)[2,3]/
                                                   sum(table(et$case_status, et$ET)[2,])*100,0),
                                             '%)', sep=''),
                                       paste(table(et$case_status, et$ET)[1,3],' (',
                                             round(table(et$case_status, et$ET)[1,3]/
                                                   sum(table(et$case_status, et$ET)[1,])*100,0),
                                             '%)', sep='')),
                      `PD vs NHC`=c('',
                                    paste('X2=',round(x2.test.res$statistic,1),
                                    ', P=',formatC(x2.test.res$p.value,format='e',digits=0),
                                    sep='')),
                      `PD observed vs expected`=c('',
                                                  paste('X2=',
                                                        round(x2.1,1),
                                                        ', P=',
                                                        formatC(x2.p1,format='e',digits=0),
                                                        sep='')),
                      `PD observed vs expected (no Firmicutes)`=c('',
                                                                  paste('X2=',
                                                                        round(x2.2,1),
                                                                        ', P=',
                                                                        round(x2.p2,2),
                                                                        sep='')),
```

```
                      `Odds ratio for Firmicutes`=c('',
                                                     paste('OR[95%CI]=',
                                                           round(fisher.ET_F$estimate,1),
                                                           '[',
                                                           round(fisher.ET_F$conf.int[1],1),
                                                           '-',
                                                           round(fisher.ET_F$conf.int[2],1),
                                                           ']',
                                                           ', P=',
                                                           formatC(fisher.ET_F$p.value,
                                                                   format='e',digits=0),
                                                           sep='')),
                      `Odds ratio for Prevotella`=c('',
                                                    paste('OR[95%CI]=',
                                                          round(fisher.ET_P$estimate,1),
                                                          '[',
                                                          round(fisher.ET_P$conf.int[1],1),
                                                          '-',
                                                          round(fisher.ET_P$conf.int[2],1),
                                                          ']',
                                                          ', P=',
                                                          round(fisher.ET_P$p.value,2),
                                                          sep='')),
                      `Odds ratio for Prevotella (no Firmicutes)`=c('',
                                                      paste('OR[95%CI]=',
                                                            round(fisher.ET_P2$estimate,1),
                                                            '[',
                                                            round(fisher.ET_P2$conf.int[1],1),
                                                            '-',
                                                            round(fisher.ET_P2$conf.int[2],1),']',
                                                            ', P=',
                                                            round(fisher.ET_P2$p.value,2),
                                                            sep='')),
                      check.names=FALSE)

# write results
# create workbook
wb <- createWorkbook()
# add worksheet, write data, and format output
addWorksheet(wb, 'Enterotype results')
writeData(wb, 'Enterotype results', results, keepNA=TRUE)
setColWidths(wb, 'Enterotype results', cols=seq_len(ncol(results)),
             widths=c(rep(15,8),rep(26,3))) ### format cells
addStyle(wb, 'Enterotype results', cols=seq_len(ncol(results)),
         rows=1:(nrow(results)+1), gridExpand=TRUE, style=center, stack=TRUE)
addStyle(wb, 'Enterotype results', cols=seq_len(ncol(results)),
         rows=1, style=bold, stack=TRUE) ### font
addStyle(wb, 'Enterotype results', cols=seq_len(ncol(results)),
         rows=c(1,2,(nrow(results)+2)), ### borders
         gridExpand=TRUE, style=horizontal_border_med, stack=TRUE)
# save workbook
saveWorkbook(wb,
    'PDShotgunAnalysis_out/2.Gut_microbiome_composition/Enterotype_results.xlsx',
    overwrite=TRUE)
```

```r

# prep data for mosaic plot
counts <- table(dplyr::recode(et$case_status, PD=1L, NHC=2L), et$ET)
rownames(counts) <- c("PD", "NHC")
colnames(counts) <- c("Bacteroides","Firmicutes","Prevotella")
dimnames(counts) <- list(Case_status=c(paste("PD (N=",results$`N Total`[1],")",sep=""),
                                       paste("NHC (N=",results$`N Total`[2],")",sep="")),
                         Enterotype=c("Bacteroides", "Firmicutes", "Prevotella"))
percents <- rbind(paste(round(table(et$case_status, et$ET)[2,]/
                              sum(table(et$case_status, et$ET)[2,])*100,0),'%',sep=''),
                  paste(round(table(et$case_status, et$ET)[1,]/
                              sum(table(et$case_status, et$ET)[1,])*100,0),'%',sep=''))
rownames(percents) <- c("PD", "NHC")
colnames(percents) <- c("Bacteroides","Firmicutes","Prevotella")
dimnames(percents) <- list(Case_status=c(paste("PD (N=",results$`N Total`[1],")",sep=""),
                                         paste("NHC (N=",results$`N Total`[2],")",sep="")),
                           Enterotype=c("Bacteroides", "Firmicutes", "Prevotella"))

# create mosaic plot
pdf('PDShotgunAnalysis_out/2.Gut_microbiome_composition/Enterotype_mosaic_plot.pdf',
    height=3, width=10)
mosaic(counts, main=NULL, highlighting='Enterotype',
       highlighting_fill=c('grey50','dodgerblue3','firebrick'),
       spacing=spacing_equal(sp=0.5), margins=c(2,2,2,7),
       labeling=labeling_border(varname=FALSE, rot_labels=0,
       just_labels=c('center','center','center','right')),
       keep_aspect_ratio=FALSE, pop=FALSE)
  labeling_cells(text=percents, gp_text=gpar(col='white'), margin=0)(counts)
trash <- dev.off()
```

Differential abundance of species and genera

MWAS

To determine what species and genera are differentially abundant between PD and NHC samples, differential abundance analysis was performed using two methods: 1) ANCOM-BC with count data [relative abundance with unknown estimation x total reads] and 2) linear regression with log2 transformed relative abundances (without unknown estimation) as implemented in MaAsLin2.

- To perform differential abundance analysis using ANCOM-BC with counts, counts were used as input for the `ancombc` function of the `ANCOMBC` R package. The ANCOM-BC `formula` included case status (PD vs NHC), collection method (swab vs OMNIgene GUT kit), and total sequence count (taken from `#nread` line of bowtie2 intermediate files produced by MetaPhlAn) standardized using the `scale` function in R with default parameters. All parameters were left as default except for the FDR adjustment which was made to be the Benjamini-Hochberg (`BH`) method, and the `zero_cut` which was made `0.95` to make the effective sample size for analysis 37 samples.
- To peform differential abundance analysis using linear regression with log2 transformed relative abundances, relative abundances from MetaPhlAn were divided by 100 to convert to proportions and used as input to the `Maaslin2` function. All parameters were left as default except for `min_prevalence` which was set to `0.05` to make the effective sample size 37, `normalization` which was set to `NONE` as we are already inputting relative abundances, `standardize` which was set to `FALSE` as this is being done prior to MaAsLin2, and `max_significance` which was set to `0.05`. The MaAsLin2 `fixed_effects` model included case status (PD vs NHC), collection method (swab vs OMNIgene GUT kit), and total sequence

count (taken from `#nread` line of bowtie2 intermediate files produced by MetaPhlAn) standardized using the `scale` function.

```
#### SPECIES AND GENUS MWAS ####

# recode categorical variables to get correct effect direction and scale numeric data
sample_data(abun.ps.s)$Case_status <- dplyr::recode(sample_data(abun.ps.s)$Case_status,
                                                    PD=1, Control=0)
sample_data(abun.ps.g)$Case_status <- dplyr::recode(sample_data(abun.ps.g)$Case_status,
                                                    PD=1, Control=0)
sample_data(abun.ps.s)$collection_method <- dplyr::recode(sample_data(abun.ps.s)$collection_method,
                                                          swab=1, `OMNIgene GUT`=0)
sample_data(abun.ps.g)$collection_method <- dplyr::recode(sample_data(abun.ps.g)$collection_method,
                                                          swab=1, `OMNIgene GUT`=0)
sample_data(abun.ps.s)$seqs_scaled <- scale(sample_data(abun.ps.s)$total_sequences)
sample_data(abun.ps.g)$seqs_scaled <- scale(sample_data(abun.ps.g)$total_sequences)

sample_data(ra.ps.s)$Case_status <- dplyr::recode(sample_data(ra.ps.s)$Case_status,
                                                  PD=1, Control=0)
sample_data(ra.ps.g)$Case_status <- dplyr::recode(sample_data(ra.ps.g)$Case_status,
                                                  PD=1, Control=0)
sample_data(ra.ps.s)$collection_method <- dplyr::recode(sample_data(ra.ps.s)$collection_method,
                                                        swab=1, `OMNIgene GUT`=0)
sample_data(ra.ps.g)$collection_method <- dplyr::recode(sample_data(ra.ps.g)$collection_method,
                                                        swab=1, `OMNIgene GUT`=0)
sample_data(ra.ps.s)$seqs_scaled <- scale(sample_data(ra.ps.s)$total_sequences)
sample_data(ra.ps.g)$seqs_scaled <- scale(sample_data(ra.ps.g)$total_sequences)

# perform differential abundance analysis using ANCOM-BC with count data
ancom.s <- ANCOMBC.plus(ps=abun.ps.s,
                        formula="Case_status + collection_method + seqs_scaled",
                        p_adj_method="BH",
                        zero_cut=0.95)
ancom.g <- ANCOMBC.plus(ps=abun.ps.g,
                        formula="Case_status + collection_method + seqs_scaled",
                        p_adj_method="BH",
                        zero_cut=0.95)

# prep temporary directory for MaAsLin2 output
system('
if [ ! -d "temp_directory" ]
then
  mkdir temp_directory
fi
')

# perform differential abundance analysis using linear regression with
# log2 transformed relative abundances
suppress(
lm.s <- MaAsLin2.plus(ps=phyloseq(otu_table(ra.ps.s)/100,
                      sample_data(ra.ps.s)),
                      output='temp_directory',
                      metadata=c('Case_status','collection_method','seqs_scaled'),
                      min_prevalence=0.05,
```

```
                      normalization='NONE',
                      max_significance=0.05,
                      standardize=FALSE,
                      plot_heatmap=FALSE,
                      plot_scatter=FALSE)
)
suppress(
lm.g <- MaAsLin2.plus(ps=phyloseq(otu_table(ra.ps.g)/100,
                      sample_data(ra.ps.g)),
                      output='temp_directory',
                      metadata=c('Case_status','collection_method','seqs_scaled'),
                      min_prevalence=0.05,
                      normalization='NONE',
                      max_significance=0.05,
                      standardize=FALSE,
                      plot_heatmap=FALSE,
                      plot_scatter=FALSE)
)

# remove temporary output directory
system('rm -r temp_directory')

# initialize workbook
wb <- createWorkbook()

# coalesce results for species
res.summ <- merge(
            data.frame(Variable=lm.s$result.summary$Variable,
                       Kingdom=gsub('_', ' ',
                                    gsub('k__', '',
                                         sapply(strsplit(lm.s$result.summary$Feature, "\\|"),
                                                function(x){x[1]}))),
                       Phylum=gsub('_', ' ',
                                   gsub('p__', '',
                                        sapply(strsplit(lm.s$result.summary$Feature, "\\|"),
                                               function(x){x[2]}))),
                       Class=gsub('_', ' ',
                                  gsub('c__', '',
                                       sapply(strsplit(lm.s$result.summary$Feature, "\\|"),
                                              function(x){x[3]}))),
                       Order=gsub('_', ' ',
                                  gsub('o__', '',
                                       sapply(strsplit(lm.s$result.summary$Feature, "\\|"),
                                              function(x){x[4]}))),
                       Family=gsub('_', ' ',
                                   gsub('f__', '',
                                        sapply(strsplit(lm.s$result.summary$Feature, "\\|"),
                                               function(x){x[5]}))),
                       Genus=gsub('_', ' ',
                                  gsub('g__', '',
                                       sapply(strsplit(lm.s$result.summary$Feature, "\\|"),
                                              function(x){x[6]}))),
                       Species=gsub('_', ' ',
                                    gsub('s__', '',
```

```
                                         sapply(strsplit(lm.s$result.summary$Feature, "\\|"),
                                                function(x){x[7]})),
                       `N PD`=lm.s$result.summary$N1,
                       `N NHC`=lm.s$result.summary$N2,
                       space_1='',
                       `RA in PD`=lm.s$result.summary$Mean1,
                       `RA in NHC`=lm.s$result.summary$Mean2,
                       lm.s$result.summary[,c('Beta','SE','P','FDR','FC')],
                       `FC lower`=lm.s$result.summary$FC_lower,
                       `FC upper`=lm.s$result.summary$FC_upper,
                       space_2='', check.names=FALSE),
            data.frame(Variable=ancom.s$result.summary$Variable,
                       Kingdom=gsub('_', ' ',
                                    gsub('k__', '',
                                         sapply(strsplit(ancom.s$result.summary$Feature, "\\|"),
                                                function(x){x[1]}))),
                        Phylum=gsub('_', ' ',
                                    gsub('p__', '',
                                         sapply(strsplit(ancom.s$result.summary$Feature, "\\|"),
                                                function(x){x[2]}))),
                        Class=gsub('_', ' ',
                                   gsub('c__', '',
                                        sapply(strsplit(ancom.s$result.summary$Feature, "\\|"),
                                               function(x){x[3]}))),
                        Order=gsub('_', ' ',
                                   gsub('o__', '',
                                        sapply(strsplit(ancom.s$result.summary$Feature, "\\|"),
                                               function(x){x[4]}))),
                        Family=gsub('_', ' ',
                                    gsub('f__', '',
                                         sapply(strsplit(ancom.s$result.summary$Feature, "\\|"),
                                                function(x){x[5]}))),
                        Genus=gsub('_', ' ',
                                   gsub('g__', '',
                                        sapply(strsplit(ancom.s$result.summary$Feature, "\\|"),
                                               function(x){x[6]}))),
                        Species=gsub('_', ' ',
                                     gsub('s__', '',
                                          sapply(strsplit(ancom.s$result.summary$Feature, "\\|"),
                                                 function(x){x[7]}))),
                        `N PD`=ancom.s$result.summary$N1,
                        `N NHC`=ancom.s$result.summary$N2,
                        `BC-OA in PD`=ancom.s$result.summary$Mean1,
                        `BC-OA in NHC`=ancom.s$result.summary$Mean2,
                        ancom.s$result.summary[,c('Beta','SE','P','FDR','FC')],
                        `FC lower`=ancom.s$result.summary$FC_lower,
                        `FC upper`=ancom.s$result.summary$FC_upper,
                        check.names=FALSE),
                  by=c('Variable','Kingdom','Phylum','Class','Order',
                       'Family','Genus','Species','N PD','N NHC'),
                  suffix=c('_m','_a'), all=TRUE, sort=FALSE)
res.summ <- res.summ[res.summ$Variable=='Case_status',-1]
res.summ <- rbind(data.frame(Kingdom='', Phylum='', Class='', Order='',
                             Family='', Genus='', Species='',
```

```
                             `N PD`='', `N NHC`='', space_1='',
                             `RA in PD`='MaAsLin2 results', `RA in NHC`='',
                             Beta_m='', SE_m='', P_m='', FDR_m='',
                             FC_m='', `FC lower_m`='', `FC upper_m`='', space_2='',
                             `BC-OA in PD`='ANCOM-BC results', `BC-OA in NHC`='',
                             Beta_a='', SE_a='', P_a='', FDR_a='',
                             FC_a='', `FC lower_a`='', `FC upper_a`='',
                             check.names=FALSE),
                  data.frame(Kingdom='Kingdom', Phylum='Phylum', Class='Class', Order='Order',
                             Family='Famiy', Genus='Genus', Species='Species',
                             `N PD`='N PD', `N NHC`='N NHC', space_1='',
                             `RA in PD`='RA in PD', `RA in NHC`='RA in NHC',
                             Beta_m='Beta', SE_m='SE', P_m='P', FDR_m='FDR', FC_m='FC',
                             `FC lower_m`='FC lower', `FC upper_m`='FC upper', space_2='',
                             `BC-OA in PD`='BC-OA in PD', `BC-OA in NHC`='BC-OA in NHC',
                             Beta_a='Beta', SE_a='SE', P_a='P', FDR_a='FDR', FC_a='FC',
                             `FC lower_a`='FC lower', `FC upper_a`='FC upper',
                             check.names=FALSE),
                  res.summ)
res.summ[3:(nrow(res.summ)-1),
         c(grep('MaAsLin2', res.summ[1,]):(grep('space_2', colnames(res.summ))-1),
           grep('ANCOM', res.summ[1,]):ncol(res.summ))][
         is.na(res.summ[3:(nrow(res.summ)-1),
                        c(grep('MaAsLin2', res.summ[1,]):(grep('space_2', colnames(res.summ))-1),
                          grep('ANCOM', res.summ[1,]):ncol(res.summ))])] <- 'NT'

# add species results to workbook and format
addWorksheet(wb, 'Species results')
writeData(wb, 'Species results', res.summ, keepNA=FALSE, colNames=FALSE)
setColWidths(wb, 'Species results', cols=seq_len(ncol(res.summ)),
             widths=c(10, rep(22,6), rep(11,2), 2, rep(11,9), 2, rep(11,9))) ### format cells
mergeCells(wb, 'Species results',
           cols=grep('MaAsLin2', res.summ[1,]):(grep('space_2', colnames(res.summ))-1), rows=1)
mergeCells(wb, 'Species results',
           cols=grep('ANCOM', res.summ[1,]):ncol(res.summ), rows=1)
addStyle(wb, 'Species results',
         cols=seq_len(ncol(res.summ)), rows=1:2, style=bold, stack=TRUE, gridExpand=TRUE) ### font
addStyle(wb, 'Species results',
         cols=seq_len(ncol(res.summ)), rows=c(1,3,(nrow(res.summ)+1)), ### borders
         gridExpand=TRUE, style=horizontal_border_med, stack=TRUE)
addStyle(wb, 'Species results',
         cols=grep('MaAsLin2', res.summ[1,]):(grep('space_2', colnames(res.summ))-1), rows=2,
         style=horizontal_border_thin, stack=TRUE)
addStyle(wb, 'Species results', cols=grep('ANCOM', res.summ[1,]):ncol(res.summ),
         rows=2, style=horizontal_border_thin, stack=TRUE)
# convert numbers from strings back to numbers
convertNum(res.summ, wb, 'Species results', FALSE)

# coalesce results for genera
res.summ <- merge(
            data.frame(Variable=lm.g$result.summary$Variable,
                       Kingdom=gsub('_', ' ',
                                    gsub('k__', '',
                                         sapply(strsplit(lm.g$result.summary$Feature, "\\|"),
```

```
                                            function(x){x[1]}))),
                   Phylum=gsub('_', ' ',
                               gsub('p__', '',
                                    sapply(strsplit(lm.g$result.summary$Feature, "\\|"),
                                           function(x){x[2]}))),
                   Class=gsub('_', ' ',
                              gsub('c__', '',
                                   sapply(strsplit(lm.g$result.summary$Feature, "\\|"),
                                          function(x){x[3]}))),
                   Order=gsub('_', ' ',
                              gsub('o__', '',
                                   sapply(strsplit(lm.g$result.summary$Feature, "\\|"),
                                          function(x){x[4]}))),
                   Family=gsub('_', ' ',
                               gsub('f__', '',
                                    sapply(strsplit(lm.g$result.summary$Feature, "\\|"),
                                           function(x){x[5]}))),
                   Genus=gsub('_', ' ',
                              gsub('g__', '',
                                   sapply(strsplit(lm.g$result.summary$Feature, "\\|"),
                                          function(x){x[6]}))),
                   `N PD`=lm.g$result.summary$N1,
                   `N NHC`=lm.g$result.summary$N2,
                   space_1='',
                   `RA in PD`=lm.g$result.summary$Mean1,
                   `RA in NHC`=lm.g$result.summary$Mean2,
                   lm.g$result.summary[,c('Beta','SE','P','FDR','FC')],
                   `FC lower`=lm.g$result.summary$FC_lower,
                   `FC upper`=lm.g$result.summary$FC_upper,
                   space_2='', check.names=FALSE),
        data.frame(Variable=ancom.g$result.summary$Variable,
                   Kingdom=gsub('_', ' ',
                                gsub('k__', '',
                                     sapply(strsplit(ancom.g$result.summary$Feature, "\\|"),
                                            function(x){x[1]}))),
                    Phylum=gsub('_', ' ',
                                gsub('p__', '',
                                     sapply(strsplit(ancom.g$result.summary$Feature, "\\|"),
                                            function(x){x[2]}))),
                    Class=gsub('_', ' ',
                               gsub('c__', '',
                                    sapply(strsplit(ancom.g$result.summary$Feature, "\\|"),
                                           function(x){x[3]}))),
                    Order=gsub('_', ' ',
                               gsub('o__', '',
                                    sapply(strsplit(ancom.g$result.summary$Feature, "\\|"),
                                           function(x){x[4]}))),
                    Family=gsub('_', ' ',
                                gsub('f__', '',
                                     sapply(strsplit(ancom.g$result.summary$Feature, "\\|"),
                                            function(x){x[5]}))),
                    Genus=gsub('_', ' ',
                               gsub('g__', '',
                                    sapply(strsplit(ancom.g$result.summary$Feature, "\\|"),
```

```r
                                               function(x){x[6]})),
                        `N PD`=ancom.g$result.summary$N1,
                        `N NHC`=ancom.g$result.summary$N2,
                        `BC-OA in PD`=ancom.g$result.summary$Mean1,
                        `BC-OA in NHC`=ancom.g$result.summary$Mean2,
                        ancom.g$result.summary[,c('Beta','SE','P','FDR','FC')],
                        `FC lower`=ancom.g$result.summary$FC_lower,
                        `FC upper`=ancom.g$result.summary$FC_upper,
                        check.names=FALSE),
                  by=c('Variable','Kingdom','Phylum','Class','Order',
                       'Family','Genus','N PD','N NHC'),
                  suffix=c('_m','_a'), all=TRUE, sort=FALSE)
res.summ <- res.summ[res.summ$Variable=='Case_status',-1]
res.summ <- rbind(data.frame(Kingdom='', Phylum='', Class='', Order='', Family='', Genus='',
                             `N PD`='', `N NHC`='', space_1='',
                             `RA in PD`='MaAsLin2 results', `RA in NHC`='',
                             Beta_m='', SE_m='', P_m='', FDR_m='',
                             FC_m='', `FC lower_m`='', `FC upper_m`='', space_2='',
                             `BC-OA in PD`='ANCOM-BC results', `BC-OA in NHC`='',
                             Beta_a='', SE_a='', P_a='', FDR_a='',
                             FC_a='', `FC lower_a`='', `FC upper_a`='',
                             check.names=FALSE),
                  data.frame(Kingdom='Kingdom', Phylum='Phylum', Class='Class',
                             Order='Order', Family='Famiy', Genus='Genus',
                             `N PD`='N PD', `N NHC`='N NHC', space_1='',
                             `RA in PD`='RA in PD', `RA in NHC`='RA in NHC',
                             Beta_m='Beta', SE_m='SE', P_m='P', FDR_m='FDR', FC_m='FC',
                             `FC lower_m`='FC lower', `FC upper_m`='FC upper', space_2='',
                             `BC-OA in PD`='BC-OA in PD', `BC-OA in NHC`='BC-OA in NHC',
                             Beta_a='Beta', SE_a='SE', P_a='P', FDR_a='FDR', FC_a='FC',
                             `FC lower_a`='FC lower', `FC upper_a`='FC upper',
                             check.names=FALSE),
                  res.summ)
res.summ[3:(nrow(res.summ)-1),
         c(grep('MaAsLin2', res.summ[1,]):(grep('space_2', colnames(res.summ))-1),
           grep('ANCOM', res.summ[1,]):ncol(res.summ))][
         is.na(res.summ[3:(nrow(res.summ)-1),
                        c(grep('MaAsLin2', res.summ[1,]):(grep('space_2', colnames(res.summ))-1),
                          grep('ANCOM', res.summ[1,]):ncol(res.summ))])] <- 'NT'

# add genus results to workbook and format
addWorksheet(wb, 'Genus results')
writeData(wb, 'Genus results', res.summ, keepNA=FALSE, colNames=FALSE)
setColWidths(wb, 'Genus results', cols=seq_len(ncol(res.summ)),
             widths=c(10, rep(22,5), rep(11,2), 2, rep(11,9), 2, rep(11,9))) ### format cells
mergeCells(wb, 'Genus results',
           cols=grep('MaAsLin2', res.summ[1,]):(grep('space_2', colnames(res.summ))-1), rows=1)
mergeCells(wb, 'Genus results',
           cols=grep('ANCOM', res.summ[1,]):ncol(res.summ), rows=1)
addStyle(wb, 'Genus results',
         cols=seq_len(ncol(res.summ)), rows=1:2, style=bold, stack=TRUE, gridExpand=TRUE) ### font
addStyle(wb, 'Genus results',
         cols=seq_len(ncol(res.summ)), rows=c(1,3,(nrow(res.summ)+1)), ### borders
         gridExpand=TRUE, style=horizontal_border_med, stack=TRUE)
```

```
addStyle(wb, 'Genus results',
         cols=grep('MaAsLin2', res.summ[1,]):(grep('space_2', colnames(res.summ))-1), rows=2,
         style=horizontal_border_thin, stack=TRUE)
addStyle(wb, 'Genus results',
         cols=grep('ANCOM', res.summ[1,]):ncol(res.summ),
         rows=2, style=horizontal_border_thin, stack=TRUE)
# convert numbers from strings back to numbers
convertNum(res.summ, wb, 'Genus results', FALSE)

# save workbook
saveWorkbook(wb,
    'PDShotgunAnalysis_out/3.Taxonomic_associations/MaAsLin2_ANCOMBC_MWAS_PDvsNHC.xlsx',
    overwrite=TRUE)
```

MaAsLin2 and ANCOM-BC MWAS concordance

To visualize the concordance of results between MaAsLin2 and ANCOM-BC, the FDR q-values resulting from each method were plotted together. Species tagged as significantly enriched (colored blue) or depleted (colored red) in PD were also highlighted. Venn diagrams showing the overlap of detected signals at FDR < 0.05 and < 0.1 between MaAsLin2 and ANCOM-BC were also generated.

```
#### MAASLIN2 AND ANCOMBC MWAS CONCORDANCE ####

# get FDR q-values ready for plotting
plot.data <- merge(lm.s$result.summary[lm.s$result.summary$Variable == 'Case_status',
                                       c('Feature','FDR','FC')],
                   ancom.s$result.summary[ancom.s$result.summary$Variable == 'Case_status',
                                          c('Feature','FDR','FC')],
                   by='Feature', suffix=c('_maaslin','_ancombc'))
plot.data <- plot.data[rowSums(is.na(plot.data)) == 0,]

# tag PD-associated enriched and depleted species
plot.data$`PD association` <- ifelse(plot.data[,2] < 0.05 & round(plot.data[,4],1) <= 0.1 |
                                     round(plot.data[,2],1) <= 0.1 & plot.data[,4] < 0.05,
                                     ifelse(plot.data[,3] > 1 & plot.data[,5] > 1, 'enriched',
                                            ifelse(plot.data[,3] < 1 & plot.data[,5] < 1,
                                                   'depleted', 'opposite directions')),
                                     'not associated')

# create column to label features reaching FDR 1E-4 in either method
labels <- gsub('_',' ',sapply(plot.data[,1], function(x){strsplit(x, 's__')[[1]][2]}))
plot.data$labels <- ''
plot.data$labels[plot.data[,2] < 1E-4 | plot.data[,4] < 1E-4] <- labels[plot.data[,2] < 1E-4 |
                                                                         plot.data[,4] < 1E-4]

# tag what species were detected at FDR q-value thresholds of 0.1 and 0.05
plot.data$`MaAsLin2 FDR<0.1`[plot.data$FDR_maaslin < 0.1] <- TRUE
plot.data$`MaAsLin2 FDR<0.1`[plot.data$FDR_maaslin > 0.1] <- FALSE
plot.data$`MaAsLin2 FDR<0.05`[plot.data$FDR_maaslin < 0.05] <- TRUE
plot.data$`MaAsLin2 FDR<0.05`[plot.data$FDR_maaslin > 0.05] <- FALSE

plot.data$`ANCOM-BC FDR<0.1`[plot.data$FDR_ancombc < 0.1] <- TRUE
plot.data$`ANCOM-BC FDR<0.1`[plot.data$FDR_ancombc > 0.1] <- FALSE
plot.data$`ANCOM-BC FDR<0.05`[plot.data$FDR_ancombc < 0.05] <- TRUE
```

```
plot.data$`ANCOM-BC FDR<0.05`[plot.data$FDR_ancombc > 0.05] <- FALSE

plot.data$`MaAsLin2 FDR<0.1`[plot.data$`MaAsLin2 FDR<0.1` +
                             plot.data$`ANCOM-BC FDR<0.1` == 0] <- NA
plot.data$`ANCOM-BC FDR<0.1`[plot.data$`MaAsLin2 FDR<0.1` +
                             plot.data$`ANCOM-BC FDR<0.1` == 0] <- NA
plot.data$`MaAsLin2 FDR<0.05`[plot.data$`MaAsLin2 FDR<0.05` +
                              plot.data$`ANCOM-BC FDR<0.05` == 0] <- NA
plot.data$`ANCOM-BC FDR<0.05`[plot.data$`MaAsLin2 FDR<0.05` +
                              plot.data$`ANCOM-BC FDR<0.05` == 0] <- NA

# create venn diagrams of overlapping signals
g1 <- ggplot(data=plot.data) +
  geom_venn(aes(A=`MaAsLin2 FDR<0.1`, B=`ANCOM-BC FDR<0.1`), fill_color='white',
            stroke_size=0.5, stroke_color='black', stroke_linetype=c('dashed','solid'),
            set_name_size=4, text_size=7, auto_scale=TRUE, position=position_dodge(2),
            show_percentage=FALSE) +
  theme_void()
ggsave(
'PDShotgunAnalysis_out/3.Taxonomic_associations/MaAsLin2_vs_ANCOMBC_species_venn_diag_FDR_0.1.pdf',
g1, device='pdf', width=5, height=5)

g2 <- ggplot(data=plot.data) +
  geom_venn(aes(A=`MaAsLin2 FDR<0.05`, B=`ANCOM-BC FDR<0.05`), fill_color='white',
            stroke_size=0.5, stroke_color='black', stroke_linetype=c('dashed','solid'),
            set_name_size=4, text_size=7, auto_scale=TRUE, position=position_dodge(2),
            show_percentage=FALSE) +
  theme_void()
ggsave(
'PDShotgunAnalysis_out/3.Taxonomic_associations/MaAsLin2_vs_ANCOMBC_species_venn_diag_FDR_0.05.pdf',
g2, device='pdf', width=5, height=5)

# create scatter plot of FDR q-values
set.seed(1234)
g3 <- ggplot(data=plot.data, aes(y=-log10(plot.data[,2]), x=-log10(plot.data[,4]),
             fill=`PD association`, label=labels)) +
  geom_point(size=4, shape=21) +
  geom_text_repel(min.segment.length=0, box.padding=0.5, size=6, color='grey25') +
  geom_vline(xintercept=-log10(0.05), color='grey50', linetype='dashed') +
  geom_hline(yintercept=-log10(0.05), color='grey50', linetype='dashed') +
  labs(y='-log10(MaAsLin2 FDR)', x='-log10(ANCOM-BC FDR)') +
  scale_y_continuous(breaks=c(0,-log10(0.05),-log10(0.01),-log10(1E-4),-log10(1E-6),-log10(1E-8)),
                     labels=c('0',
                              paste(round(-log10(0.05),1),' \n','(0.05)',sep=''),
                              paste(-log10(0.01),' \n','(0.01)',sep=''),
                              paste(-log10(1E-4),' \n','(1E-4)',sep=''),
                              paste(-log10(1E-6),' \n','(1E-6)',sep=''),
                              paste(-log10(1E-8),' \n','(1E-8)',sep='')),
                     limits=c(0,-log10(min(plot.data[,c('FDR_maaslin','FDR_ancombc')]))),
                     minor_breaks=NULL) +
  scale_x_continuous(breaks=c(0,-log10(0.05),-log10(0.01),-log10(1E-4),-log10(1E-6),-log10(1E-8)),
                     labels=c('0',
                              paste(round(-log10(0.05),1),' \n','(0.05)',sep=''),
                              paste(-log10(0.01),'\n','(0.01)',sep=''),
```

```
                              paste(-log10(1E-4),'\n','(1E-4)',sep=''),
                              paste(-log10(1E-6),'\n','(1E-6)',sep=''),
                              paste(-log10(1E-8),'\n','(1E-8)',sep='')),
                     limits=c(0,-log10(min(plot.data[,c('FDR_maaslin','FDR_ancombc')]))),
                     minor_breaks=NULL) +
  scale_fill_manual(values=c('red','blue','grey')) +
  guides(fill=guide_legend(override.aes=list(shape=21))) +
  theme_bw() +
  theme(legend.title=element_text(size=20), legend.text=element_text(size=20), legend.text.align=0,
        legend.position=c(0.87, 0.3), legend.background=element_rect(fill='white', color='grey50'),
        axis.text.x=element_text(size=18), axis.text.y=element_text(size=18, vjust=0.8),
        axis.title=element_text(size=20),
        axis.title.x=element_text(vjust=-0.75), axis.title.y=element_text(vjust=3),
        plot.margin=margin(t=10, r=30, b=10, l=10, unit = "pt"))
ggsave(
'PDShotgunAnalysis_out/3.Taxonomic_associations/MaAsLin2_vs_ANCOMBC_species_MWAS_qvalues.pdf',
g3, device='pdf', width=12, height=10)
```

Genus heterogeneity

Species counts for species that were tested, found significant, and found elevated or reduced for each significant genus from differential abundance analysis were calculated to observe heterogeneity of genera and their association with PD. This was also done for genera not found significant in differential abunance analysis, but had a significant species in the species differential abundance analysis.

```
#### GENUS HETEROGENEITY ####

# get names of significant taxa and tested taxa
sub.data <- merge(lm.s$result.summary[lm.s$result.summary$Variable == 'Case_status',
                                      c('Feature','FDR')],
                  ancom.s$result.summary[ancom.s$result.summary$Variable == 'Case_status',
                                          c('Feature','FDR')],
                  by='Feature')
tested.species <- sub.data$Feature[rowSums(is.na(sub.data)) == 0]
sig.species <- ifelse(sub.data[,2] < 0.05 & round(sub.data[,3],1) <= 0.1 |
                      round(sub.data[,2],1) <= 0.1 & sub.data[,3] < 0.05,
                      sub.data$Feature, NA)
sig.species <- sig.species[!is.na(sig.species)]

sub.data <- merge(lm.g$result.summary[lm.g$result.summary$Variable == 'Case_status',
                                      c('Feature','FDR')],
                  ancom.g$result.summary[ancom.g$result.summary$Variable == 'Case_status',
                                          c('Feature','FDR')],
                  by='Feature')
tested.genera <- sub.data$Feature[rowSums(is.na(sub.data)) == 0]
sig.genera <- ifelse(sub.data[,2] < 0.05 & round(sub.data[,3],1) <= 0.1 |
                     round(sub.data[,2],1) <= 0.1 & sub.data[,3] < 0.05,
                     sub.data$Feature, NA)
sig.genera <- sig.genera[!is.na(sig.genera)]

# create table giving species count for each significant genus
species.counts <- data.frame()
for (genus in seq_along(tested.genera)){
  genus.name <- strsplit(tested.genera[genus], '\\|')[[1]][6]
```

```r
  species.n <- length(tested.species[grep(genus.name, tested.species)])
  elev.species <- length(
                  lm.s$result.summary$Feature[lm.s$result.summary$Variable == 'Case_status' &
                                              lm.s$result.summary$Feature %in%
                                              sig.species[grep(genus.name, sig.species)] &
                                              lm.s$result.summary$FC > 1])
  red.species <- length(
                 lm.s$result.summary$Feature[lm.s$result.summary$Variable == 'Case_status' &
                                             lm.s$result.summary$Feature %in%
                                             sig.species[grep(genus.name, sig.species)] &
                                             lm.s$result.summary$FC < 1])
  if (tested.genera[genus] %in% sig.genera){
    genus.fc <- ifelse(lm.g$result.summary$FC[lm.g$result.summary$Variable == 'Case_status' &
                                        lm.g$result.summary$Feature == tested.genera[genus]] < 1,
                                        'Reduced', 'Elevated')
  }else{
    genus.fc <- 'Missed'
  }
  species.counts <- rbind(species.counts,
                          data.frame(`PD-associated genera`=gsub('_', ' ',
                                                                 gsub('g__', '', genus.name)),
                                     `N species tested`=species.n,
                                     `N PD assoc species`=elev.species+red.species,
                                     `N species elevated`=elev.species,
                                     `N species reduced`=red.species,
                                     `Genus level MWAS`=genus.fc,
                                     check.names=FALSE))
}

# remove genera who were not significant and did not have any significant species
species.counts <- species.counts[species.counts$`N PD assoc species` > 0 |
                                 species.counts$`Genus level MWAS` != 'Missed',]

# sort by genus name and put missed genera at bottom
species.counts <- species.counts[order(species.counts$`PD-associated genera`),]
species.counts <- rbind(species.counts[species.counts$`Genus level MWAS` != 'Missed',],
        data.frame(`PD-associated genera`='Association at species level, missed at genus level',
                   `N species tested`='',
                   `N PD assoc species`='',
                   `N species elevated`='',
                   `N species reduced`='',
                   `Genus level MWAS`='',
                    check.names=FALSE),
            species.counts[species.counts$`Genus level MWAS` == 'Missed',])

# write results
# create workbook
wb <- createWorkbook()
# add worksheet, write data, and format output
addWorksheet(wb, 'Genus hetero')
writeData(wb, 'Genus hetero', species.counts, keepNA=TRUE, colNames=TRUE)
setColWidths(wb, 'Genus hetero', cols=seq_len(ncol(species.counts)),
             widths=c(29, rep(18, (ncol(species.counts)-1)))) ### format cells
mergeCells(wb, 'Genus hetero', cols=seq_len(ncol(species.counts)), rows=36)
```

```
addStyle(wb, 'Genus hetero', cols=seq_len(ncol(species.counts)),
         rows=c(1,36), style=bold, gridExpand=TRUE, stack=TRUE) ### font
addStyle(wb, 'Genus hetero', cols=seq_len(ncol(species.counts)),
         rows=c(1,2,36,37,(nrow(species.counts)+2)),  ### borders
         style=horizontal_border_med, gridExpand=TRUE, stack=TRUE)
# convert numbers from strings back to numbers
convertNum(species.counts, wb, 'Genus hetero', TRUE)
# save workbook
saveWorkbook(wb,
    'PDShotgunAnalysis_out/3.Taxonomic_associations/Genus_heterogeneity.xlsx',
    overwrite=TRUE)
```

Species distributions and fold changes from MWAS

Log2 transformed relative abundances and natural log transformed bias-corrected abundances (estimated from ANCOM-BC) were plotted as boxplots for the 84 PD-associated species to see distribution of the data, along with fold changes from MaAsLin2 and ANCOM-BC. A smaller plot was also created focusing on PD-associated species that had a 75% change in relative abundance (absolute fold change of 1.75 or higher).

```
#### PD-ASSOCIATED SPECIES DISTRIBUTION & FOLD CHANGES ####

# grab relative abundances and bias-corrected abundances of species
ra.spp <- data.frame(otu_table(ra.ps.s)/100, check.names=FALSE)
ba.spp <- data.frame(otu_table(ancom.s$bias.corrected.ps), check.names=FALSE)

# pull out plotting data for PD-associated species
spp.ra.data <- ra.spp[,colnames(ra.spp) %in% sig.species, FALSE]
spp.ra.fc.data <- lm.s$result.summary[lm.s$result.summary$Feature %in% sig.species &
                                      lm.s$result.summary$Variable == 'Case_status',
                                      c('FC','FC_lower','FC_upper')]
spp.ra.fc.data <- spp.ra.fc.data[order(sapply(spp.ra.fc.data$FC,
                                              function(x){ifelse(x<1,1/x,x)}),
                                       decreasing=FALSE),]
spp.ra.data <- spp.ra.data[,rownames(spp.ra.fc.data), FALSE]
spp.ra.fc.data <- data.frame(plot='Absolute fold change with 95%CI', line='MaAsLin2',
                             variable=gsub('_',' ',
                                           sapply(strsplit(rownames(spp.ra.fc.data),
                                                           "\\|s__"),
                                                  function(x){x[2]})),
                             spp.ra.fc.data)
spp.ra.data <- data.frame(plot='log2(Relative abundances)',
                          Case_status=sample_data(ra.ps.s)$Case_status,
                          spp.ra.data, check.names=FALSE)

spp.ba.data <- ba.spp[,colnames(ba.spp) %in% sig.species, FALSE]
spp.ba.fc.data <- ancom.s$result.summary[ancom.s$result.summary$Feature %in% sig.species &
                                         ancom.s$result.summary$Variable == 'Case_status',
                                         c('FC','FC_lower','FC_upper')]
spp.ba.fc.data <- spp.ba.fc.data[rownames(spp.ra.fc.data),, FALSE]
spp.ba.data <- spp.ba.data[,rownames(spp.ra.fc.data), FALSE]
spp.ba.fc.data <- data.frame(plot='Absolute fold change with 95%CI', line='ANCOM-BC',
                             variable=gsub('_',' ',
                                           sapply(strsplit(rownames(spp.ba.fc.data),
                                                           "\\|s__"),
```

```r
                                                  function(x){x[2]})),
                             spp.ba.fc.data)
spp.ba.data <- data.frame(plot='log(Bias-corrected abundances)',
                          Case_status=sample_data(ra.ps.s)$Case_status,
                          spp.ba.data, check.names=FALSE)

# combine MaAsLin2 and ANCOM-BC data
fc.plot.data <- data.frame(rbind(spp.ra.fc.data, spp.ba.fc.data))
ab.plot.data <- merge(spp.ra.data, spp.ba.data, all=TRUE, sort=FALSE)
colnames(ab.plot.data)[3:ncol(ab.plot.data)] <- gsub('_',' ',
                                  sapply(strsplit(colnames(ab.plot.data)[3:ncol(ab.plot.data)],
                                                  "\\|s__"),
                                         function(x){x[2]}))

# prep fold change data for plotting
fc.plot.data$line <- factor(fc.plot.data$line, levels=rev(unique(fc.plot.data$line)))
fc.plot.data$variable <- factor(fc.plot.data$variable, levels=unique(fc.plot.data$variable))
fc.plot.data$color[fc.plot.data$FC < 1] <- 'elevated'
fc.plot.data$color[fc.plot.data$FC > 1] <- 'depleted'
fc.plot.data$FC_mod[fc.plot.data$FC > 1] <- fc.plot.data$FC[fc.plot.data$FC > 1]-1
fc.plot.data$FC_mod[fc.plot.data$FC < 1] <- -((1/fc.plot.data$FC[fc.plot.data$FC < 1])-1)
fc.plot.data$FC_lower_mod[fc.plot.data$FC_lower > 1] <-
          fc.plot.data$FC_lower[fc.plot.data$FC_lower > 1]-1
fc.plot.data$FC_lower_mod[fc.plot.data$FC_lower < 1] <-
          -((1/fc.plot.data$FC_lower[fc.plot.data$FC_lower < 1])-1)
fc.plot.data$FC_upper_mod[fc.plot.data$FC_upper > 1] <-
          fc.plot.data$FC_upper[fc.plot.data$FC_upper > 1]-1
fc.plot.data$FC_upper_mod[fc.plot.data$FC_upper < 1] <-
          -((1/fc.plot.data$FC_upper[fc.plot.data$FC_upper < 1])-1)

# prep abundance data for plotting
ab.plot.data$Case_status <- dplyr::recode(ab.plot.data$Case_status, '1'='PD', '0'='NHC')
ab.plot.data$Case_status <- factor(ab.plot.data$Case_status,
                                   levels=rev(unique(ab.plot.data$Case_status)))
ab.plot.data$plot <- factor(ab.plot.data$plot, levels=unique(ab.plot.data$plot))
ab.plot.data.melt <- reshape2::melt(ab.plot.data)
ab.plot.data.melt <- ab.plot.data.melt[!is.na(ab.plot.data.melt$value),]
ab.plot.data.melt$value[ab.plot.data.melt$plot == 'log2(Relative abundances)'] <-
      log2.trans(ab.plot.data.melt$value[ab.plot.data.melt$plot == 'log2(Relative abundances)'])-20
ab.plot.data.melt$value[ab.plot.data.melt$plot == 'log(Bias-corrected abundances)'] <-
      ab.plot.data.melt$value[ab.plot.data.melt$plot == 'log(Bias-corrected abundances)']+20

#### FULL PLOT ####

# merge data
plot.data <- merge(ab.plot.data.melt, fc.plot.data, all=TRUE, sort=FALSE)
plot.data$plot <- factor(plot.data$plot, levels=unique(plot.data$plot))
plot.data$variable <- factor(gsub('Candidatus Methanomassiliicoccus intestinalis',
                                  'Candidatus Methanomassiliicoccus\nintestinalis',
                                  plot.data$variable),
                                  levels=gsub('Candidatus Methanomassiliicoccus intestinalis',
                                              'Candidatus Methanomassiliicoccus\nintestinalis',
                                              unique(plot.data$variable)))

```

```
# create breaks and break labels for plot
breaks <- c(-40,-35,-30,-25,-20,-15,-10,-5,-2,0,2,5,10,20,25,30,35)
break_labels <- c(paste(breaks[1:5]+20,'\n(',gsub('e\\+00','',gsub('e-0','e-',
                        formatC(2^(breaks[1:5]+20),format='e',digits=0))),')',sep=''),
                  gsub('1x','0x', paste(abs(breaks[6:13])+1, 'x',sep='')),
                  paste(breaks[14:17]-20,'\n(',round(exp(breaks[14:17]-20),1),')',sep=''))

# create plot
g1 <- ggplot(data=plot.data[grep('log', plot.data$plot),],
             aes(x=variable, y=value, fill=as.character(Case_status))) +
        geom_boxplot(notch=FALSE, outlier.size=0.5) +
        geom_errorbar(inherit.aes=FALSE,
                      data=plot.data[plot.data$plot=='Absolute fold change with 95%CI',],
                      aes(x=variable, ymin=FC_lower_mod, ymax=FC_upper_mod,
                          color=color, linetype=line),
                      width=0, position=position_dodge(0.75), size=0.75) +
        geom_point(inherit.aes=FALSE,
                   data=plot.data[plot.data$plot=='Absolute fold change with 95%CI',],
                   aes(x=variable, y=FC_mod, color=color, pch=line),
                   position=position_dodge(0.75), size=1.75) +
        geom_hline(data=plot.data[plot.data$plot=='Absolute fold change with 95%CI',],
                   aes(yintercept=0),
                   size=0.5, linetype='dashed', alpha=0.5) +
        facet_nested(. ~ plot, scales='free', space='free_y', switch='y',
                     strip=strip_nested(text_y=list(element_text(angle=0))),
                     labeller=labeller(group=label_wrap_gen(width=10),
                                       sub_group=label_wrap_gen(width=10))) +
        scale_x_discrete(position='bottom') +
        scale_y_continuous(position='right', breaks=breaks, labels=break_labels) +
        coord_flip() +
        scale_fill_manual(values=c("#E69F00", "#00BFC4")) +
        scale_color_manual(values=c("blue", "red"), labels=c("elevated","depleted")) +
        scale_linetype_manual(values=c("11", "solid")) +
        scale_shape_manual(values=c(16, 15)) +
        guides(fill=guide_legend(order=1, title="Subject group", title.position="top"),
               color=guide_legend(order=2, title="Fold change direction", title.position="top"),
               linetype=guide_legend(title="Fold change source", title.position="top", reverse=TRUE),
               pch=guide_legend(title="Fold change source", title.position="top", reverse=TRUE)) +
        theme(legend.position="top", legend.key=element_blank(),
              legend.title=element_text(size=12), legend.text=element_text(size=12),
              axis.title.x=element_blank(), axis.text.x=element_text(size=10),
              axis.title.y=element_blank(), axis.text.y=element_text(size=10),
              strip.text=element_text(size=12),
              strip.background=element_rect(fill='gray90', color='gray'),
              strip.placement="outside", panel.spacing.y=unit(0.5, "lines"))
ggsave(
'PDShotgunAnalysis_out/3.Taxonomic_associations/PD_associated_species_distributions_foldchanges_1.pdf',
g1, device='pdf', width=12, height=30)

#### REDUCED PLOT ####

# merge data
targets <- fc.plot.data$variable[fc.plot.data$line == 'MaAsLin2' &
                                 (round(fc.plot.data$FC,2) >= 1.75 |
```

```
                                  round(fc.plot.data$FC,2) <= 0.57)]
plot.data <- merge(ab.plot.data.melt[ab.plot.data.melt$variable %in% targets,],
                   fc.plot.data[fc.plot.data$variable %in% targets,],
                   all=TRUE, sort=FALSE)
plot.data$plot <- factor(plot.data$plot, levels=unique(plot.data$plot))

# truncate upper limits of fold changes to < 10x
plot.data$FC_lower_mod[plot.data$plot == 'Absolute fold change with 95%CI' &
                       plot.data$FC_lower < 0.1] <- -9
plot.data$FC_upper_mod[plot.data$plot == 'Absolute fold change with 95%CI' &
                       plot.data$FC_upper > 10] <- 9

# create breaks and break labels for plot
breaks <- c(-40,-35,-30,-25,-20,-9,-5,-2,0,2,5,9,20,25,30,35)
break_labels <- c(paste(breaks[1:5]+20,'\n(',gsub('e\\+00','',gsub('e-0','e-',
                        formatC(2^(breaks[1:5]+20),format='e',digits=0))),')',sep=''),
                  gsub('1x','0x', paste(abs(breaks[6:12])+1, 'x',sep='')),
                  paste(breaks[13:16]-20,'\n(',round(exp(breaks[13:16]-20),1),')',sep=''))

# create plot
g2 <- ggplot(data=plot.data[grep('log', plot.data$plot),],
             aes(x=variable, y=value, fill=as.character(Case_status))) +
        geom_boxplot(notch=FALSE, outlier.size=0.5) +
        geom_errorbar(inherit.aes=FALSE,
                      data=plot.data[plot.data$plot=='Absolute fold change with 95%CI',],
                      aes(x=variable, ymin=FC_lower_mod, ymax=FC_upper_mod,
                          color=color, linetype=line),
                      width=0, position=position_dodge(0.75), size=0.75) +
        geom_point(inherit.aes=FALSE,
                   data=plot.data[plot.data$plot=='Absolute fold change with 95%CI',],
                   aes(x=variable, y=FC_mod, color=color, pch=line),
                   position=position_dodge(0.75), size=1.75) +
        geom_hline(data=plot.data[plot.data$plot=='Absolute fold change with 95%CI',],
                   aes(yintercept=0),
                   size=0.5, linetype='dashed', alpha=0.5) +
        facet_nested(. ~ plot, scales='free', space='free_y', switch='y',
                     strip=strip_nested(text_y=list(element_text(angle=0))),
                     labeller=labeller(group=label_wrap_gen(width=10),
                                       sub_group=label_wrap_gen(width=10))) +
        scale_x_discrete(position='bottom') +
        scale_y_continuous(position='right', breaks=breaks, labels=break_labels) +
        coord_flip() +
        scale_fill_manual(values=c("#E69F00", "#00BFC4")) +
        scale_color_manual(values=c("blue", "red"), labels=c("elevated","depleted")) +
        scale_linetype_manual(values=c("11", "solid")) +
        scale_shape_manual(values=c(16, 15)) +
        guides(fill=guide_legend(order=1, title="Subject group", title.position="top"),
               color=guide_legend(order=2, title="Fold change direction", title.position="top"),
               linetype=guide_legend(title="Fold change source", title.position="top", reverse=TRUE),
               pch=guide_legend(title="Fold change source", title.position="top", reverse=TRUE)) +
        theme(legend.position="top", legend.key=element_blank(),
              legend.title=element_text(size=12), legend.text=element_text(size=12),
              axis.title.x=element_blank(), axis.text.x=element_text(size=10),
              axis.title.y=element_blank(), axis.text.y=element_text(size=10),
```

```
                 strip.text=element_text(size=12),
                 strip.background=element_rect(fill='gray90', color='gray'),
                 strip.placement="outside", panel.spacing.y=unit(0.5, "lines"))
ggsave(
'PDShotgunAnalysis_out/3.Taxonomic_associations/PD_associated_species_distributions_foldchanges_2.pdf',
g2, device='pdf', width=12, height=18)
```

Sex, age, and confounder analysis

To see how PD-species associations are affected when adusting for age and sex and extrinsic PD-associated subject data (variables associated with PD from earlier subject metadata analysis that are exposure variables not intrinsically or biologically related to the disease), re-ran MaAsLin2 for PD-associated species adjusting for these variables.

- Note: data for pain meds and sleep aid were missing for 5 subjects who had their stool samples collected with sterile swabs, therefore, these subjects were excluded from these analyses to control for collection method instead of adjusting for it in the model.

MaAsLin2 was ran once adjusting for age and sex, and then again adjusting for the 7 potential confounding variables. In both analyses total sequence count per sample (standardized) was also adjusted for. After running the confounding analysis, a table was created tagging which model variables associated with each taxon.

```
#### SEX, AGE, & CONFOUNDER ANALYSES ####

# recode categorical variable to get correct effect direction and scale numeric variables
sample_data(ra.ps.s)$Sex <- dplyr::recode(sample_data(ra.ps.s)$Sex, M=1, F=0)
sample_data(ra.ps.s)$Do_you_drink_alcohol <-
                 dplyr::recode(sample_data(ra.ps.s)$Do_you_drink_alcohol, Y=1, N=0)
sample_data(ra.ps.s)$Laxatives <- dplyr::recode(sample_data(ra.ps.s)$Laxatives, Y=1, N=0)
sample_data(ra.ps.s)$Probiotic <- dplyr::recode(sample_data(ra.ps.s)$Probiotic, Y=1, N=0)
sample_data(ra.ps.s)$Pain_med <- dplyr::recode(sample_data(ra.ps.s)$Pain_med, Y=1, N=0)
sample_data(ra.ps.s)$Depression_anxiety_mood_med <-
                 dplyr::recode(sample_data(ra.ps.s)$Depression_anxiety_mood_med, Y=1, N=0)
sample_data(ra.ps.s)$Antihistamines <- dplyr::recode(sample_data(ra.ps.s)$Antihistamines, Y=1, N=0)
sample_data(ra.ps.s)$Sleep_aid <- dplyr::recode(sample_data(ra.ps.s)$Sleep_aid, Y=1, N=0)
sample_data(ra.ps.s)$age_scaled <- scale(sample_data(ra.ps.s)$Age_at_collection)

#### SEX AND AGE ####

# prep temporary directory for MaAsLin2 output
system('
if [ ! -d "temp_directory" ]
then
  mkdir temp_directory
fi
')

# perform differential abundance analysis for PD-associated species using
# linear regression with log2 transformed relative abundances
variables <- c('Case_status', 'seqs_scaled', 'collection_method', 'Sex', 'age_scaled')

ps <- phyloseq(otu_table(prune_taxa(sig.species, ra.ps.s))/100, sample_data(ra.ps.s))
suppress(
lm.s.adj <- MaAsLin2.plus(ps=ps,
```

```r
                                metadata=variables,
                                output='temp_directory',
                                min_prevalence=0.05,
                                normalization='NONE',
                                max_significance=0.05,
                                standardize=FALSE,
                                plot_heatmap=FALSE,
                                plot_scatter=FALSE)
)

# coalesce results for sex and age analysis
res.summ <- data.frame(Variable=lm.s.adj$result.summary$Variable,
                            Species=gsub('_', ' ',
                                         gsub('s__', '',
                                              sapply(strsplit(lm.s.adj$result.summary$Feature, "\\|"),
                                                     function(x){x[7]}))),
                            lm.s.adj$result.summary[,c('Beta','SE','P','FDR','FC')],
                            `FC lower`=lm.s.adj$result.summary$FC_lower,
                            `FC upper`=lm.s.adj$result.summary$FC_upper,
                            check.names=FALSE)
res.summ <- res.summ[order(res.summ$Species),]
res.summ$Variable <- gsub('Case_status', 'Case status',
                                res.summ$Variable)
res.summ$Variable <- gsub('seqs_scaled', 'Total sequence count (standardized)',
                                res.summ$Variable)
res.summ$Variable <- gsub('collection_method', 'Stool collection method',
                                res.summ$Variable)
res.summ$Variable <- gsub('age_scaled', 'Age (standardized)',
                                res.summ$Variable)

# initialize worbook
wb <- createWorkbook()

# add results for sex and age analysis and format
addWorksheet(wb, 'Sex age results')
writeData(wb, 'Sex age results', res.summ, keepNA=TRUE, colNames=TRUE)
setColWidths(wb, 'Sex age results', cols=seq_len(ncol(res.summ)),
             widths=c(22, 34, rep(10,7))) ### format cells
addStyle(wb, 'Sex age results', cols=seq_len(ncol(res.summ)),
         rows=1, style=bold, stack=TRUE) ### font
addStyle(wb, 'Sex age results', cols=seq_len(ncol(res.summ)),
         rows=c(1,2,(nrow(res.summ)+2)), ### borders
         gridExpand=TRUE, style=horizontal_border_med, stack=TRUE)

#### PD EXTRINSIC CONFOUNDERS ####

# perform differential abundance analysis for PD-associated species using
# linear regression with log2 transformed relative abundances
# (Note: subjects who had sample collected with sterile swab are removed prior to testing)
variables <- c('Case_status', 'seqs_scaled', 'Do_you_drink_alcohol',
               'Laxatives', 'Probiotic', 'Pain_med',
               'Depression_anxiety_mood_med', 'Antihistamines', 'Sleep_aid')

ps <- phyloseq(otu_table(prune_taxa(sig.species,
```

```
                                        subset_samples(ra.ps.s,
                                                         collection_method==0)))/100,
                 sample_data(ra.ps.s))
suppress(
lm.s.adj <- MaAsLin2.plus(ps=ps,
                          metadata=variables,
                          output='temp_directory',
                          min_prevalence=0.05,
                          normalization='NONE',
                          max_significance=0.05,
                          standardize=FALSE,
                          plot_heatmap=FALSE,
                          plot_scatter=FALSE)
)

# remove temporary output directory
system('rm -r temp_directory')

# coalesce results for confounder analysis
res.summ <- data.frame(Variable=lm.s.adj$result.summary$Variable,
                       Species=gsub('_', ' ',
                                    gsub('s__', '',
                                         sapply(strsplit(lm.s.adj$result.summary$Feature, "\\|"),
                                                function(x){x[7]}))),
                       lm.s.adj$result.summary[,c('Beta','SE','P','FDR','FC')],
                       `FC lower`=lm.s.adj$result.summary$FC_lower,
                       `FC upper`=lm.s.adj$result.summary$FC_upper,
                       check.names=FALSE)
res.summ <- res.summ[order(res.summ$Species),]
res.summ$Variable <- gsub('Case_status', 'Case status',
                          res.summ$Variable)
res.summ$Variable <- gsub('seqs_scaled', 'Total sequence count (standardized)',
                          res.summ$Variable)
res.summ$Variable <- gsub('Do_you_drink_alcohol', 'Alcohol',
                          res.summ$Variable)
res.summ$Variable <- gsub('Pain_med', 'Pain medication',
                          res.summ$Variable)
res.summ$Variable <- gsub('Depression_anxiety_mood_med',
                          'Depression, anxiety, mood medication',
                          res.summ$Variable)
res.summ$Variable <- gsub('Sleep_aid', 'Sleep aid',
                          res.summ$Variable)

# add results for confounder analysis and format
addWorksheet(wb, 'Confounder var results')
writeData(wb, 'Confounder var results', res.summ, keepNA=TRUE, colNames=TRUE)
setColWidths(wb, 'Confounder var results', cols=seq_len(ncol(res.summ)),
             widths=c(22, 34, rep(10,7))) ### format cells
addStyle(wb, 'Confounder var results', cols=seq_len(ncol(res.summ)),
         rows=1, style=bold, stack=TRUE) ### font
addStyle(wb, 'Confounder var results', cols=seq_len(ncol(res.summ)),
         rows=c(1,2,(nrow(res.summ)+2)), ### borders
         gridExpand=TRUE, style=horizontal_border_med, stack=TRUE)
```

```
# make table of what species associated with what variable
var.breakdown <- data.frame(Feature=unique(lm.s.adj$result.summary$Feature),
                            `Associated variable`=NA, check.names=FALSE)
for (taxa in seq_len(nrow(var.breakdown))){
  taxa.name <- var.breakdown$Feature[taxa]
  sig.var <- lm.s.adj$result.summary[lm.s.adj$result.summary$Feature == taxa.name &
                                     round(lm.s.adj$result.summary$FDR,1) <= 0.1,]
  if (nrow(sig.var) > 0){
    sig.var.lab <- c()
    for (var in seq_len(nrow(sig.var))){
      var.res <- sig.var[var,]
      if (!(is.na(var.res$FC))){
        if (var.res$FC > 1 && var.res$FDR < 0.05){sig.var.lab <-
                    c(sig.var.lab, paste(var.res$Variable,'++',sep=''))}
        if (var.res$FC > 1 && var.res$FDR >= 0.05){sig.var.lab <-
                    c(sig.var.lab, paste(var.res$Variable,'+',sep=''))}
        if (var.res$FC < 1 && var.res$FDR < 0.05){sig.var.lab <-
                    c(sig.var.lab, paste(var.res$Variable,'--',sep=''))}
        if (var.res$FC < 1 && var.res$FDR >= 0.05){sig.var.lab <-
                    c(sig.var.lab, paste(var.res$Variable,'-',sep=''))}
      }
    }
    var.breakdown$`Associated variable`[taxa] <- paste(sig.var.lab, collapse=',')
  }
}
var.breakdown$`Associated variable`[is.na(var.breakdown$`Associated variable`)] <- ''

# replace variable names with smaller names
var.breakdown$`Associated variable` <- gsub('Case_status', 'PD',
                                            var.breakdown$`Associated variable`)
var.breakdown$`Associated variable` <- gsub('Depression_anxiety_mood_med', 'Mood med',
                                            var.breakdown$`Associated variable`)
var.breakdown$`Associated variable` <- gsub('Do_you_drink_alcohol', 'Alcohol',
                                            var.breakdown$`Associated variable`)
var.breakdown$`Associated variable` <- gsub('_', ' ',
                                            var.breakdown$`Associated variable`)

# coalesce results for asscociating variables
res.summ <- data.frame(Species=gsub('_', ' ',
                                    gsub('s__', '',
                                         sapply(strsplit(var.breakdown$Feature, "\\|"),
                                                function(x){x[7]}))),
                       `Associated variable`=var.breakdown$`Associated variable`,
                       check.names=FALSE)
res.summ <- res.summ[order(res.summ$Species),]

# add results for associating variables and format
addWorksheet(wb, 'Assoc confound var')
writeData(wb, 'Assoc confound var', res.summ, keepNA=TRUE, colNames=TRUE)
setColWidths(wb, 'Assoc confound var', cols=seq_len(ncol(res.summ)),
             widths=c(22, 30)) ### format cells
addStyle(wb, 'Assoc confound var', cols=seq_len(ncol(res.summ)),
         rows=1, style=bold, stack=TRUE) ### font
addStyle(wb, 'Assoc confound var', cols=seq_len(ncol(res.summ)),
```

```
        rows=c(1,2,(nrow(res.summ)+2)), ### borders
        gridExpand=TRUE, style=horizontal_border_med, stack=TRUE)

# save workbook
saveWorkbook(wb,
    'PDShotgunAnalysis_out/3.Taxonomic_associations/PD_associated_species_adj_covariates.xlsx',
    overwrite=TRUE)
```

Correlation networks

In order to get an inferred ecological picture of the PD and NHC gut microbiome, co-occurence networks were constructed using SparCC. To construct the correlation networks, pairwise correlations and corresponding P-values were calculated using SparCC (FastSpar C++ implementation) on species count data. These correlations were visulized as a network using the GUI program `Gephi`.

SparCC

SparCC was used to calculate pairwise correlations between species in PD and NHC samples separately.

- SparCC (`FastSpar`) was performed using 100 iterations (double the default) to get inter-random seed stable correlation calculations with the default `--threshold` parameter of 0.1.
- To calculate p-values for SparCC correlations, the input PD and NHC data were randomly permuted 1,000 times to make 1,000 random datasets. SparCC correlations were then calculated on these random datasets. P-values were calculated by comparing SparCC correlations computed on random datasets to those computed on the real data to see how many instances the real data correlations were better than those derived from random.

```
#### SPARCC CORRELATIONS ####

# separate to PD and healthy NHC data making sure to remove
# UNKNOWN group and any taxa with all 0 after subsetting subjects
ps.pd.s <- filter_taxa(prune_taxa(taxa_names(abun.ps.s)[grep('UNKNOWN',
                                                             taxa_names(abun.ps.s),
                                                             invert=TRUE)],
                                  subset_samples(abun.ps.s, Case_status == 1)),
                       function(x){sum(x > 0) > 0}, TRUE)
ps.hc.s <- filter_taxa(prune_taxa(taxa_names(abun.ps.s)[grep('UNKNOWN',
                                                             taxa_names(abun.ps.s),
                                                             invert=TRUE)],
                                  subset_samples(abun.ps.s, Case_status == 0)),
                       function(x){sum(x > 0) > 0}, TRUE)

# format for input to SparCC
pd.s <- data.frame(OTU_id=sapply(strsplit(as.character(taxa_names(ps.pd.s)), "s__"),
                                 function(x){x[2]}),
                   t(otu_table(ps.pd.s)), check.names=FALSE)

hc.s <- data.frame(OTU_id=sapply(strsplit(as.character(taxa_names(ps.hc.s)), "s__"),
                                 function(x){x[2]}),
                   t(otu_table(ps.hc.s)), check.names=FALSE)

write.table(pd.s, 'PDShotgunAnalysis_out/4.a.Network_analysis/Species_SparCC_PD_table.txt',
            row.names=FALSE, quote=FALSE, sep='\t')
```

```
write.table(hc.s, 'PDShotgunAnalysis_out/4.a.Network_analysis/Species_SparCC_HC_table.txt',
            row.names=FALSE, quote=FALSE, sep='\t')

# calculate SparCC correlations in HPC environment
system('
fastspar --iterations 100 \\
--threads 10 \\
--threshold 0.1 \\
--seed 1234 \\
--otu_table PDShotgunAnalysis_out/4.a.Network_analysis/Species_SparCC_PD_table.txt \\
--correlation PDShotgunAnalysis_out/4.a.Network_analysis/Species_SparCC_PD_Cor_Matrix.txt \\
--covariance PDShotgunAnalysis_out/4.a.Network_analysis/Species_SparCC_PD_Cov_Matrix.txt
')

system('
fastspar --iterations 100 \\
--threads 10 \\
--threshold 0.1 \\
--seed 1234 \\
--otu_table PDShotgunAnalysis_out/4.a.Network_analysis/Species_SparCC_HC_table.txt \\
--correlation PDShotgunAnalysis_out/4.a.Network_analysis/Species_SparCC_HC_Cor_Matrix.txt \\
--covariance PDShotgunAnalysis_out/4.a.Network_analysis/Species_SparCC_HC_Cov_Matrix.txt
')

# create directory for temporary permuted data
system('
if [ ! -d "temp_data" ]
then
  mkdir temp_data
  mkdir temp_data/ErrorOut
  mkdir temp_data/Output
fi
')

# create randomly permuted datasets
system('
fastspar_bootstrap \\
--otu_table PDShotgunAnalysis_out/4.a.Network_analysis/Species_SparCC_PD_table.txt \\
--threads 20 \\
--number 1000 \\
--seed 1234 \\
--prefix temp_data/PD_temp_data
')

system('
fastspar_bootstrap \\
--otu_table PDShotgunAnalysis_out/4.a.Network_analysis/Species_SparCC_HC_table.txt \\
--threads 20 \\
--number 1000 \\
--seed 1234 \\
--prefix temp_data/HC_temp_data
')
```

```
# calculated correlations for each permuted dataset
# (Note: this section is set up to be run on a HPC with a SLURM scheduler)
system('
echo "#!/bin/bash" > bash_script.sh
echo "#SBATCH --partition=amd-hdr100" >> bash_script.sh
echo "#SBATCH --job-name=SparCC" >> bash_script.sh
echo "#SBATCH --error=temp_data/ErrorOut/SparCC_%A_%a.err" >> bash_script.sh
echo "#SBATCH --output=temp_data/Output/SparCC_%A_%a.out" >> bash_script.sh
echo "#SBATCH --time=2:00:00" >> bash_script.sh
echo "#SBATCH --ntasks=1" >> bash_script.sh
echo "#SBATCH --cpus-per-task=1" >> bash_script.sh
echo "#SBATCH --mem-per-cpu=4000" >> bash_script.sh
echo "#SBATCH --mail-type=FAIL" >> bash_script.sh
echo "#SBATCH --mail-user=wallenz@uab.edu" >> bash_script.sh
echo "#SBATCH --array=0-999" >> bash_script.sh
echo "#SBATCH --wait" >> bash_script.sh
echo " " >> bash_script.sh
echo "source ~/miniconda3/etc/profile.d/conda.sh" >> bash_script.sh
echo "conda activate fastspar" >> bash_script.sh
echo " " >> bash_script.sh
echo "fastspar --iterations 100 \\\\" >> bash_script.sh
echo "--threads 10 \\\\" >> bash_script.sh
echo "--threshold 0.1 \\\\" >> bash_script.sh
echo "--seed 1234 \\\\" >> bash_script.sh
echo "--otu_table temp_data/PD_temp_data_\\${SLURM_ARRAY_TASK_ID}.tsv \\\\" >> bash_script.sh
echo "--correlation temp_data/PD_Cor_Mat_\\${SLURM_ARRAY_TASK_ID}.tsv \\\\" >> bash_script.sh
echo "--covariance temp_data/PD_Cov_Mat_\\${SLURM_ARRAY_TASK_ID}.tsv" >> bash_script.sh
echo " " >> bash_script.sh
echo "fastspar --iterations 100 \\\\" >> bash_script.sh
echo "--threads 10 \\\\" >> bash_script.sh
echo "--threshold 0.1 \\\\" >> bash_script.sh
echo "--seed 1234 \\\\" >> bash_script.sh
echo "--otu_table temp_data/HC_temp_data_\\${SLURM_ARRAY_TASK_ID}.tsv \\\\" >> bash_script.sh
echo "--correlation temp_data/HC_Cor_Mat_\\${SLURM_ARRAY_TASK_ID}.tsv \\\\" >> bash_script.sh
echo "--covariance temp_data/HC_Cov_Mat_\\${SLURM_ARRAY_TASK_ID}.tsv" >> bash_script.sh
')

system('sbatch bash_script.sh')

# calculate permuted p-values for each correlation
system('
fastspar_pvalues --threads 20 \\
--otu_table PDShotgunAnalysis_out/4.a.Network_analysis/Species_SparCC_PD_table.txt \\
--correlation PDShotgunAnalysis_out/4.a.Network_analysis/Species_SparCC_PD_Cor_Matrix.txt \\
--prefix temp_data/PD_Cor_Mat_ \\
--permutations 1000 \\
--outfile PDShotgunAnalysis_out/4.a.Network_analysis/Species_SparCC_PD_Pval_Matrix.txt
')

system('
fastspar_pvalues --threads 20 \\
--otu_table PDShotgunAnalysis_out/4.a.Network_analysis/Species_SparCC_HC_table.txt \\
--correlation PDShotgunAnalysis_out/4.a.Network_analysis/Species_SparCC_HC_Cor_Matrix.txt \\
--prefix temp_data/HC_Cor_Mat_ \\
```

```
--permutations 1000 \\
--outfile PDShotgunAnalysis_out/4.a.Network_analysis/Species_SparCC_HC_Pval_Matrix.txt
')

# clean up
system('
rm -r temp_data
rm bash_script.sh
')
```

Community detection and creating node and edge files for network visualization

Once SparCC correlations and p-values were computed, they were formatted into node and edge data.frames and files that could be imported into `igraph` and the visualization program `Gephi`.

- Before importing into `igraph`, SparCC correlations were filtered for species correlations that resulted in a permuted p-value of < 0.05.
- Once imported into `igraph`, edges were further filtered for those with $r > 0.2$, and nodes were then filtered for those who had edges remaining.
- Communities of nodes (species) were then detected using the Louvain algorithm via `cluster_louvain` function in `igraph`.
- Degree for each species was calculated using the `degree` function in `igraph`.
- Node and edge CSV files were outputted from R, and PD and NHC networks were plotted in `Gephi` using the force directed `Force Atlas 2` algorithm to position nodes, then coloring first by species cluster memberships (detected with Louvain algorithm) then by PD-associated species differentiating between elevated (blue) vs reduced (red) species.

```
#### COMMUNITY DETECTION & PREPARING NODE/EDGE FILES ####

# read in SparCC correlations and pvalues
pd.cor.s <- read.table('PDShotgunAnalysis_out/4.a.Network_analysis/Species_SparCC_PD_Cor_Matrix.txt',
                       row.names="#OTU ID", header=TRUE, stringsAsFactors=FALSE,
                       check.names=FALSE, comment.char='', sep='\t')
pd.pval.s <- read.table('PDShotgunAnalysis_out/4.a.Network_analysis/Species_SparCC_PD_Pval_Matrix.txt',
                        row.names="#OTU ID", header=TRUE, stringsAsFactors=FALSE,
                        check.names=FALSE, comment.char='', sep='\t')

hc.cor.s <- read.table('PDShotgunAnalysis_out/4.a.Network_analysis/Species_SparCC_HC_Cor_Matrix.txt',
                       row.names="#OTU ID", header=TRUE, stringsAsFactors=FALSE,
                       check.names=FALSE, comment.char='', sep='\t')
hc.pval.s <- read.table('PDShotgunAnalysis_out/4.a.Network_analysis/Species_SparCC_HC_Pval_Matrix.txt',
                        row.names="#OTU ID", header=TRUE, stringsAsFactors=FALSE,
                        check.names=FALSE, comment.char='', sep='\t')

# subset and merge result data for case status results
res.s <- merge(ancom.s$result.summary[ancom.s$result.summary$Variable == 'Case_status' &
                                      ancom.s$result.summary$Feature != 'UNKNOWN',],
               lm.s$result.summary[lm.s$result.summary$Variable == 'Case_status',],
               by=c('Variable','Feature','N1','N2'), suffix=c('_ancombc','_maaslin'))
res.s <- res.s[order(res.s$P_ancom, decreasing=TRUE),]

# create node file
nodes.s <- data.frame(Id=sapply(strsplit(as.character(res.s$Feature), "s__"),
                                function(x){x[2]}),
```

```
                         Label=sapply(strsplit(as.character(res.s$Feature), "s__"),
                                        function(x){x[2]}),
                         ANCOMBC_Beta=res.s$Beta_ancombc,
                         ANCOMBC_FDR=res.s$FDR_ancombc,
                         MaAsLin2_Beta=res.s$Beta_maaslin,
                         MaAsLin2_FDR=res.s$FDR_maaslin,
                         row.names=NULL)
nodes.s$`PD-associated` <- 'No'
nodes.s$`PD-associated`[((nodes.s$ANCOMBC_FDR < 0.05 & round(nodes.s$MaAsLin2_FDR,1) <= 0.1) |
                      (round(nodes.s$ANCOMBC_FDR,1) <= 0.1 & nodes.s$MaAsLin2_FDR < 0.05)) &
                      (nodes.s$ANCOMBC_Beta > 0 & nodes.s$MaAsLin2_Beta > 0)] <- 'Yes_Increased'
nodes.s$`PD-associated`[((nodes.s$ANCOMBC_FDR < 0.05 & round(nodes.s$MaAsLin2_FDR,1) <= 0.1) |
                      (round(nodes.s$ANCOMBC_FDR,1) <= 0.1 & nodes.s$MaAsLin2_FDR < 0.05)) &
                      (nodes.s$ANCOMBC_Beta < 0 & nodes.s$MaAsLin2_Beta < 0)] <- 'Yes_Decreased'
nodes.s$`PD-associated`[is.na(nodes.s$ANCOMBC_FDR) & is.na(nodes.s$MaAsLin2_FDR)] <- 'Not_tested'
nodes.s <- nodes.s[,grep('Beta|FDR', colnames(nodes.s), invert=TRUE)]

# create edge files for PD and NHC
corr.direction <- c()
corr.direction[pd.cor.s[lower.tri(pd.cor.s)] > 0] <- "+"
corr.direction[pd.cor.s[lower.tri(pd.cor.s)] < 0] <- "-"
pd.edges.s <- data.frame(Source=t(combn(rownames(pd.cor.s), 2))[,1],
                         Target=t(combn(rownames(pd.cor.s), 2))[,2],
                         Weight=abs(pd.cor.s[lower.tri(pd.cor.s)]),
                               `Direction of correlation`=corr.direction,
                         `Correlation P-value`=pd.pval.s[lower.tri(pd.pval.s)],
                               check.names=FALSE)

corr.direction <- c()
corr.direction[hc.cor.s[lower.tri(hc.cor.s)] > 0] <- "+"
corr.direction[hc.cor.s[lower.tri(hc.cor.s)] < 0] <- "-"
hc.edges.s <- data.frame(Source=t(combn(rownames(hc.cor.s), 2))[,1],
                         Target=t(combn(rownames(hc.cor.s), 2))[,2],
                         Weight=abs(hc.cor.s[lower.tri(hc.cor.s)]),
                               `Direction of correlation`=corr.direction,
                         `Correlation P-value`=hc.pval.s[lower.tri(hc.pval.s)],
                               check.names=FALSE)

# tag edges that contains a significant taxon
pd.edges.s$`PD-associated`[pd.edges.s$Source %in%
                           nodes.s$Id[grep('Yes', nodes.s$`PD-associated`)] |
                           pd.edges.s$Target %in%
                           nodes.s$Id[grep('Yes', nodes.s$`PD-associated`)]] <- "Yes"
pd.edges.s$`PD-associated`[is.na(pd.edges.s$`PD-associated`)] <- "No"

hc.edges.s$`PD-associated`[hc.edges.s$Source %in%
                           nodes.s$Id[grep('Yes', nodes.s$`PD-associated`)] |
                           hc.edges.s$Target %in%
                           nodes.s$Id[grep('Yes', nodes.s$`PD-associated`)]] <- "Yes"
hc.edges.s$`PD-associated`[is.na(hc.edges.s$`PD-associated`)] <- "No"

# filter edges for significant correlations (permuted pvalue < 0.05)
pd.edges.s <- pd.edges.s[pd.edges.s$`Correlation P-value` < 0.05,]

```

```r
hc.edges.s <- hc.edges.s[hc.edges.s$`Correlation P-value` < 0.05,]

# import data into igraph
pd.igraph.s <- graph_from_data_frame(pd.edges.s, directed=FALSE, vertices=nodes.s)
E(pd.igraph.s)$weight <- E(pd.igraph.s)$Weight

hc.igraph.s <- graph_from_data_frame(hc.edges.s, directed=FALSE, vertices=nodes.s)
E(hc.igraph.s)$weight <- E(hc.igraph.s)$Weight

# remove edges with correlations < 0.2
pd.igraph.s <- delete_edges(pd.igraph.s, which(E(pd.igraph.s)$weight < 0.2))

hc.igraph.s <- delete_edges(hc.igraph.s, which(E(hc.igraph.s)$weight < 0.2))

# remove nodes with degree of 0
V(pd.igraph.s)$Degree <- degree(pd.igraph.s, normalized=FALSE)
pd.igraph.s <- delete_vertices(pd.igraph.s, V(pd.igraph.s)$Degree == 0)

V(hc.igraph.s)$Degree <- degree(hc.igraph.s, normalized=FALSE)
hc.igraph.s <- delete_vertices(hc.igraph.s, V(hc.igraph.s)$Degree == 0)

# calculate community membership and modularity of networks
pd.clusters <- cluster_louvain(pd.igraph.s)
V(pd.igraph.s)$Cluster <- pd.clusters$membership

hc.clusters <- cluster_louvain(hc.igraph.s)
V(hc.igraph.s)$Cluster <- hc.clusters$membership

# add degree and community memberships to node files
nodes.s <- merge(nodes.s,
                 data.frame(Id=V(pd.igraph.s)$name,
                            `Degree in PD`=V(pd.igraph.s)$Degree,
                            `Cluster in PD`=V(pd.igraph.s)$Cluster,
                            check.names=FALSE),
                 by='Id', all=TRUE)
nodes.s <- merge(nodes.s,
                 data.frame(Id=V(hc.igraph.s)$name,
                            `Degree in NHC`=V(hc.igraph.s)$Degree,
                            `Cluster in NHC`=V(hc.igraph.s)$Cluster,
                            check.names=FALSE),
                 by='Id', all=TRUE)
nodes.s$`Degree in PD`[is.na(nodes.s$`Degree in PD`)] <- 0
nodes.s$`Degree in NHC`[is.na(nodes.s$`Degree in NHC`)] <- 0
nodes.s$`Cluster in PD`[is.na(nodes.s$`Cluster in PD`)] <- "none"
nodes.s$`Cluster in NHC`[is.na(nodes.s$`Cluster in NHC`)] <- "none"

# output node and edge files
write.csv(nodes.s,
          "PDShotgunAnalysis_out/4.a.Network_analysis/Species_SparCC_node_file.csv",
          quote=FALSE, row.names=FALSE)
write.csv(pd.edges.s,
          "PDShotgunAnalysis_out/4.a.Network_analysis/Species_SparCC_PD_edge_file.csv",
          quote=FALSE, row.names=FALSE)
write.csv(hc.edges.s,
```

```
            "PDShotgunAnalysis_out/4.a.Network_analysis/Species_SparCC_HC_edge_file.csv",
            quote=FALSE, row.names=FALSE)
```

Analyses of gene families and pathways

Preparing count data for downstream analyses

```
#### PREPARE RELATIVE ABUNDANCE AND COUNT DATA ####

# read in metadata
metadata <- data.frame(read_xlsx('Source_Data.xlsx', sheet='subject_metadata'))
rownames(metadata) <- metadata$sample_name

# read in tables that were previously generated by functional profiling
gene <- data.frame(read_xlsx('Source_Data.xlsx', sheet='humann_KO_group_counts'))

path <- data.frame(read_xlsx('Source_Data.xlsx', sheet='humann_pathway_counts'))

# order same as metadata
gene <- gene[,c('Gene.Family',metadata$sample_name)]

path <- path[,c('Pathway',metadata$sample_name)]

# make table sample x feature
rownames(gene) <- gene$Gene.Family
gene <- data.frame(t(gene[,-1]), check.names=FALSE)

rownames(path) <- path$Pathway
path <- data.frame(t(path[,-1]), check.names=FALSE)

# create phyloseq objects for gene families and pathways
gene.ps <- phyloseq(otu_table(as.matrix(gene), taxa_are_rows=FALSE),
                    sample_data(metadata))
path.ps <- phyloseq(otu_table(as.matrix(path), taxa_are_rows=FALSE),
                    sample_data(metadata))
```

Differential abundance of gene families and pathways

MWAS

To peform the differential abundance analyses of KO groups and pathways, abundances of detected pathways and KO groups were provided to ANCOM-BC and MaAsLin2. Parameters and model formula used for ANCOM-BC and MaAsLin2 were kept the same as what was used in taxonomic-based analyses, except KO group and pathway abundances were total sum scaled prior to analyzing with MaAsLin2 since abundances are being used as input this time instead of relative abundances.

```
#### KO GROUP AND PATHWAY MWAS ####

# recode categorical variable to get correct effect direction and scale total sequence count
sample_data(gene.ps)$Case_status <-
               dplyr::recode(sample_data(gene.ps)$Case_status, PD=1, Control=0)
sample_data(gene.ps)$collection_method <-
```

```
                dplyr::recode(sample_data(gene.ps)$collection_method, swab=1, `OMNIgene GUT`=0)
sample_data(gene.ps)$seqs_scaled <- scale(sample_data(gene.ps)$total_sequences)

sample_data(path.ps)$Case_status <-
                dplyr::recode(sample_data(path.ps)$Case_status, PD=1, Control=0)
sample_data(path.ps)$collection_method <-
                dplyr::recode(sample_data(path.ps)$collection_method, swab=1, `OMNIgene GUT`=0)
sample_data(path.ps)$seqs_scaled <- scale(sample_data(path.ps)$total_sequences)

# perform differential abundance analysis using ANCOM-BC with abundance data
ancom.gene <- ANCOMBC.plus(ps=gene.ps,
                           formula="Case_status + collection_method + seqs_scaled",
                           p_adj_method="BH",
                           zero_cut=0.95)

ancom.path <- ANCOMBC.plus(ps=path.ps,
                           formula="Case_status + collection_method + seqs_scaled",
                           p_adj_method="BH",
                           zero_cut=0.95)

# prep temporary directory for MaAsLin2 output
system('
if [ ! -d "temp_directory" ]
then
  mkdir temp_directory
fi
')

# perform differential abundance analysis using linear regression
# with log2 transformed relative abundances
suppress(
lm.gene <- MaAsLin2.plus(ps=transform_sample_counts(gene.ps, function(x){x/sum(x)}),
                         output='temp_directory',
                         metadata=c('Case_status','collection_method','seqs_scaled'),
                         min_prevalence=0.05,
                         normalization='NONE',
                         max_significance=0.05,
                         standardize=FALSE,
                         plot_heatmap=FALSE,
                         plot_scatter=FALSE)
)

suppress(
lm.path <- MaAsLin2.plus(ps=transform_sample_counts(path.ps, function(x){x/sum(x)}),
                         output='temp_directory',
                         metadata=c('Case_status','collection_method','seqs_scaled'),
                         min_prevalence=0.05,
                         normalization='NONE',
                         max_significance=0.05,
                         standardize=FALSE,
                         plot_heatmap=FALSE,
                         plot_scatter=FALSE)
)
```

```r
# remove temporary output directory
system('rm -r temp_directory')

# initialize workbook
wb <- createWorkbook()

# coalesce results for KO groups
res.summ <- data.frame(Variable=lm.gene$result.summary$Variable,
                       `KEGG ID`=sapply(strsplit(lm.gene$result.summary$Feature, ": "),
                                        function(x){x[1]}),
                       `KEGG ortholog group`=sapply(strsplit(lm.gene$result.summary$Feature, ": "),
                                                    function(x){x[2]}),
                       `N PD`=lm.gene$result.summary$N1,
                       `N NHC`=lm.gene$result.summary$N2,
                       space_1='',
                       `RA in PD`=lm.gene$result.summary$Mean1,
                       `RA in NHC`=lm.gene$result.summary$Mean2,
                       lm.gene$result.summary[,c('Beta','SE','P','FDR','FC')],
                       `FC lower`=lm.gene$result.summary$FC_lower,
                       `FC upper`=lm.gene$result.summary$FC_upper,
                       space_2='', check.names=FALSE)
res.summ <- merge(res.summ,
                  data.frame(Variable=ancom.gene$result.summary$Variable,
                      `KEGG ID`=sapply(strsplit(ancom.gene$result.summary$Feature, ": "),
                                       function(x){x[1]}),
                      `KEGG ortholog group`=sapply(strsplit(ancom.gene$result.summary$Feature, ": "),
                                                   function(x){x[2]}),
                      `N PD`=ancom.gene$result.summary$N1,
                      `N NHC`=ancom.gene$result.summary$N2,
                      `BC-OA in PD`=ancom.gene$result.summary$Mean1,
                      `BC-OA in NHC`=ancom.gene$result.summary$Mean2,
                      ancom.gene$result.summary[,c('Beta','SE','P','FDR','FC')],
                      `FC lower`=ancom.gene$result.summary$FC_lower,
                      `FC upper`=ancom.gene$result.summary$FC_upper,
                      check.names=FALSE),
                  by=c('Variable','KEGG ID','KEGG ortholog group','N PD','N NHC'),
                  suffix=c('_m','_a'), all=TRUE, sort=FALSE)
res.summ <- res.summ[res.summ$Variable=='Case_status',-1]
res.summ <- rbind(data.frame(`KEGG ID`='', `KEGG ortholog group`='',
                             `N PD`='', `N NHC`='', space_1='',
                             `RA in PD`='MaAsLin2 results', `RA in NHC`='',
                             Beta_m='', SE_m='', P_m='', FDR_m='',
                             FC_m='', `FC lower_m`='', `FC upper_m`='', space_2='',
                             `BC-OA in PD`='ANCOM-BC results', `BC-OA in NHC`='',
                             Beta_a='', SE_a='', P_a='', FDR_a='',
                             FC_a='', `FC lower_a`='', `FC upper_a`='',
                             check.names=FALSE),
                  data.frame(`KEGG ID`='KEGG ID', `KEGG ortholog group`='KEGG ortholog group',
                             `N PD`='N PD', `N NHC`='N NHC', space_1='',
                             `RA in PD`='RA in PD', `RA in NHC`='RA in NHC', Beta_m='Beta',
                             SE_m='SE', P_m='P', FDR_m='FDR', FC_m='FC',
                             `FC lower_m`='FC lower', `FC upper_m`='FC upper', space_2='',
                             `BC-OA in PD`='BC-OA in PD', `BC-OA in NHC`='BC-OA in NHC',
                             Beta_a='Beta', SE_a='SE', P_a='P', FDR_a='FDR', FC_a='FC',
```

```
                             `FC lower_a`='FC lower', `FC upper_a`='FC upper',
                             check.names=FALSE),
                  res.summ)
res.summ[3:(nrow(res.summ)-1),
         c(grep('MaAsLin2', res.summ[1,]):(grep('space_2', colnames(res.summ))-1),
           grep('ANCOM', res.summ[1,]):ncol(res.summ))][
         is.na(res.summ[3:(nrow(res.summ)-1),
                        c(grep('MaAsLin2', res.summ[1,]):(grep('space_2', colnames(res.summ))-1),
                          grep('ANCOM', res.summ[1,]):ncol(res.summ))])] <- 'NT'

# add results for KO groups and format
addWorksheet(wb, 'KO group results')
writeData(wb, 'KO group results', res.summ, keepNA=FALSE, colNames=FALSE)
setColWidths(wb, 'KO group results', cols=seq_len(ncol(res.summ)),
             widths=c(10, 82, rep(11,2), 2, rep(11,9), 2, rep(11,9))) ### format cells
mergeCells(wb, 'KO group results',
           cols=grep('MaAsLin2', res.summ[1,]):(grep('space_2', colnames(res.summ))-1), rows=1)
mergeCells(wb, 'KO group results',
           cols=grep('ANCOM', res.summ[1,]):ncol(res.summ), rows=1)
addStyle(wb, 'KO group results', cols=seq_len(ncol(res.summ)),
         rows=1:2, style=bold, stack=TRUE, gridExpand=TRUE) ### font
addStyle(wb, 'KO group results', cols=seq_len(ncol(res.summ)),
         rows=c(1,3,(nrow(res.summ)+1)), ### borders
         gridExpand=TRUE, style=horizontal_border_med, stack=TRUE)
addStyle(wb, 'KO group results',
         cols=grep('MaAsLin2', res.summ[1,]):(grep('space_2', colnames(res.summ))-1), rows=2,
         style=horizontal_border_thin, stack=TRUE)
addStyle(wb, 'KO group results', cols=grep('ANCOM', res.summ[1,]):ncol(res.summ),
         rows=2, style=horizontal_border_thin, stack=TRUE)
# convert numbers from strings back to numbers
# Note: this convertNum function below may take too long to run
# on a standard machine, consider skipping or running over night
###convertNum(res.summ, wb, 'KO group results', FALSE)

# coalesce results for pathways
res.summ <- data.frame(Variable=lm.path$result.summary$Variable,
                       `MetaCyc ID`=sapply(strsplit(lm.path$result.summary$Feature, ": "),
                                           function(x){x[1]}),
                       `Pathway`=sapply(strsplit(lm.path$result.summary$Feature, ": "),
                                        function(x){x[2]}),
                       `N PD`=lm.path$result.summary$N1,
                       `N NHC`=lm.path$result.summary$N2,
                       space_1='',
                       `RA in PD`=lm.path$result.summary$Mean1,
                       `RA in NHC`=lm.path$result.summary$Mean2,
                       lm.path$result.summary[,c('Beta','SE','P','FDR','FC')],
                       `FC lower`=lm.path$result.summary$FC_lower,
                       `FC upper`=lm.path$result.summary$FC_upper,
                       space_2='', check.names=FALSE)
res.summ <- merge(res.summ,
                  data.frame(Variable=ancom.path$result.summary$Variable,
                             `MetaCyc ID`=sapply(strsplit(ancom.path$result.summary$Feature, ": "),
                                                 function(x){x[1]}),
                             `Pathway`=sapply(strsplit(ancom.path$result.summary$Feature, ": "),
```

```r
                                                function(x){x[2]}),
                                `N PD`=ancom.path$result.summary$N1,
                                `N NHC`=ancom.path$result.summary$N2,
                                `BC-OA in PD`=ancom.path$result.summary$Mean1,
                                `BC-OA in NHC`=ancom.path$result.summary$Mean2,
                                ancom.path$result.summary[,c('Beta','SE','P','FDR','FC')],
                                `FC lower`=ancom.path$result.summary$FC_lower,
                                `FC upper`=ancom.path$result.summary$FC_upper,
                                check.names=FALSE),
                  by=c('Variable','MetaCyc ID','Pathway','N PD','N NHC'),
                  suffix=c('_m','_a'), all=TRUE, sort=FALSE)
res.summ <- res.summ[res.summ$Variable=='Case_status',-1]
res.summ <- rbind(data.frame(`MetaCyc ID`='', `Pathway`='',
                                `N PD`='', `N NHC`='', space_1='',
                                `RA in PD`='MaAsLin2 results', `RA in NHC`='',
                                Beta_m='', SE_m='', P_m='', FDR_m='',
                                FC_m='', `FC lower_m`='', `FC upper_m`='', space_2='',
                                `BC-OA in PD`='ANCOM-BC results', `BC-OA in NHC`='',
                                Beta_a='', SE_a='', P_a='', FDR_a='',
                                FC_a='', `FC lower_a`='', `FC upper_a`='',
                                check.names=FALSE),
                  data.frame(`MetaCyc ID`='MetaCyc ID', `Pathway`='Pathway',
                                `N PD`='N PD', `N NHC`='N NHC', space_1='',
                                `RA in PD`='RA in PD', `RA in NHC`='RA in NHC',
                                 Beta_m='Beta', SE_m='SE', P_m='P', FDR_m='FDR', FC_m='FC',
                                `FC lower_m`='FC lower', `FC upper_m`='FC upper', space_2='',
                                `BC-OA in PD`='BC-OA in PD', `BC-OA in NHC`='BC-OA in NHC',
                                Beta_a='Beta', SE_a='SE', P_a='P', FDR_a='FDR', FC_a='FC',
                                `FC lower_a`='FC lower', `FC upper_a`='FC upper',
                                check.names=FALSE),
                  res.summ)
res.summ[3:(nrow(res.summ)-1),
         c(grep('MaAsLin2', res.summ[1,]):(grep('space_2', colnames(res.summ))-1),
           grep('ANCOM', res.summ[1,]):ncol(res.summ))][
         is.na(res.summ[3:(nrow(res.summ)-1),
                        c(grep('MaAsLin2', res.summ[1,]):(grep('space_2', colnames(res.summ))-1),
                          grep('ANCOM', res.summ[1,]):ncol(res.summ))])] <- 'NT'

# add results for pathways and format
addWorksheet(wb, 'Pathway results')
writeData(wb, 'Pathway results', res.summ, keepNA=FALSE, colNames=FALSE)
setColWidths(wb, 'Pathway results', cols=seq_len(ncol(res.summ)),
             widths=c(10, 82, rep(11,2), 2, rep(11,9), 2, rep(11,9))) ### format cells
mergeCells(wb, 'Pathway results',
           cols=grep('MaAsLin2', res.summ[1,]):(grep('space_2', colnames(res.summ))-1), rows=1)
mergeCells(wb, 'Pathway results',
           cols=grep('ANCOM', res.summ[1,]):ncol(res.summ), rows=1)
addStyle(wb, 'Pathway results', cols=seq_len(ncol(res.summ)),
         rows=1:2, style=bold, stack=TRUE, gridExpand=TRUE) ### font
addStyle(wb, 'Pathway results', cols=seq_len(ncol(res.summ)),
         rows=c(1,3,(nrow(res.summ)+1)), ### borders
         gridExpand=TRUE, style=horizontal_border_med, stack=TRUE)
addStyle(wb, 'Pathway results',
         cols=grep('MaAsLin2', res.summ[1,]):(grep('space_2', colnames(res.summ))-1), rows=2,
```

```r
         style=horizontal_border_thin, stack=TRUE)
addStyle(wb, 'Pathway results', cols=grep('ANCOM', res.summ[1,]):ncol(res.summ),
         rows=2, style=horizontal_border_thin, stack=TRUE)
# convert numbers from strings back to numbers
convertNum(res.summ, wb, 'Pathway results', FALSE)

# save workbook
saveWorkbook(wb,
    'PDShotgunAnalysis_out/5.Gene_pathway_associations/MaAsLin2_ANCOMBC_MWAS_PDvsNHC.xlsx',
    overwrite=TRUE)
```

Sporulation KO group association with constipation

Given that spore forming bacteria stimulate gut motility, and our data suggest a global depletion of the sporulation KO groups, we speculated, and tested the hypothesis that constipation, a common symptom of PD, may be related to the depletion of spore forming bacteria.

- To determine if broad reduction in sporulation KO groups is associated with constipation, tested association of PD-associated sporulation KO groups with constipation in PD and NHC groups by collapsing the relative abundances of differentially abundant sporulation KO groups (FDR < 0.05 in both MaAsLin2 and ANCOM-BC; 27 in total), and testing for association with constipation in PD and NHC subjects separately using linear regression with log2 transformed relative abundances (as done in MaAsLin2).
- To determine if constipation was driving the PD association signal observed for sporulation KO groups, tested for association of PD with the collapsed sporulation KO groups while including constipation in the model.

```r
#### SPORULATION KO GROUPS & CONSTIPATION ####

# recode variable to get correct effect direction
sample_data(gene.ps)$Constipation <- dplyr::recode(sample_data(gene.ps)$Constipation, Y=1, N=0)

# define target KO groups
target_ko <- intersect(ancom.gene$result.summary$Feature[ancom.gene$result.summary$FDR < 0.05 &
                                                         !is.na(ancom.gene$result.summary$FDR)],
                       lm.gene$result.summary$Feature[lm.gene$result.summary$FDR < 0.05 &
                                                        !is.na(lm.gene$result.summary$FDR)])
target_ko <- target_ko[grep('sporulation', target_ko)]

# collapse KO group relative abundances into one group
ra.mod <- data.frame(otu_table(transform_sample_counts(gene.ps, function(x){x/sum(x)})),
                     check.names=FALSE)
ra.mod <- data.frame(`Sporulation KOs`=rowSums(ra.mod[,colnames(ra.mod) %in% target_ko]),
                     ra.mod[,!(colnames(ra.mod) %in% target_ko)], check.names=FALSE)

# log2 transform
log2.ra <- data.frame(apply(ra.mod, 2, log2.trans), check.names=FALSE)

# begin result data.frame
mod.results <- data.frame(`Subject group`="",
              Beta1="Results for constipation",
              SE1="",
              P1="",
              FC1="",
```

```r
                Beta2="Results for case status",
                SE2="",
                P2="",
                FC2="",
                check.names=FALSE)
mod.results <- rbind(mod.results,
              data.frame(`Subject group`="Subject group",
                Beta1="Beta",
                SE1="SE",
                P1="P",
                FC1="FC",
                Beta2="Beta",
                SE2="SE",
                P2="P",
                FC2="FC",
                check.names=FALSE))

### PD ###

# subset for only PD
ra.sub <- ra.mod[rownames(ra.mod) %in%
                 sample_names(subset_samples(gene.ps, Case_status == 1)),]
log2.sub <- log2.ra[rownames(log2.ra) %in%
                   sample_names(subset_samples(gene.ps, Case_status == 1)),]
ps.sub <- subset_samples(gene.ps, Case_status == 1)

# perform linear regression
ra.lm <- lm(log2.sub$`Sporulation KOs` ~ Constipation + collection_method + seqs_scaled,
            data=data.frame(sample_data(ps.sub)))

# coalesce results
mod.results <- rbind(mod.results,
              data.frame(`Subject group`="PD",
                Beta1=round(summary(ra.lm)$coefficients[2,1],2),
                SE1=round(summary(ra.lm)$coefficients[2,2],2),
                P1=formatC(summary(ra.lm)$coefficients[2,4],format='e',digits=1),
                FC1=round(2^summary(ra.lm)$coefficients[2,1],2),
                Beta2="-",
                SE2="-",
                P2="-",
                FC2="-",
                check.names=FALSE))

### NHC ###

# subset for only NHC
ra.sub <- ra.mod[rownames(ra.mod) %in%
                 sample_names(subset_samples(gene.ps, Case_status == 0)),]
log2.sub <- log2.ra[rownames(log2.ra) %in%
                   sample_names(subset_samples(gene.ps, Case_status == 0)),]
ps.sub <- subset_samples(gene.ps, Case_status == 0)

# perform linear regression
ra.lm <- lm(log2.sub$`Sporulation KOs` ~ Constipation + collection_method + seqs_scaled,
```

```
             data=data.frame(sample_data(ps.sub)))

# coalesce results
mod.results <- rbind(mod.results,
              data.frame(`Subject group`="NHC",
              Beta1=round(summary(ra.lm)$coefficients[2,1],2),
              SE1=round(summary(ra.lm)$coefficients[2,2],2),
              P1=formatC(summary(ra.lm)$coefficients[2,4],format='e',digits=1),
              FC1=round(2^summary(ra.lm)$coefficients[2,1],2),
              Beta2="-",
              SE2="-",
              P2="-",
              FC2="-",
              check.names=FALSE))

### PD and NHC ###

# perform linear regression
ra.lm <- lm(log2.ra$`Sporulation KOs` ~
             Constipation + Case_status + collection_method + seqs_scaled,
             data=data.frame(sample_data(gene.ps)))

# coalesce results
mod.results <- rbind(mod.results,
               data.frame(`Subject group`="PD and NHC",
              Beta1=round(summary(ra.lm)$coefficients[2,1],2),
              SE1=round(summary(ra.lm)$coefficients[2,2],2),
              P1=formatC(summary(ra.lm)$coefficients[2,4],format='e',digits=1),
              FC1=round(2^summary(ra.lm)$coefficients[2,1],2),
              Beta2=round(summary(ra.lm)$coefficients[3,1],2),
              SE2=round(summary(ra.lm)$coefficients[3,2],2),
              P2=formatC(summary(ra.lm)$coefficients[3,4],format='e',digits=1),
              FC2=round(2^summary(ra.lm)$coefficients[3,1],2),
              check.names=FALSE))

# write results
# create workbook
wb <- createWorkbook()
# add worksheet, write data, and format output
addWorksheet(wb, 'Sporulation KO groups')
writeData(wb, 'Sporulation KO groups', mod.results, keepNA=TRUE, colNames=FALSE)
setColWidths(wb, 'Sporulation KO groups', cols=seq_len(ncol(mod.results)),
             widths=c(20, rep(10,4), rep(10,4))) ### format cells
addStyle(wb, 'Sporulation KO groups', cols=seq_len(ncol(mod.results)),
         rows=1:(nrow(mod.results)+1), gridExpand=TRUE, style=center, stack=TRUE)
addStyle(wb, 'Sporulation KO groups', cols=seq_len(ncol(mod.results)),
         rows=1:2, gridExpand=TRUE, style=bold, stack=TRUE) ### font
addStyle(wb, 'Sporulation KO groups', cols=seq_len(ncol(mod.results)),
         rows=c(1,3,(nrow(mod.results)+1)), ### borders
         gridExpand=TRUE, style=horizontal_border_med, stack=TRUE)
# convert numbers from strings back to numbers
convertNum(mod.results, wb, 'Sporulation KO groups', FALSE)
# save workbook
saveWorkbook(wb,
```

```
    'PDShotgunAnalysis_out/5.Gene_pathway_associations/Sporulation_KOs_constipation.xlsx',
    overwrite=TRUE)
```

Gene family/pathway boxplots with fold changes from MWAS

To summarize differential abundance analysis results of KO groups and pathways, plotted the relative abundances and fold changes of select KO groups and pathways that were chosen based on relevance to current PD literature.

```
#### PD-ASSOCIATED KO GROUPS & PATHWAY  ####
#### DISTRIBUTIONS & FOLD CHANGES       ####

# grab relative abundances and bias-corrected abundances of KO groups and pathways
ra.gene <- ra.mod
ba.gene <- data.frame(otu_table(ancom.gene$bias.corrected.ps), check.names=FALSE)
ra.path <- data.frame(otu_table(transform_sample_counts(path.ps, function(x){x/sum(x)})),
                      check.names=FALSE)
ba.path <- data.frame(otu_table(ancom.path$bias.corrected.ps), check.names=FALSE)

# extract only IDs
colnames(ra.gene) <- sapply(strsplit(colnames(ra.gene), ": "), function(x){x[1]})
colnames(ba.gene) <- sapply(strsplit(colnames(ba.gene), ": "), function(x){x[1]})
colnames(ra.path) <- sapply(strsplit(colnames(ra.path), ": "), function(x){x[1]})
colnames(ba.path) <- sapply(strsplit(colnames(ba.path), ": "), function(x){x[1]})

### pull out plotting data for selected KOs and pathways
### in order of common categories and proteins

## elevated immunogenic bacterial components
# LPS/lipid A
targets <- c('K02535','K09949','K04744','KDO-NAGLIPASYN-PWY','PWY0-881','PWY-6285','ECASYN-PWY')

lps.ra.data <- cbind(ra.gene[,colnames(ra.gene) %in% targets, FALSE],
                     ra.path[,colnames(ra.path) %in% targets, FALSE])
lps.ra.fc.data <- rbind(
          lm.gene$result.summary[sapply(strsplit(lm.gene$result.summary$Feature, ": "),
                                        function(x){x[1]}) %in% targets &
                                 lm.gene$result.summary$Variable == 'Case_status',
                                 c('FC','FC_lower','FC_upper')],
          lm.path$result.summary[sapply(strsplit(lm.path$result.summary$Feature, ": "),
                                        function(x){x[1]}) %in% targets &
                                 lm.path$result.summary$Variable == 'Case_status',
                                  c('FC','FC_lower','FC_upper')])
rownames(lps.ra.fc.data) <- sapply(strsplit(rownames(lps.ra.fc.data), ": "), function(x){x[1]})
lps.ra.fc.data <- lps.ra.fc.data[order(lps.ra.fc.data$FC, decreasing=FALSE),]
lps.ra.data <- lps.ra.data[,rownames(lps.ra.fc.data), FALSE]
lps.ra.fc.data <- data.frame(sub_group='LPS/ lipid A',
                             plot='Absolute fold change with 95%CI',
                             line='MaAsLin2',
                             variable=rownames(lps.ra.fc.data), lps.ra.fc.data)
lps.ra.data <- data.frame(sub_group='LPS/ lipid A', plot='log2(Relative abundances)',
                          Case_status=sample_data(gene.ps)$Case_status, lps.ra.data)

lps.ba.data <- cbind(ba.gene[,colnames(ba.gene) %in% targets, FALSE],
```

```
                    ba.path[,colnames(ba.path) %in% targets, FALSE])
lps.ba.fc.data <- rbind(
          ancom.gene$result.summary[sapply(strsplit(ancom.gene$result.summary$Feature, ": "),
                                           function(x){x[1]}) %in% targets &
                                    ancom.gene$result.summary$Variable == 'Case_status',
                                    c('FC','FC_lower','FC_upper')],
          ancom.path$result.summary[sapply(strsplit(ancom.path$result.summary$Feature, ": "),
                                           function(x){x[1]}) %in% targets &
                                    ancom.path$result.summary$Variable == 'Case_status',
                                    c('FC','FC_lower','FC_upper')])
rownames(lps.ba.fc.data) <- sapply(strsplit(rownames(lps.ba.fc.data), ": "), function(x){x[1]})
lps.ba.fc.data <- lps.ba.fc.data[rownames(lps.ra.fc.data),, FALSE]
lps.ba.data <- lps.ba.data[,rownames(lps.ra.fc.data), FALSE]
lps.ba.fc.data <- data.frame(sub_group='LPS/ lipid A',
                             plot='Absolute fold change with 95%CI',
                             line='ANCOM-BC',
                             variable=rownames(lps.ba.fc.data), lps.ba.fc.data)
lps.ba.data <- data.frame(sub_group='LPS/ lipid A', plot='log(Bias-corrected abundances)',
                          Case_status=sample_data(gene.ps)$Case_status, lps.ba.data)

# LTA
targets <- c('K19005','K03739')

lta.ra.data <- cbind(ra.gene[,colnames(ra.gene) %in% targets, FALSE],
                    ra.path[,colnames(ra.path) %in% targets, FALSE])
lta.ra.fc.data <- rbind(
          lm.gene$result.summary[sapply(strsplit(lm.gene$result.summary$Feature, ": "),
                                        function(x){x[1]}) %in% targets &
                                 lm.gene$result.summary$Variable == 'Case_status',
                                 c('FC','FC_lower','FC_upper')],
          lm.path$result.summary[sapply(strsplit(lm.path$result.summary$Feature, ": "),
                                        function(x){x[1]}) %in% targets &
                                 lm.path$result.summary$Variable == 'Case_status',
                                  c('FC','FC_lower','FC_upper')])
rownames(lta.ra.fc.data) <- sapply(strsplit(rownames(lta.ra.fc.data), ": "), function(x){x[1]})
lta.ra.fc.data <- lta.ra.fc.data[order(lta.ra.fc.data$FC, decreasing=FALSE),]
lta.ra.data <- lta.ra.data[,rownames(lta.ra.fc.data), FALSE]
lta.ra.fc.data <- data.frame(sub_group='LTA',
                             plot='Absolute fold change with 95%CI',
                             line='MaAsLin2',
                             variable=rownames(lta.ra.fc.data), lta.ra.fc.data)
lta.ra.data <- data.frame(sub_group='LTA', plot='log2(Relative abundances)',
                          Case_status=sample_data(gene.ps)$Case_status, lta.ra.data)

lta.ba.data <- cbind(ba.gene[,colnames(ba.gene) %in% targets, FALSE],
                    ba.path[,colnames(ba.path) %in% targets, FALSE])
lta.ba.fc.data <- rbind(
          ancom.gene$result.summary[sapply(strsplit(ancom.gene$result.summary$Feature, ": "),
                                           function(x){x[1]}) %in% targets &
                                    ancom.gene$result.summary$Variable == 'Case_status',
                                    c('FC','FC_lower','FC_upper')],
          ancom.path$result.summary[sapply(strsplit(ancom.path$result.summary$Feature, ": "),
                                           function(x){x[1]}) %in% targets &
                                    ancom.path$result.summary$Variable == 'Case_status',
```

```r
                                    c('FC','FC_lower','FC_upper')])
rownames(lta.ba.fc.data) <- sapply(strsplit(rownames(lta.ba.fc.data), ": "), function(x){x[1]})
lta.ba.fc.data <- lta.ba.fc.data[rownames(lta.ra.fc.data),, FALSE]
lta.ba.data <- lta.ba.data[,rownames(lta.ra.fc.data), FALSE]
lta.ba.fc.data <- data.frame(sub_group='LTA',
                             plot='Absolute fold change with 95%CI',
                             line='ANCOM-BC',
                             variable=rownames(lta.ba.fc.data), lta.ba.fc.data)
lta.ba.data <- data.frame(sub_group='LTA', plot='log(Bias-corrected abundances)',
                          Case_status=sample_data(gene.ps)$Case_status, lta.ba.data)

# BLP
targets <- c('K06078')

blp.ra.data <- cbind(ra.gene[,colnames(ra.gene) %in% targets, FALSE],
                     ra.path[,colnames(ra.path) %in% targets, FALSE])
blp.ra.fc.data <- rbind(
          lm.gene$result.summary[sapply(strsplit(lm.gene$result.summary$Feature, ": "),
                                        function(x){x[1]}) %in% targets &
                                 lm.gene$result.summary$Variable == 'Case_status',
                                 c('FC','FC_lower','FC_upper')],
          lm.path$result.summary[sapply(strsplit(lm.path$result.summary$Feature, ": "),
                                        function(x){x[1]}) %in% targets &
                                 lm.path$result.summary$Variable == 'Case_status',
                                  c('FC','FC_lower','FC_upper')])
rownames(blp.ra.fc.data) <- sapply(strsplit(rownames(blp.ra.fc.data), ": "), function(x){x[1]})
blp.ra.fc.data <- blp.ra.fc.data[order(blp.ra.fc.data$FC, decreasing=FALSE),]
blp.ra.data <- blp.ra.data[,rownames(blp.ra.fc.data), FALSE]
blp.ra.fc.data <- data.frame(sub_group='BLP',
                             plot='Absolute fold change with 95%CI',
                             line='MaAsLin2',
                             variable=rownames(blp.ra.fc.data), blp.ra.fc.data)
blp.ra.data <- data.frame(sub_group='BLP', plot='log2(Relative abundances)',
                          Case_status=sample_data(gene.ps)$Case_status, blp.ra.data)

blp.ba.data <- cbind(ba.gene[,colnames(ba.gene) %in% targets, FALSE],
                     ba.path[,colnames(ba.path) %in% targets, FALSE])
blp.ba.fc.data <- rbind(
          ancom.gene$result.summary[sapply(strsplit(ancom.gene$result.summary$Feature, ": "),
                                           function(x){x[1]}) %in% targets &
                                    ancom.gene$result.summary$Variable == 'Case_status',
                                    c('FC','FC_lower','FC_upper')],
          ancom.path$result.summary[sapply(strsplit(ancom.path$result.summary$Feature, ": "),
                                           function(x){x[1]}) %in% targets &
                                    ancom.path$result.summary$Variable == 'Case_status',
                                    c('FC','FC_lower','FC_upper')])
rownames(blp.ba.fc.data) <- sapply(strsplit(rownames(blp.ba.fc.data), ": "), function(x){x[1]})
blp.ba.fc.data <- blp.ba.fc.data[rownames(blp.ra.fc.data),, FALSE]
blp.ba.data <- blp.ba.data[,rownames(blp.ra.fc.data), FALSE]
blp.ba.fc.data <- data.frame(sub_group='BLP',
                             plot='Absolute fold change with 95%CI',
                             line='ANCOM-BC',
                             variable=rownames(blp.ba.fc.data), blp.ba.fc.data)
blp.ba.data <- data.frame(sub_group='BLP', plot='log(Bias-corrected abundances)',
```

```
                          Case_status=sample_data(gene.ps)$Case_status, blp.ba.data)

# combine
fc.plot.data <- data.frame(group='Elevated immunogenic bacterial components',
                           rbind(lps.ra.fc.data, lta.ra.fc.data, blp.ra.fc.data,
                                 lps.ba.fc.data, lta.ba.fc.data, blp.ba.fc.data))
ab.plot.data <- merge(
                 data.frame(group='Elevated immunogenic bacterial components',
                            merge(lps.ra.data, merge(lta.ra.data, blp.ra.data, all=TRUE, sort=FALSE),
                                  all=TRUE, sort=FALSE)),
                 data.frame(group='Elevated immunogenic bacterial components',
                            merge(lps.ba.data, merge(lta.ba.data, blp.ba.data, all=TRUE, sort=FALSE),
                                  all=TRUE, sort=FALSE)),
                      all=TRUE,sort=FALSE)
colnames(ab.plot.data) <- gsub('\\.','-',colnames(ab.plot.data))

## reduced polysaccharide metabolism and loss of SCFA
targets <- c('K17236','K17234','K16213','K00702','PWY-7456','PWY-6527','PWY-7237',
             'PWY-7242','GALACTUROCAT-PWY','GLUCUROCAT-PWY','GALACT-GLUCUROCAT-PWY')

pbm.ra.data <- cbind(ra.gene[,colnames(ra.gene) %in% targets, FALSE],
                     ra.path[,colnames(ra.path) %in% targets, FALSE])
pbm.ra.fc.data <- rbind(
          lm.gene$result.summary[sapply(strsplit(lm.gene$result.summary$Feature, ": "),
                                        function(x){x[1]}) %in% targets &
                                 lm.gene$result.summary$Variable == 'Case_status',
                                 c('FC','FC_lower','FC_upper')],
          lm.path$result.summary[sapply(strsplit(lm.path$result.summary$Feature, ": "),
                                        function(x){x[1]}) %in% targets &
                                 lm.path$result.summary$Variable == 'Case_status',
                                  c('FC','FC_lower','FC_upper')])
rownames(pbm.ra.fc.data) <- sapply(strsplit(rownames(pbm.ra.fc.data), ": "), function(x){x[1]})
pbm.ra.fc.data <- pbm.ra.fc.data[order(pbm.ra.fc.data$FC, decreasing=TRUE),]
pbm.ra.data <- pbm.ra.data[,rownames(pbm.ra.fc.data), FALSE]
pbm.ra.fc.data <- data.frame(sub_group='---',
                             plot='Absolute fold change with 95%CI',
                             line='MaAsLin2',
                             variable=rownames(pbm.ra.fc.data), pbm.ra.fc.data)
pbm.ra.data <- data.frame(sub_group='---', plot='log2(Relative abundances)',
                          Case_status=sample_data(gene.ps)$Case_status, pbm.ra.data)

pbm.ba.data <- cbind(ba.gene[,colnames(ba.gene) %in% targets, FALSE],
                     ba.path[,colnames(ba.path) %in% targets, FALSE])
pbm.ba.fc.data <- rbind(
          ancom.gene$result.summary[sapply(strsplit(ancom.gene$result.summary$Feature, ": "),
                                           function(x){x[1]}) %in% targets &
                                    ancom.gene$result.summary$Variable == 'Case_status',
                                    c('FC','FC_lower','FC_upper')],
          ancom.path$result.summary[sapply(strsplit(ancom.path$result.summary$Feature, ": "),
                                           function(x){x[1]}) %in% targets &
                                    ancom.path$result.summary$Variable == 'Case_status',
                                    c('FC','FC_lower','FC_upper')])
rownames(pbm.ba.fc.data) <- sapply(strsplit(rownames(pbm.ba.fc.data), ": "), function(x){x[1]})
pbm.ba.fc.data <- pbm.ba.fc.data[rownames(pbm.ra.fc.data),, FALSE]
```

```
pbm.ba.data <- pbm.ba.data[,rownames(pbm.ra.fc.data), FALSE]
pbm.ba.fc.data <- data.frame(sub_group='---',
                             plot='Absolute fold change with 95%CI',
                             line='ANCOM-BC',
                             variable=rownames(pbm.ba.fc.data), pbm.ba.fc.data)
pbm.ba.data <- data.frame(sub_group='---', plot='log(Bias-corrected abundances)',
                          Case_status=sample_data(gene.ps)$Case_status, pbm.ba.data)

# combine
fc.plot.data <- rbind(fc.plot.data,
          data.frame(group='Reduced plant-based polysaccharide degradation and SCFA production',
                     rbind(pbm.ra.fc.data, pbm.ba.fc.data)))
ab.plot.data <- merge(ab.plot.data,
          merge(data.frame(group='Reduced plant-based polysaccharide degradation and SCFA production',
                           pbm.ra.data),
                data.frame(group='Reduced plant-based polysaccharide degradation and SCFA production',
                           pbm.ba.data),
                all=TRUE, sort=FALSE),
      all=TRUE, sort=FALSE)
colnames(ab.plot.data) <- gsub('\\.','-',colnames(ab.plot.data))

## elevated proteolytic pathways
targets <- c('ORNARGDEG-PWY','ORNDEG-PWY','THREOCAT-PWY')

pdp.ra.data <- cbind(ra.gene[,colnames(ra.gene) %in% targets, FALSE],
                     ra.path[,colnames(ra.path) %in% targets, FALSE])
pdp.ra.fc.data <- rbind(
          lm.gene$result.summary[sapply(strsplit(lm.gene$result.summary$Feature, ": "),
                                        function(x){x[1]}) %in% targets &
                                 lm.gene$result.summary$Variable == 'Case_status',
                                 c('FC','FC_lower','FC_upper')],
          lm.path$result.summary[sapply(strsplit(lm.path$result.summary$Feature, ": "),
                                        function(x){x[1]}) %in% targets &
                                 lm.path$result.summary$Variable == 'Case_status',
                                  c('FC','FC_lower','FC_upper')])
rownames(pdp.ra.fc.data) <- sapply(strsplit(rownames(pdp.ra.fc.data), ": "), function(x){x[1]})
pdp.ra.fc.data <- pdp.ra.fc.data[order(pdp.ra.fc.data$FC, decreasing=FALSE),]
pdp.ra.data <- pdp.ra.data[,rownames(pdp.ra.fc.data), FALSE]
pdp.ra.fc.data <- data.frame(sub_group='---',
                             plot='Absolute fold change with 95%CI',
                             line='MaAsLin2',
                             variable=rownames(pdp.ra.fc.data), pdp.ra.fc.data)
pdp.ra.data <- data.frame(sub_group='---', plot='log2(Relative abundances)',
                          Case_status=sample_data(gene.ps)$Case_status, pdp.ra.data)

pdp.ba.data <- cbind(ba.gene[,colnames(ba.gene) %in% targets, FALSE],
                     ba.path[,colnames(ba.path) %in% targets, FALSE])
pdp.ba.fc.data <- rbind(
          ancom.gene$result.summary[sapply(strsplit(ancom.gene$result.summary$Feature, ": "),
                                           function(x){x[1]}) %in% targets &
                                    ancom.gene$result.summary$Variable == 'Case_status',
                                    c('FC','FC_lower','FC_upper')],
          ancom.path$result.summary[sapply(strsplit(ancom.path$result.summary$Feature, ": "),
                                           function(x){x[1]}) %in% targets &
```

```
                                        ancom.path$result.summary$Variable == 'Case_status',
                                        c('FC','FC_lower','FC_upper')])
rownames(pdp.ba.fc.data) <- sapply(strsplit(rownames(pdp.ba.fc.data), ": "), function(x){x[1]})
pdp.ba.fc.data <- pdp.ba.fc.data[rownames(pdp.ra.fc.data),, FALSE]
pdp.ba.data <- pdp.ba.data[,rownames(pdp.ra.fc.data), FALSE]
pdp.ba.fc.data <- data.frame(sub_group='---',
                             plot='Absolute fold change with 95%CI',
                             line='ANCOM-BC',
                             variable=rownames(pdp.ba.fc.data), pdp.ba.fc.data)
pdp.ba.data <- data.frame(sub_group='---', plot='log(Bias-corrected abundances)',
                          Case_status=sample_data(gene.ps)$Case_status, pdp.ba.data)

# combine
fc.plot.data <- rbind(fc.plot.data, data.frame(group='Increased protein degradation',
                                               rbind(pdp.ra.fc.data, pdp.ba.fc.data)))
ab.plot.data <- merge(ab.plot.data,
                      merge(data.frame(group='Increased protein degradation', pdp.ra.data),
                            data.frame(group='Increased protein degradation', pdp.ba.data),
                            all=TRUE, sort=FALSE),
                      all=TRUE, sort=FALSE)
colnames(ab.plot.data) <- gsub('\\.','-',colnames(ab.plot.data))

## dysregulated neuroactive signaling
# dopamine synthesis
targets <- c('COMPLETE-ARO-PWY')

dsp.ra.data <- cbind(ra.gene[,colnames(ra.gene) %in% targets, FALSE],
                     ra.path[,colnames(ra.path) %in% targets, FALSE])
dsp.ra.fc.data <- rbind(
          lm.gene$result.summary[sapply(strsplit(lm.gene$result.summary$Feature, ": "),
                                        function(x){x[1]}) %in% targets &
                                  lm.gene$result.summary$Variable == 'Case_status',
                                  c('FC','FC_lower','FC_upper')],
          lm.path$result.summary[sapply(strsplit(lm.path$result.summary$Feature, ": "),
                                        function(x){x[1]}) %in% targets &
                                  lm.path$result.summary$Variable == 'Case_status',
                                   c('FC','FC_lower','FC_upper')])
rownames(dsp.ra.fc.data) <- sapply(strsplit(rownames(dsp.ra.fc.data), ": "), function(x){x[1]})
dsp.ra.fc.data <- dsp.ra.fc.data[order(dsp.ra.fc.data$FC, decreasing=TRUE),]
dsp.ra.data <- dsp.ra.data[,rownames(dsp.ra.fc.data), FALSE]
dsp.ra.fc.data <- data.frame(sub_group='dopamine synthesis',
                             plot='Absolute fold change with 95%CI',
                             line='MaAsLin2',
                             variable=rownames(dsp.ra.fc.data), dsp.ra.fc.data)
dsp.ra.data <- data.frame(sub_group='dopamine synthesis', plot='log2(Relative abundances)',
                          Case_status=sample_data(gene.ps)$Case_status, dsp.ra.data)

dsp.ba.data <- cbind(ba.gene[,colnames(ba.gene) %in% targets, FALSE],
                     ba.path[,colnames(ba.path) %in% targets, FALSE])
dsp.ba.fc.data <- rbind(
          ancom.gene$result.summary[sapply(strsplit(ancom.gene$result.summary$Feature, ": "),
                                           function(x){x[1]}) %in% targets &
                                     ancom.gene$result.summary$Variable == 'Case_status',
                                     c('FC','FC_lower','FC_upper')],
```

```
          ancom.path$result.summary[sapply(strsplit(ancom.path$result.summary$Feature, ": "),
                                     function(x){x[1]}) %in% targets &
                              ancom.path$result.summary$Variable == 'Case_status',
                              c('FC','FC_lower','FC_upper')])
rownames(dsp.ba.fc.data) <- sapply(strsplit(rownames(dsp.ba.fc.data), ": "), function(x){x[1]})
dsp.ba.fc.data <- dsp.ba.fc.data[rownames(dsp.ra.fc.data),, FALSE]
dsp.ba.data <- dsp.ba.data[,rownames(dsp.ra.fc.data), FALSE]
dsp.ba.fc.data <- data.frame(sub_group='dopamine synthesis',
                             plot='Absolute fold change with 95%CI',
                             line='ANCOM-BC',
                             variable=rownames(dsp.ba.fc.data), dsp.ba.fc.data)
dsp.ba.data <- data.frame(sub_group='dopamine synthesis', plot='log(Bias-corrected abundances)',
                          Case_status=sample_data(gene.ps)$Case_status, dsp.ba.data)

# glutamate synthesis
targets <- c('K00266','PWY-5505')

gsp.ra.data <- cbind(ra.gene[,colnames(ra.gene) %in% targets, FALSE],
                     ra.path[,colnames(ra.path) %in% targets, FALSE])
gsp.ra.fc.data <- rbind(
          lm.gene$result.summary[sapply(strsplit(lm.gene$result.summary$Feature, ": "),
                                        function(x){x[1]}) %in% targets &
                                 lm.gene$result.summary$Variable == 'Case_status',
                                 c('FC','FC_lower','FC_upper')],
          lm.path$result.summary[sapply(strsplit(lm.path$result.summary$Feature, ": "),
                                        function(x){x[1]}) %in% targets &
                                 lm.path$result.summary$Variable == 'Case_status',
                                  c('FC','FC_lower','FC_upper')])
rownames(gsp.ra.fc.data) <- sapply(strsplit(rownames(gsp.ra.fc.data), ": "), function(x){x[1]})
gsp.ra.fc.data <- gsp.ra.fc.data[order(gsp.ra.fc.data$FC, decreasing=TRUE),]
gsp.ra.data <- gsp.ra.data[,rownames(gsp.ra.fc.data), FALSE]
gsp.ra.fc.data <- data.frame(sub_group='glutamate synthesis',
                             plot='Absolute fold change with 95%CI',
                             line='MaAsLin2',
                             variable=rownames(gsp.ra.fc.data), gsp.ra.fc.data)
gsp.ra.data <- data.frame(sub_group='glutamate synthesis', plot='log2(Relative abundances)',
                          Case_status=sample_data(gene.ps)$Case_status, gsp.ra.data)

gsp.ba.data <- cbind(ba.gene[,colnames(ba.gene) %in% targets, FALSE],
                     ba.path[,colnames(ba.path) %in% targets, FALSE])
gsp.ba.fc.data <- rbind(
          ancom.gene$result.summary[sapply(strsplit(ancom.gene$result.summary$Feature, ": "),
                                     function(x){x[1]}) %in% targets &
                              ancom.gene$result.summary$Variable == 'Case_status',
                              c('FC','FC_lower','FC_upper')],
          ancom.path$result.summary[sapply(strsplit(ancom.path$result.summary$Feature, ": "),
                                     function(x){x[1]}) %in% targets &
                              ancom.path$result.summary$Variable == 'Case_status',
                              c('FC','FC_lower','FC_upper')])
rownames(gsp.ba.fc.data) <- sapply(strsplit(rownames(gsp.ba.fc.data), ": "), function(x){x[1]})
gsp.ba.fc.data <- gsp.ba.fc.data[rownames(gsp.ra.fc.data),, FALSE]
gsp.ba.data <- gsp.ba.data[,rownames(gsp.ra.fc.data), FALSE]
gsp.ba.fc.data <- data.frame(sub_group='glutamate synthesis',
                             plot='Absolute fold change with 95%CI',
```

```
                               line='ANCOM-BC',
                               variable=rownames(gsp.ba.fc.data), gsp.ba.fc.data)
gsp.ba.data <- data.frame(sub_group='glutamate synthesis', plot='log(Bias-corrected abundances)',
                            Case_status=sample_data(gene.ps)$Case_status, gsp.ba.data)

# serotonin synthesis
targets <- c('Sporulation KOs','TRPSYN-PWY')

ssp.ra.data <- cbind(ra.gene[,colnames(ra.gene) %in% targets, FALSE],
                      ra.path[,colnames(ra.path) %in% targets, FALSE])
ssp.ra.fc.data <- rbind(data.frame(FC=2^(coef(lm(log2.ra$`Sporulation KOs` ~
                                                  Case_status + collection_method + seqs_scaled,
                                                  data=data.frame(sample_data(gene.ps))))[2]),
                                     FC_lower=2^(confint(lm(log2.ra$`Sporulation KOs` ~
                                                       Case_status + collection_method + seqs_scaled,
                                                       data=data.frame(sample_data(gene.ps))))[2,1]),
                                     FC_upper=2^(confint(lm(log2.ra$`Sporulation KOs` ~
                                                       Case_status + collection_method + seqs_scaled,
                                                       data=data.frame(sample_data(gene.ps))))[2,2]),
                                     row.names='Sporulation KOs'),
                         lm.path$result.summary[sapply(strsplit(lm.path$result.summary$Feature, ": "),
                                                       function(x){x[1]}) %in% targets &
                                                lm.path$result.summary$Variable == 'Case_status',
                                                c('FC','FC_lower','FC_upper')])
rownames(ssp.ra.fc.data) <- sapply(strsplit(rownames(ssp.ra.fc.data), ": "), function(x){x[1]})
ssp.ra.fc.data <- ssp.ra.fc.data[order(ssp.ra.fc.data$FC, decreasing=TRUE),]
ssp.ra.data <- ssp.ra.data[,rownames(ssp.ra.fc.data), FALSE]
ssp.ra.fc.data <- data.frame(sub_group='serotonin synthesis',
                               plot='Absolute fold change with 95%CI',
                               line='MaAsLin2',
                               variable=rownames(ssp.ra.fc.data), ssp.ra.fc.data)
ssp.ra.data <- data.frame(sub_group='serotonin synthesis', plot='log2(Relative abundances)',
                            Case_status=sample_data(gene.ps)$Case_status, ssp.ra.data)

ssp.ba.data <- cbind(ba.gene[,colnames(ba.gene) %in% targets, FALSE],
                      ba.path[,colnames(ba.path) %in% targets, FALSE])
ssp.ba.fc.data <- rbind(
          ancom.gene$result.summary[sapply(strsplit(ancom.gene$result.summary$Feature, ": "),
                                           function(x){x[1]}) %in% targets &
                                    ancom.gene$result.summary$Variable == 'Case_status',
                                    c('FC','FC_lower','FC_upper')],
          ancom.path$result.summary[sapply(strsplit(ancom.path$result.summary$Feature, ": "),
                                           function(x){x[1]}) %in% targets &
                                    ancom.path$result.summary$Variable == 'Case_status',
                                    c('FC','FC_lower','FC_upper')])
rownames(ssp.ba.fc.data) <- sapply(strsplit(rownames(ssp.ba.fc.data), ": "), function(x){x[1]})
#ssp.ba.fc.data <- ssp.ba.fc.data[rownames(ssp.ra.fc.data),, FALSE]
#ssp.ba.data <- ssp.ba.data[,rownames(ssp.ra.fc.data), FALSE]
ssp.ba.fc.data <- data.frame(sub_group='serotonin synthesis',
                               plot='Absolute fold change with 95%CI',
                               line='ANCOM-BC',
                               variable=rownames(ssp.ba.fc.data), ssp.ba.fc.data)
ssp.ba.data <- data.frame(sub_group='serotonin synthesis', plot='log(Bias-corrected abundances)',
                            Case_status=sample_data(gene.ps)$Case_status, ssp.ba.data)
```

```
# dopamine inhibition
targets <- c('K18933')

dip.ra.data <- cbind(ra.gene[,colnames(ra.gene) %in% targets, FALSE],
                     ra.path[,colnames(ra.path) %in% targets, FALSE])
dip.ra.fc.data <- rbind(
          lm.gene$result.summary[sapply(strsplit(lm.gene$result.summary$Feature, ": "),
                                        function(x){x[1]}) %in% targets &
                                 lm.gene$result.summary$Variable == 'Case_status',
                                 c('FC','FC_lower','FC_upper')],
          lm.path$result.summary[sapply(strsplit(lm.path$result.summary$Feature, ": "),
                                        function(x){x[1]}) %in% targets &
                                 lm.path$result.summary$Variable == 'Case_status',
                                  c('FC','FC_lower','FC_upper')])
rownames(dip.ra.fc.data) <- sapply(strsplit(rownames(dip.ra.fc.data), ": "), function(x){x[1]})
dip.ra.fc.data <- dip.ra.fc.data[order(dip.ra.fc.data$FC, decreasing=FALSE),]
dip.ra.data <- dip.ra.data[,rownames(dip.ra.fc.data), FALSE]
dip.ra.fc.data <- data.frame(sub_group='dopamine inhibition',
                             plot='Absolute fold change with 95%CI',
                             line='MaAsLin2',
                             variable=rownames(dip.ra.fc.data), dip.ra.fc.data)
dip.ra.data <- data.frame(sub_group='dopamine inhibition', plot='log2(Relative abundances)',
                          Case_status=sample_data(gene.ps)$Case_status, dip.ra.data)

dip.ba.data <- cbind(ba.gene[,colnames(ba.gene) %in% targets, FALSE],
                     ba.path[,colnames(ba.path) %in% targets, FALSE])
dip.ba.fc.data <- rbind(
          ancom.gene$result.summary[sapply(strsplit(ancom.gene$result.summary$Feature, ": "),
                                           function(x){x[1]}) %in% targets &
                                    ancom.gene$result.summary$Variable == 'Case_status',
                                    c('FC','FC_lower','FC_upper')],
          ancom.path$result.summary[sapply(strsplit(ancom.path$result.summary$Feature, ": "),
                                           function(x){x[1]}) %in% targets &
                                    ancom.path$result.summary$Variable == 'Case_status',
                                    c('FC','FC_lower','FC_upper')])
rownames(dip.ba.fc.data) <- sapply(strsplit(rownames(dip.ba.fc.data), ": "), function(x){x[1]})
dip.ba.fc.data <- dip.ba.fc.data[rownames(dip.ra.fc.data),, FALSE]
dip.ba.data <- dip.ba.data[,rownames(dip.ra.fc.data), FALSE]
dip.ba.fc.data <- data.frame(sub_group='dopamine inhibition',
                             plot='Absolute fold change with 95%CI',
                             line='ANCOM-BC',
                             variable=rownames(dip.ba.fc.data), dip.ba.fc.data)
dip.ba.data <- data.frame(sub_group='dopamine inhibition', plot='log(Bias-corrected abundances)',
                          Case_status=sample_data(gene.ps)$Case_status, dip.ba.data)

# glutamate/GABA degradation
targets <- c('PWY-5088','ARGDEG-PWY')

ggd.ra.data <- cbind(ra.gene[,colnames(ra.gene) %in% targets, FALSE],
                     ra.path[,colnames(ra.path) %in% targets, FALSE])
ggd.ra.fc.data <- rbind(
          lm.gene$result.summary[sapply(strsplit(lm.gene$result.summary$Feature, ": "),
                                        function(x){x[1]}) %in% targets &
```

```
                                  lm.gene$result.summary$Variable == 'Case_status',
                                  c('FC','FC_lower','FC_upper')],
            lm.path$result.summary[sapply(strsplit(lm.path$result.summary$Feature, ": "),
                                         function(x){x[1]}) %in% targets &
                                  lm.path$result.summary$Variable == 'Case_status',
                                   c('FC','FC_lower','FC_upper')])
rownames(ggd.ra.fc.data) <- sapply(strsplit(rownames(ggd.ra.fc.data), ": "), function(x){x[1]})
ggd.ra.fc.data <- ggd.ra.fc.data[order(ggd.ra.fc.data$FC, decreasing=FALSE),]
ggd.ra.data <- ggd.ra.data[,rownames(ggd.ra.fc.data), FALSE]
ggd.ra.fc.data <- data.frame(sub_group='glutamate/GABA degradation',
                              plot='Absolute fold change with 95%CI',
                              line='MaAsLin2',
                              variable=rownames(ggd.ra.fc.data), ggd.ra.fc.data)
ggd.ra.data <- data.frame(sub_group='glutamate/GABA degradation',
                           plot='log2(Relative abundances)',
                           Case_status=sample_data(gene.ps)$Case_status, ggd.ra.data)

ggd.ba.data <- cbind(ba.gene[,colnames(ba.gene) %in% targets, FALSE],
                     ba.path[,colnames(ba.path) %in% targets, FALSE])
ggd.ba.fc.data <- rbind(
            ancom.gene$result.summary[sapply(strsplit(ancom.gene$result.summary$Feature, ": "),
                                            function(x){x[1]}) %in% targets &
                                     ancom.gene$result.summary$Variable == 'Case_status',
                                     c('FC','FC_lower','FC_upper')],
            ancom.path$result.summary[sapply(strsplit(ancom.path$result.summary$Feature, ": "),
                                            function(x){x[1]}) %in% targets &
                                     ancom.path$result.summary$Variable == 'Case_status',
                                     c('FC','FC_lower','FC_upper')])
rownames(ggd.ba.fc.data) <- sapply(strsplit(rownames(ggd.ba.fc.data), ": "), function(x){x[1]})
ggd.ba.fc.data <- ggd.ba.fc.data[rownames(ggd.ra.fc.data),, FALSE]
ggd.ba.data <- ggd.ba.data[,rownames(ggd.ra.fc.data), FALSE]
ggd.ba.fc.data <- data.frame(sub_group='glutamate/GABA degradation',
                              plot='Absolute fold change with 95%CI',
                              line='ANCOM-BC',
                              variable=rownames(ggd.ba.fc.data), ggd.ba.fc.data)
ggd.ba.data <- data.frame(sub_group='glutamate/GABA degradation',
                           plot='log(Bias-corrected abundances)',
                           Case_status=sample_data(gene.ps)$Case_status, ggd.ba.data)

# combine
fc.plot.data <- rbind(fc.plot.data,
                      data.frame(group='Dysregulated neuroactive signaling',
                                 rbind(dsp.ra.fc.data, gsp.ra.fc.data, ssp.ra.fc.data,
                                       dip.ra.fc.data, ggd.ra.fc.data,
                                       dsp.ba.fc.data, gsp.ba.fc.data, ssp.ba.fc.data,
                                       dip.ba.fc.data, ggd.ba.fc.data)))
ab.plot.data <- merge(ab.plot.data,
                      merge(
                       data.frame(group='Dysregulated neuroactive signaling',
                                  merge(dsp.ra.data,
                                    merge(gsp.ra.data,
                                      merge(ssp.ra.data,
                                        merge(dip.ra.data, ggd.ra.data,
                                              all=TRUE, sort=FALSE),
```

```r
                                         all=TRUE, sort=FALSE),
                                       all=TRUE, sort=FALSE),
                                     all=TRUE, sort=FALSE)),
                       data.frame(group='Dysregulated neuroactive signaling',
                                 merge(dsp.ba.data,
                                   merge(gsp.ba.data,
                                     merge(ssp.ba.data,
                                       merge(dip.ba.data, ggd.ba.data,
                                           all=TRUE, sort=FALSE),
                                         all=TRUE, sort=FALSE),
                                       all=TRUE, sort=FALSE),
                                     all=TRUE, sort=FALSE)),
                            all=TRUE, sort=FALSE),
                       all=TRUE, sort=FALSE)
colnames(ab.plot.data) <- gsub('\\.','-',colnames(ab.plot.data))
colnames(ab.plot.data) <- gsub('Sporulation-KOs','Sporulation KOs',colnames(ab.plot.data))

## reduced neuroprotective molecules
# nicotinamide degradation
targets <- c('K08281')

ndp.ra.data <- cbind(ra.gene[,colnames(ra.gene) %in% targets, FALSE],
                     ra.path[,colnames(ra.path) %in% targets, FALSE])
ndp.ra.fc.data <- rbind(
          lm.gene$result.summary[sapply(strsplit(lm.gene$result.summary$Feature, ": "),
                                        function(x){x[1]}) %in% targets &
                                 lm.gene$result.summary$Variable == 'Case_status',
                                 c('FC','FC_lower','FC_upper')],
          lm.path$result.summary[sapply(strsplit(lm.path$result.summary$Feature, ": "),
                                        function(x){x[1]}) %in% targets &
                                 lm.path$result.summary$Variable == 'Case_status',
                                  c('FC','FC_lower','FC_upper')])
rownames(ndp.ra.fc.data) <- sapply(strsplit(rownames(ndp.ra.fc.data), ": "), function(x){x[1]})
ndp.ra.fc.data <- ndp.ra.fc.data[order(ndp.ra.fc.data$FC, decreasing=FALSE),]
ndp.ra.data <- ndp.ra.data[,rownames(ndp.ra.fc.data), FALSE]
ndp.ra.fc.data <- data.frame(sub_group='nicotinamide degradation',
                             plot='Absolute fold change with 95%CI',
                             line='MaAsLin2',
                             variable=rownames(ndp.ra.fc.data), ndp.ra.fc.data)
ndp.ra.data <- data.frame(sub_group='nicotinamide degradation',
                          plot='log2(Relative abundances)',
                          Case_status=sample_data(gene.ps)$Case_status, ndp.ra.data)

ndp.ba.data <- cbind(ba.gene[,colnames(ba.gene) %in% targets, FALSE],
                     ba.path[,colnames(ba.path) %in% targets, FALSE])
ndp.ba.fc.data <- rbind(
          ancom.gene$result.summary[sapply(strsplit(ancom.gene$result.summary$Feature, ": "),
                                           function(x){x[1]}) %in% targets &
                                    ancom.gene$result.summary$Variable == 'Case_status',
                                    c('FC','FC_lower','FC_upper')],
          ancom.path$result.summary[sapply(strsplit(ancom.path$result.summary$Feature, ": "),
                                           function(x){x[1]}) %in% targets &
                                    ancom.path$result.summary$Variable == 'Case_status',
                                    c('FC','FC_lower','FC_upper')])
```

```r
rownames(ndp.ba.fc.data) <- sapply(strsplit(rownames(ndp.ba.fc.data), ": "), function(x){x[1]})
ndp.ba.fc.data <- ndp.ba.fc.data[rownames(ndp.ra.fc.data),, FALSE]
ndp.ba.data <- ndp.ba.data[,rownames(ndp.ra.fc.data), FALSE]
ndp.ba.fc.data <- data.frame(sub_group='nicotinamide degradation',
                             plot='Absolute fold change with 95%CI',
                             line='ANCOM-BC',
                             variable=rownames(ndp.ba.fc.data), ndp.ba.fc.data)
ndp.ba.data <- data.frame(sub_group='nicotinamide degradation',
                          plot='log(Bias-corrected abundances)',
                          Case_status=sample_data(gene.ps)$Case_status, ndp.ba.data)

# trehalose degradation
targets <- c('K00697','K01087','PWY-2723')

tdp.ra.data <- cbind(ra.gene[,colnames(ra.gene) %in% targets, FALSE],
                     ra.path[,colnames(ra.path) %in% targets, FALSE])
tdp.ra.fc.data <- rbind(
          lm.gene$result.summary[sapply(strsplit(lm.gene$result.summary$Feature, ": "),
                                        function(x){x[1]}) %in% targets &
                                 lm.gene$result.summary$Variable == 'Case_status',
                                 c('FC','FC_lower','FC_upper')],
          lm.path$result.summary[sapply(strsplit(lm.path$result.summary$Feature, ": "),
                                        function(x){x[1]}) %in% targets &
                                 lm.path$result.summary$Variable == 'Case_status',
                                  c('FC','FC_lower','FC_upper')])
rownames(tdp.ra.fc.data) <- sapply(strsplit(rownames(tdp.ra.fc.data), ": "), function(x){x[1]})
tdp.ra.fc.data <- tdp.ra.fc.data[order(tdp.ra.fc.data$FC, decreasing=FALSE),]
tdp.ra.data <- tdp.ra.data[,rownames(tdp.ra.fc.data), FALSE]
tdp.ra.fc.data <- data.frame(sub_group='trehalose degradation and metabolism',
                             plot='Absolute fold change with 95%CI', line='MaAsLin2',
                             variable=rownames(tdp.ra.fc.data), tdp.ra.fc.data)
tdp.ra.data <- data.frame(sub_group='trehalose degradation and metabolism',
                          plot='log2(Relative abundances)',
                          Case_status=sample_data(gene.ps)$Case_status, tdp.ra.data)

tdp.ba.data <- cbind(ba.gene[,colnames(ba.gene) %in% targets, FALSE],
                     ba.path[,colnames(ba.path) %in% targets, FALSE])
tdp.ba.fc.data <- rbind(
          ancom.gene$result.summary[sapply(strsplit(ancom.gene$result.summary$Feature, ": "),
                                        function(x){x[1]}) %in% targets &
                                 ancom.gene$result.summary$Variable == 'Case_status',
                                 c('FC','FC_lower','FC_upper')],
          ancom.path$result.summary[sapply(strsplit(ancom.path$result.summary$Feature, ": "),
                                        function(x){x[1]}) %in% targets &
                                 ancom.path$result.summary$Variable == 'Case_status',
                                 c('FC','FC_lower','FC_upper')])
rownames(tdp.ba.fc.data) <- sapply(strsplit(rownames(tdp.ba.fc.data), ": "), function(x){x[1]})
tdp.ba.fc.data <- tdp.ba.fc.data[rownames(tdp.ra.fc.data),, FALSE]
tdp.ba.data <- tdp.ba.data[,rownames(tdp.ra.fc.data), FALSE]
tdp.ba.fc.data <- data.frame(sub_group='trehalose degradation and metabolism',
                             plot='Absolute fold change with 95%CI', line='ANCOM-BC',
                             variable=rownames(tdp.ba.fc.data), tdp.ba.fc.data)
tdp.ba.data <- data.frame(sub_group='trehalose degradation and metabolism',
                          plot='log(Bias-corrected abundances)',
```

```
                              Case_status=sample_data(gene.ps)$Case_status, tdp.ba.data)

# combine
fc.plot.data <- rbind(fc.plot.data,
                      data.frame(group='Reduced neuroprotective molecules',
                                 rbind(ndp.ra.fc.data, tdp.ra.fc.data,
                                       ndp.ba.fc.data, tdp.ba.fc.data)))
ab.plot.data <- merge(ab.plot.data,
                      merge(data.frame(group='Reduced neuroprotective molecules',
                                       merge(ndp.ra.data, tdp.ra.data, all=TRUE, sort=FALSE)),
                            data.frame(group='Reduced neuroprotective molecules',
                                       merge(ndp.ba.data, tdp.ba.data, all=TRUE, sort=FALSE)),
                            all=TRUE, sort=FALSE),
                      all=TRUE, sort=FALSE)
colnames(ab.plot.data) <- gsub('\\.','-',colnames(ab.plot.data))

## elevated bacterial amyloid curli
# curli production
targets <- c('K04334','K04336','K06214')

cpp.ra.data <- cbind(ra.gene[,colnames(ra.gene) %in% targets, FALSE],
                     ra.path[,colnames(ra.path) %in% targets, FALSE])
cpp.ra.fc.data <- rbind(
          lm.gene$result.summary[sapply(strsplit(lm.gene$result.summary$Feature, ": "),
                                        function(x){x[1]}) %in% targets &
                                 lm.gene$result.summary$Variable == 'Case_status',
                                 c('FC','FC_lower','FC_upper')],
          lm.path$result.summary[sapply(strsplit(lm.path$result.summary$Feature, ": "),
                                        function(x){x[1]}) %in% targets &
                                 lm.path$result.summary$Variable == 'Case_status',
                                  c('FC','FC_lower','FC_upper')])
rownames(cpp.ra.fc.data) <- sapply(strsplit(rownames(cpp.ra.fc.data), ": "), function(x){x[1]})
cpp.ra.fc.data <- cpp.ra.fc.data[order(cpp.ra.fc.data$FC, decreasing=FALSE),]
cpp.ra.data <- cpp.ra.data[,rownames(cpp.ra.fc.data), FALSE]
cpp.ra.fc.data <- data.frame(sub_group='curli production',
                             plot='Absolute fold change with 95%CI',
                             line='MaAsLin2',
                             variable=rownames(cpp.ra.fc.data), cpp.ra.fc.data)
cpp.ra.data <- data.frame(sub_group='curli production', plot='log2(Relative abundances)',
                          Case_status=sample_data(gene.ps)$Case_status, cpp.ra.data)

cpp.ba.data <- cbind(ba.gene[,colnames(ba.gene) %in% targets, FALSE],
                     ba.path[,colnames(ba.path) %in% targets, FALSE])
cpp.ba.fc.data <- rbind(
          ancom.gene$result.summary[sapply(strsplit(ancom.gene$result.summary$Feature, ": "),
                                           function(x){x[1]}) %in% targets &
                                    ancom.gene$result.summary$Variable == 'Case_status',
                                    c('FC','FC_lower','FC_upper')],
          ancom.path$result.summary[sapply(strsplit(ancom.path$result.summary$Feature, ": "),
                                           function(x){x[1]}) %in% targets &
                                    ancom.path$result.summary$Variable == 'Case_status',
                                    c('FC','FC_lower','FC_upper')])
rownames(cpp.ba.fc.data) <- sapply(strsplit(rownames(cpp.ba.fc.data), ": "), function(x){x[1]})
cpp.ba.fc.data <- cpp.ba.fc.data[rownames(cpp.ra.fc.data),, FALSE]
```

```
cpp.ba.data <- cpp.ba.data[,rownames(cpp.ra.fc.data), FALSE]
cpp.ba.fc.data <- data.frame(sub_group='curli production',
                             plot='Absolute fold change with 95%CI',
                             line='ANCOM-BC',
                             variable=rownames(cpp.ba.fc.data), cpp.ba.fc.data)
cpp.ba.data <- data.frame(sub_group='curli production', plot='log(Bias-corrected abundances)',
                          Case_status=sample_data(gene.ps)$Case_status, cpp.ba.data)

# curli regulation
targets <- c('K21963')

crp.ra.data <- cbind(ra.gene[,colnames(ra.gene) %in% targets, FALSE],
                     ra.path[,colnames(ra.path) %in% targets, FALSE])
crp.ra.fc.data <- rbind(
          lm.gene$result.summary[sapply(strsplit(lm.gene$result.summary$Feature, ": "),
                                        function(x){x[1]}) %in% targets &
                                 lm.gene$result.summary$Variable == 'Case_status',
                                 c('FC','FC_lower','FC_upper')],
          lm.path$result.summary[sapply(strsplit(lm.path$result.summary$Feature, ": "),
                                        function(x){x[1]}) %in% targets &
                                 lm.path$result.summary$Variable == 'Case_status',
                                  c('FC','FC_lower','FC_upper')])
rownames(crp.ra.fc.data) <- sapply(strsplit(rownames(crp.ra.fc.data), ": "), function(x){x[1]})
crp.ra.fc.data <- crp.ra.fc.data[order(crp.ra.fc.data$FC, decreasing=FALSE),]
crp.ra.data <- crp.ra.data[,rownames(crp.ra.fc.data), FALSE]
crp.ra.fc.data <- data.frame(sub_group='curli regulation',
                             plot='Absolute fold change with 95%CI',
                             line='MaAsLin2',
                             variable=rownames(crp.ra.fc.data), crp.ra.fc.data)
crp.ra.data <- data.frame(sub_group='curli regulation', plot='log2(Relative abundances)',
                          Case_status=sample_data(gene.ps)$Case_status, crp.ra.data)

crp.ba.data <- cbind(ba.gene[,colnames(ba.gene) %in% targets, FALSE],
                     ba.path[,colnames(ba.path) %in% targets, FALSE])
crp.ba.fc.data <- rbind(
          ancom.gene$result.summary[sapply(strsplit(ancom.gene$result.summary$Feature, ": "),
                                           function(x){x[1]}) %in% targets &
                                    ancom.gene$result.summary$Variable == 'Case_status',
                                    c('FC','FC_lower','FC_upper')],
          ancom.path$result.summary[sapply(strsplit(ancom.path$result.summary$Feature, ": "),
                                           function(x){x[1]}) %in% targets &
                                    ancom.path$result.summary$Variable == 'Case_status',
                                    c('FC','FC_lower','FC_upper')])
rownames(crp.ba.fc.data) <- sapply(strsplit(rownames(crp.ba.fc.data), ": "), function(x){x[1]})
crp.ba.fc.data <- crp.ba.fc.data[rownames(crp.ra.fc.data),, FALSE]
crp.ba.data <- crp.ba.data[,rownames(crp.ra.fc.data), FALSE]
crp.ba.fc.data <- data.frame(sub_group='curli regulation',
                             plot='Absolute fold change with 95%CI',
                             line='ANCOM-BC',
                             variable=rownames(crp.ba.fc.data), crp.ba.fc.data)
crp.ba.data <- data.frame(sub_group='curli regulation', plot='log(Bias-corrected abundances)',
                          Case_status=sample_data(gene.ps)$Case_status, crp.ba.data)

# combine
```

```r
fc.plot.data <- rbind(fc.plot.data,
  data.frame(group='Elevated curli, a bacterial amyloid, triggers alpha-synuclein pathology',
             rbind(cpp.ra.fc.data, crp.ra.fc.data, cpp.ba.fc.data, crp.ba.fc.data)))
ab.plot.data <- merge(ab.plot.data,
  merge(data.frame(group='Elevated curli, a bacterial amyloid, triggers alpha-synuclein pathology',
                   merge(cpp.ra.data, crp.ra.data, all=TRUE, sort=FALSE)),
        data.frame(group='Elevated curli, a bacterial amyloid, triggers alpha-synuclein pathology',
                   merge(cpp.ba.data, crp.ba.data, all=TRUE, sort=FALSE)),
        all=TRUE, sort=FALSE),
  all=TRUE, sort=FALSE)
colnames(ab.plot.data) <- gsub('\\.','-',colnames(ab.plot.data))

## elevated toxic metabolite
# TMA from choline
targets <- c('K20038')

tch.ra.data <- cbind(ra.gene[,colnames(ra.gene) %in% targets, FALSE],
                     ra.path[,colnames(ra.path) %in% targets, FALSE])
tch.ra.fc.data <- rbind(
          lm.gene$result.summary[sapply(strsplit(lm.gene$result.summary$Feature, ": "),
                                        function(x){x[1]}) %in% targets &
                                 lm.gene$result.summary$Variable == 'Case_status',
                                 c('FC','FC_lower','FC_upper')],
          lm.path$result.summary[sapply(strsplit(lm.path$result.summary$Feature, ": "),
                                        function(x){x[1]}) %in% targets &
                                 lm.path$result.summary$Variable == 'Case_status',
                                  c('FC','FC_lower','FC_upper')])
rownames(tch.ra.fc.data) <- sapply(strsplit(rownames(tch.ra.fc.data), ": "), function(x){x[1]})
tch.ra.fc.data <- tch.ra.fc.data[order(tch.ra.fc.data$FC, decreasing=FALSE),]
tch.ra.data <- tch.ra.data[,rownames(tch.ra.fc.data), FALSE]
tch.ra.fc.data <- data.frame(sub_group='TMA from choline',
                             plot='Absolute fold change with 95%CI',
                             line='MaAsLin2',
                             variable=rownames(tch.ra.fc.data), tch.ra.fc.data)
tch.ra.data <- data.frame(sub_group='TMA from choline', plot='log2(Relative abundances)',
                          Case_status=sample_data(gene.ps)$Case_status, tch.ra.data)

tch.ba.data <- cbind(ba.gene[,colnames(ba.gene) %in% targets, FALSE],
                     ba.path[,colnames(ba.path) %in% targets, FALSE])
tch.ba.fc.data <- rbind(
          ancom.gene$result.summary[sapply(strsplit(ancom.gene$result.summary$Feature, ": "),
                                           function(x){x[1]}) %in% targets &
                                    ancom.gene$result.summary$Variable == 'Case_status',
                                    c('FC','FC_lower','FC_upper')],
          ancom.path$result.summary[sapply(strsplit(ancom.path$result.summary$Feature, ": "),
                                           function(x){x[1]}) %in% targets &
                                    ancom.path$result.summary$Variable == 'Case_status',
                                    c('FC','FC_lower','FC_upper')])
rownames(tch.ba.fc.data) <- sapply(strsplit(rownames(tch.ba.fc.data), ": "), function(x){x[1]})
tch.ba.fc.data <- tch.ba.fc.data[rownames(tch.ra.fc.data),, FALSE]
tch.ba.data <- tch.ba.data[,rownames(tch.ra.fc.data), FALSE]
tch.ba.fc.data <- data.frame(sub_group='TMA from choline',
                             plot='Absolute fold change with 95%CI',
                             line='ANCOM-BC',
```

```
                              variable=rownames(tch.ba.fc.data), tch.ba.fc.data)
tch.ba.data <- data.frame(sub_group='TMA from choline', plot='log(Bias-corrected abundances)',
                           Case_status=sample_data(gene.ps)$Case_status, tch.ba.data)

# TMA from carnitine
targets <- c('K05245')

tca.ra.data <- cbind(ra.gene[,colnames(ra.gene) %in% targets, FALSE],
                     ra.path[,colnames(ra.path) %in% targets, FALSE])
tca.ra.fc.data <- rbind(
          lm.gene$result.summary[sapply(strsplit(lm.gene$result.summary$Feature, ": "),
                                        function(x){x[1]}) %in% targets &
                                 lm.gene$result.summary$Variable == 'Case_status',
                                 c('FC','FC_lower','FC_upper')],
          lm.path$result.summary[sapply(strsplit(lm.path$result.summary$Feature, ": "),
                                        function(x){x[1]}) %in% targets &
                                 lm.path$result.summary$Variable == 'Case_status',
                                  c('FC','FC_lower','FC_upper')])
rownames(tca.ra.fc.data) <- sapply(strsplit(rownames(tca.ra.fc.data), ": "), function(x){x[1]})
tca.ra.fc.data <- tca.ra.fc.data[order(tca.ra.fc.data$FC, decreasing=FALSE),]
tca.ra.data <- tca.ra.data[,rownames(tca.ra.fc.data), FALSE]
tca.ra.fc.data <- data.frame(sub_group='TMA from carnitine',
                              plot='Absolute fold change with 95%CI',
                              line='MaAsLin2',
                              variable=rownames(tca.ra.fc.data), tca.ra.fc.data)
tca.ra.data <- data.frame(sub_group='TMA from carnitine', plot='log2(Relative abundances)',
                           Case_status=sample_data(gene.ps)$Case_status, tca.ra.data)

tca.ba.data <- cbind(ba.gene[,colnames(ba.gene) %in% targets, FALSE],
                     ba.path[,colnames(ba.path) %in% targets, FALSE])
tca.ba.fc.data <- rbind(
          ancom.gene$result.summary[sapply(strsplit(ancom.gene$result.summary$Feature, ": "),
                                           function(x){x[1]}) %in% targets &
                                    ancom.gene$result.summary$Variable == 'Case_status',
                                    c('FC','FC_lower','FC_upper')],
          ancom.path$result.summary[sapply(strsplit(ancom.path$result.summary$Feature, ": "),
                                           function(x){x[1]}) %in% targets &
                                    ancom.path$result.summary$Variable == 'Case_status',
                                    c('FC','FC_lower','FC_upper')])
rownames(tca.ba.fc.data) <- sapply(strsplit(rownames(tca.ba.fc.data), ": "), function(x){x[1]})
tca.ba.fc.data <- tca.ba.fc.data[rownames(tca.ra.fc.data),, FALSE]
tca.ba.data <- tca.ba.data[,rownames(tca.ra.fc.data), FALSE]
tca.ba.fc.data <- data.frame(sub_group='TMA from carnitine',
                              plot='Absolute fold change with 95%CI',
                              line='ANCOM-BC',
                              variable=rownames(tca.ba.fc.data), tca.ba.fc.data)
tca.ba.data <- data.frame(sub_group='TMA from carnitine', plot='log(Bias-corrected abundances)',
                           Case_status=sample_data(gene.ps)$Case_status, tca.ba.data)

# combine
fc.plot.data <- rbind(fc.plot.data,
                      data.frame(group='Elevated toxic metabolite',
                                 rbind(tch.ra.fc.data, tca.ra.fc.data,
                                       tch.ba.fc.data, tca.ba.fc.data)))
```

```
ab.plot.data <- merge(ab.plot.data,
                      merge(data.frame(group='Elevated toxic metabolite',
                                       merge(tch.ra.data, tca.ra.data, all=TRUE, sort=FALSE)),
                            data.frame(group='Elevated toxic metabolite',
                                       merge(tch.ba.data, tca.ba.data, all=TRUE, sort=FALSE)),
                            all=TRUE, sort=FALSE),
                      all=TRUE, sort=FALSE)
colnames(ab.plot.data) <- gsub('\\.','-',colnames(ab.plot.data))

# prep fold change data for plotting
fc.plot.data$group <- factor(fc.plot.data$group, levels=unique(fc.plot.data$group))
fc.plot.data$sub_group <- factor(fc.plot.data$sub_group, levels=unique(fc.plot.data$sub_group))
fc.plot.data$line <- factor(fc.plot.data$line, levels=rev(unique(fc.plot.data$line)))
fc.plot.data$variable <- factor(fc.plot.data$variable, levels=unique(fc.plot.data$variable))
fc.plot.data$color[fc.plot.data$FC < 1] <- 'elevated'
fc.plot.data$color[fc.plot.data$FC > 1] <- 'depleted'
fc.plot.data$FC_mod[fc.plot.data$FC > 1] <- fc.plot.data$FC[fc.plot.data$FC > 1]-1
fc.plot.data$FC_mod[fc.plot.data$FC < 1] <- -((1/fc.plot.data$FC[fc.plot.data$FC < 1])-1)
fc.plot.data$FC_lower_mod[fc.plot.data$FC_lower > 1] <-
                fc.plot.data$FC_lower[fc.plot.data$FC_lower > 1]-1
fc.plot.data$FC_lower_mod[fc.plot.data$FC_lower < 1] <-
                -((1/fc.plot.data$FC_lower[fc.plot.data$FC_lower < 1])-1)
fc.plot.data$FC_upper_mod[fc.plot.data$FC_upper > 1] <-
                fc.plot.data$FC_upper[fc.plot.data$FC_upper > 1]-1
fc.plot.data$FC_upper_mod[fc.plot.data$FC_upper < 1] <-
                -((1/fc.plot.data$FC_upper[fc.plot.data$FC_upper < 1])-1)

# prep abundance data for plotting
ab.plot.data$group <- factor(ab.plot.data$group, levels=unique(ab.plot.data$group))
ab.plot.data$sub_group <- factor(ab.plot.data$sub_group, levels=unique(ab.plot.data$sub_group))
ab.plot.data$Case_status <- dplyr::recode(ab.plot.data$Case_status, '1'='PD', '0'='NHC')
ab.plot.data$Case_status <- factor(ab.plot.data$Case_status,
                                   levels=rev(unique(ab.plot.data$Case_status)))
ab.plot.data$plot <- factor(ab.plot.data$plot, levels=unique(ab.plot.data$plot))
ab.plot.data.melt <- reshape2::melt(ab.plot.data)
ab.plot.data.melt <- ab.plot.data.melt[!is.na(ab.plot.data.melt$value),]
ab.plot.data.melt$value[ab.plot.data.melt$plot == 'log2(Relative abundances)'] <-
      log2.trans(ab.plot.data.melt$value[ab.plot.data.melt$plot == 'log2(Relative abundances)'])
ab.plot.data.melt$value[ab.plot.data.melt$plot == 'log(Bias-corrected abundances)'] <-
      ab.plot.data.melt$value[ab.plot.data.melt$plot == 'log(Bias-corrected abundances)']+10

# merge data
plot.data <- merge(ab.plot.data.melt, fc.plot.data, all=TRUE, sort=FALSE)
plot.data$plot <- factor(plot.data$plot, levels=unique(plot.data$plot))

# add gene annotations to KOs
plot.data$variable <- factor(gsub('K02535', 'K02535 (lpxC)', plot.data$variable),
                             levels=gsub('K02535', 'K02535 (lpxC)',
                                         unique(plot.data$variable)))
plot.data$variable <- factor(gsub('K04744', 'K04744 (lptD, imp, ostA)', plot.data$variable),
                             levels=gsub('K04744', 'K04744 (lptD, imp, ostA)',
                                         unique(plot.data$variable)))
plot.data$variable <- factor(gsub('K09949', 'K09949 (lpxI)', plot.data$variable),
                             levels=gsub('K09949', 'K09949 (lpxI)',
```

```
                                        unique(plot.data$variable)))
plot.data$variable <- factor(gsub('K19005', 'K19005 (ltaS)', plot.data$variable),
                             levels=gsub('K19005', 'K19005 (ltaS)',
                                         unique(plot.data$variable)))
plot.data$variable <- factor(gsub('K03739', 'K03739 (dltB)', plot.data$variable),
                             levels=gsub('K03739', 'K03739 (dltB)',
                                         unique(plot.data$variable)))
plot.data$variable <- factor(gsub('K06078', 'K06078 (lpp)', plot.data$variable),
                             levels=gsub('K06078', 'K06078 (lpp)',
                                         unique(plot.data$variable)))
plot.data$variable <- factor(gsub('K17236', 'K17236 (araQ)', plot.data$variable),
                             levels=gsub('K17236', 'K17236 (araQ)',
                                         unique(plot.data$variable)))
plot.data$variable <- factor(gsub('K16213', 'K16213 (cbe, mbe)', plot.data$variable),
                             levels=gsub('K16213', 'K16213 (cbe, mbe)',
                                         unique(plot.data$variable)))
plot.data$variable <- factor(gsub('K17234', 'K17234 (araN)', plot.data$variable),
                             levels=gsub('K17234', 'K17234 (araN)',
                                         unique(plot.data$variable)))
plot.data$variable <- factor(gsub('K00702', 'K00702 (E2.4.1.20)', plot.data$variable),
                             levels=gsub('K00702', 'K00702 (E2.4.1.20)',
                                         unique(plot.data$variable)))
plot.data$variable <- factor(gsub('K00266', 'K00266 (gltD)', plot.data$variable),
                             levels=gsub('K00266', 'K00266 (gltD)',
                                         unique(plot.data$variable)))
plot.data$variable <- factor(gsub('K18933', 'K18933 (mfnA, adc)', plot.data$variable),
                             levels=gsub('K18933', 'K18933 (mfnA, adc)',
                                         unique(plot.data$variable)))
plot.data$variable <- factor(gsub('K08281', 'K08281 (pncA)', plot.data$variable),
                             levels=gsub('K08281', 'K08281 (pncA)',
                                         unique(plot.data$variable)))
plot.data$variable <- factor(gsub('K01087', 'K01087 (otsB)', plot.data$variable),
                             levels=gsub('K01087', 'K01087 (otsB)',
                                         unique(plot.data$variable)))
plot.data$variable <- factor(gsub('K00697', 'K00697 (otsA)', plot.data$variable),
                             levels=gsub('K00697', 'K00697 (otsA)',
                                         unique(plot.data$variable)))
plot.data$variable <- factor(gsub('K06214', 'K06214 (csgG)', plot.data$variable),
                             levels=gsub('K06214', 'K06214 (csgG)',
                                         unique(plot.data$variable)))
plot.data$variable <- factor(gsub('K04336', 'K04336 (csgC)', plot.data$variable),
                             levels=gsub('K04336', 'K04336 (csgC)',
                                         unique(plot.data$variable)))
plot.data$variable <- factor(gsub('K04334', 'K04334 (csgA)', plot.data$variable),
                             levels=gsub('K04334', 'K04334 (csgA)',
                                         unique(plot.data$variable)))
plot.data$variable <- factor(gsub('K21963', 'K21963 (ecpR, matA)', plot.data$variable),
                             levels=gsub('K21963', 'K21963 (ecpR, matA)',
                                         unique(plot.data$variable)))
plot.data$variable <- factor(gsub('K20038', 'K20038 (cutC)', plot.data$variable),
                             levels=gsub('K20038', 'K20038 (cutC)',
                                         unique(plot.data$variable)))
plot.data$variable <- factor(gsub('K05245', 'K05245 (caiT)', plot.data$variable),
                             levels=gsub('K05245', 'K05245 (caiT)',
```

```r
                                          unique(plot.data$variable)))

# create breaks and break labels for plot
breaks <- c(-25,-20,-15,-10,-3,-2,-1,0,1,2,7.5,10,15,20)
break_labels <- c(paste(breaks[1:4],'\n(',
                        gsub('e-0','e-',formatC(2^breaks[1:4],format='e',digits=0)),
                        ')',sep=''),
                  paste(gsub('1','0', abs(breaks[5:10])+1), 'x',sep=''),
                  paste(breaks[11:14]-10,'\n(',round(exp(breaks[11:14]-10),1),')',sep=''))

# create plot
g <- ggplot(data=plot.data[grep('log', plot.data$plot),],
            aes(x=variable, y=value, fill=as.character(Case_status))) +
        geom_boxplot(notch=FALSE, outlier.size=0.5) +
        geom_errorbar(inherit.aes=FALSE,
                      data=plot.data[plot.data$plot=='Absolute fold change with 95%CI',],
                      aes(x=variable, ymin=FC_lower_mod, ymax=FC_upper_mod,
                          color=color, linetype=line),
                      width=0, position=position_dodge(0.75), size=0.75) +
        geom_point(inherit.aes=FALSE,
                   data=plot.data[plot.data$plot=='Absolute fold change with 95%CI',],
                   aes(x=variable, y=FC_mod, color=color, pch=line),
                   position=position_dodge(0.75), size=1.75) +
        geom_hline(data=plot.data[plot.data$plot=='Absolute fold change with 95%CI',],
                   aes(yintercept=0), size=0.5, linetype='dashed', alpha=0.5) +
        facet_nested(group + sub_group ~ plot, scales='free', space='free_y', switch='y',
                     strip=strip_nested(text_y=list(element_text(angle=0))),
                     labeller=labeller(group=label_wrap_gen(width=10),
                                       sub_group=label_wrap_gen(width=10))) +
        scale_x_discrete(position='bottom') +
        scale_y_continuous(position='right', breaks=breaks, labels=break_labels) +
        coord_flip() +
        scale_fill_manual(values=c("#E69F00", "#00BFC4")) +
        scale_color_manual(values=c("blue", "red"), labels=c("elevated","depleted")) +
        scale_linetype_manual(values=c("11", "solid")) +
        scale_shape_manual(values=c(16, 15)) +
        guides(fill=guide_legend(order=1, title="Subject group", title.position="top"),
               color=guide_legend(order=2, title="Fold change direction", title.position="top"),
               linetype=guide_legend(title="Fold change source", title.position="top", reverse=TRUE),
               pch=guide_legend(title="Fold change source", title.position="top", reverse=TRUE)) +
        theme(legend.position="top", legend.key=element_blank(), legend.title=element_text(size=8),
              legend.text=element_text(size=8),
              axis.title.x=element_blank(), axis.title.y=element_blank(),
              axis.text.y=element_text(size=8),
              strip.text=element_text(size=8),
              strip.background=element_rect(fill='gray90', color='gray'),
              strip.placement="outside", panel.spacing.y=unit(0.5, "lines"))
ggsave(
'PDShotgunAnalysis_out/5.Gene_pathway_associations/KO_pathway_distributions_foldchanges.pdf',
g, device='pdf', width=12, height=20)
```

Secondary analyses (replication driven)

The following analyses were conducted for the purposes of replicating prior 16S results.

Replicating signal for SILVA v 132 Prevotella

To test if *Prevotella* species previously classified into the sub-genus "*Prevotella*" in SILVA v 132 (used in previous 16S analysis from *Wallen et al. 2020 npj Parkinsons Dis*) would be signficantly associated with PD as a group in this dataset, collapsed relative abundances of *Prevotella* species in this dataset that were mapped to ASVs in *Prevotella* sub-genus of *Wallen et al. 2020 npj Parkinsons Dis* datasets (*P. buccalis*, *P. timonensis*, *P. bivia*, *P. disiens*, and *P. oralis*) into one group and tested for association with PD using linear regression (via `lm` function) with log2 transformed relative abundances (as done with MaAsLin2).

```
#### REPLICATING SILVA V 132 PREVOTELLA ####

# define target species to be collapsed
target_taxa <- c("Prevotella_buccalis","Prevotella_timonensis",
                 "Prevotella_bivia","Prevotella_disiens","Prevotella_oralis")

# collapse species relative abundances into one group
ra.mod <- data.frame(otu_table(ra.ps.s)/100)
colnames(ra.mod) <- sapply(strsplit(as.character(colnames(ra.mod)), "s__"), function(x){x[2]})
ra.mod <- data.frame(target_cluster=rowSums(ra.mod[,colnames(ra.mod) %in% target_taxa]),
                     ra.mod[,!(colnames(ra.mod) %in% target_taxa)])

# log2 transform
log2.ra <- data.frame(apply(ra.mod, 2, log2.trans))

# perform linear regression
ra.lm <- lm(log2.ra$target_cluster ~ Case_status + collection_method + seqs_scaled,
            data=data.frame(sample_data(ra.ps.s)))

# coalesce results
FC <- paste(round(2^summary(ra.lm)$coefficients[2,1],2), ' [',
            round(2^confint(ra.lm)[2,1],2),'-',round(2^confint(ra.lm)[2,2],2),']',sep='')
mod.results <- data.frame(Grouping="Prevotella (SILVAv132)",
              `N PD`=sum(ra.mod$target_cluster[sample_data(ra.ps.s)$Case_status == 1] > 0),
              `N NHC`=sum(ra.mod$target_cluster[sample_data(ra.ps.s)$Case_status == 0] > 0),
              `RA in PD`=formatC(mean(ra.mod$target_cluster[sample_data(ra.ps.s)$Case_status == 1]),
                                 format='e',digits=1),
              `RA in NHC`=formatC(mean(ra.mod$target_cluster[sample_data(ra.ps.s)$Case_status == 0]),
                                  format='e',digits=1),
              Beta=round(summary(ra.lm)$coefficients[2,1],2),
              SE=round(summary(ra.lm)$coefficients[2,2],2),
              P=formatC(summary(ra.lm)$coefficients[2,4],format='e',digits=1),
              FC=FC,
              check.names=FALSE)

# write results
# create workbook
wb <- createWorkbook()
# add worksheet, write data, and format output
addWorksheet(wb, 'Prevotella (SILVAv132)')
```

```
writeData(wb, 'Prevotella (SILVAv132)', mod.results, keepNA=TRUE, colNames=TRUE)
setColWidths(wb, 'Prevotella (SILVAv132)', cols=seq_len(ncol(mod.results)),
             widths=c(18, rep(7,7), 13)) ### format cells
addStyle(wb, 'Prevotella (SILVAv132)', cols=seq_len(ncol(mod.results)),
         rows=1:(nrow(mod.results)+1), gridExpand=TRUE, style=center, stack=TRUE)
addStyle(wb, 'Prevotella (SILVAv132)', cols=seq_len(ncol(mod.results)),
         rows=1, style=bold, stack=TRUE) ### font
addStyle(wb, 'Prevotella (SILVAv132)', cols=seq_len(ncol(mod.results)),
         rows=c(1,2,(nrow(mod.results)+2)), ### borders
         gridExpand=TRUE, style=horizontal_border_med, stack=TRUE)
# convert numbers from strings back to numbers
convertNum(mod.results, wb, 'Prevotella (SILVAv132)', TRUE)
# save workbook
saveWorkbook(wb,
    'PDShotgunAnalysis_out/6.Secondary_analyses/Prevotella_SILVA_v_132.xlsx',
    overwrite=TRUE)
```

Replicating signal for opportunitstic pathogen cluster

After correlation networks were constructed, we noted a poly-microbial cluster of species (cluster 17 in the PD network) that resembled that of a cluster of opportunitstic pathogens noted in our previous 16S analysis (*Wallen et al. 2020 npj Parkinsons Dis*). As done in *Wallen et al. 2020*, to test if all of the species in this cluster are significantly enriched in PD (since only *P. asaccharolytica* was the only member of this cluster prevalent enough to be tested in MWAS), collpased relative abundances of cluster members (as shown in PD network cluster 17) and tested for association with PD using linear regression with log2 transformed relative abundances (as done in MaAsLin2) adjusting for total sequence count per sample and collection method. **Please note that the cluster numbers may be assigned differently by different operating systems, e.g 13 instead of 17

```
#### ASSOCIATION OF OPP. PATH. CLUSTER WITH PD ####

#### FULL CLUSTER ####

# collapse species relative abundances into one group
ra.mod <- data.frame(otu_table(ra.ps.s)/100)
colnames(ra.mod) <- sapply(strsplit(as.character(colnames(ra.mod)), "s__"),
                          function(x){x[2]})
ra.mod <- data.frame(target_cluster=rowSums(ra.mod[,colnames(ra.mod) %in%
                                          pd.clusters$names[pd.clusters$membership == 17]]),
                     ra.mod[,!(colnames(ra.mod) %in%
                               pd.clusters$names[pd.clusters$membership == 17])])

# log2 transform
log2.ra <- data.frame(apply(ra.mod, 2, log2.trans))

# perform linear regression
ra.lm <- lm(log2.ra$target_cluster ~ Case_status + collection_method + seqs_scaled,
            data=data.frame(sample_data(ra.ps.s)))

# coalesce results
FC <- paste(round(2^summary(ra.lm)$coefficients[2,1],2), ' [',
           round(2^confint(ra.lm)[2,1],2),'-',round(2^confint(ra.lm)[2,2],2),']',sep='')
mod.results <- data.frame(Grouping="All 19 species in cluster #17",
              `N PD`=sum(ra.mod$target_cluster[sample_data(ra.ps.s)$Case_status == 1] > 0),
```

```
              `N NHC`=sum(ra.mod$target_cluster[sample_data(ra.ps.s)$Case_status == 0] > 0),
              `RA in PD`=formatC(mean(ra.mod$target_cluster[sample_data(ra.ps.s)$Case_status == 1]),
                                 format='e',digits=1),
              `RA in NHC`=formatC(mean(ra.mod$target_cluster[sample_data(ra.ps.s)$Case_status == 0]),
                                  format='e',digits=1),
              Beta=round(summary(ra.lm)$coefficients[2,1],2),
              SE=round(summary(ra.lm)$coefficients[2,2],2),
              P=formatC(summary(ra.lm)$coefficients[2,4],format='e',digits=1),
              FC=FC,
              check.names=FALSE)

#### REDUCED CLUSTER (no PD-associated species included) ####

# collapse species relative abundances into one group
ra.mod <- data.frame(otu_table(ra.ps.s)/100)
colnames(ra.mod) <- sapply(strsplit(as.character(colnames(ra.mod)), "s__"), function(x){x[2]})
ra.mod <- data.frame(target_cluster=rowSums(ra.mod[,(colnames(ra.mod) %in%
                                            pd.clusters$names[pd.clusters$membership == 17]) &
                                            !(colnames(ra.mod) %in%
                                            nodes.s$Id[grep("Yes", nodes.s$`PD-associated`)])]),
                     ra.mod[,!(colnames(ra.mod) %in%
                               pd.clusters$names[pd.clusters$membership == 17]) |
                               colnames(ra.mod) %in%
                               nodes.s$Id[grep("Yes", nodes.s$`PD-associated`)]])

# log2 transform
log2.ra <- data.frame(apply(ra.mod, 2, log2.trans))

# perform linear regression
ra.lm <- lm(log2.ra$target_cluster ~ Case_status + collection_method + seqs_scaled,
             data=data.frame(sample_data(ra.ps.s)))

# coalesce results
FC <- paste(round(2^summary(ra.lm)$coefficients[2,1],2), ' [',
            round(2^confint(ra.lm)[2,1],2),'-',round(2^confint(ra.lm)[2,2],2),']',sep='')
mod.results <- rbind(mod.results,
            data.frame(Grouping="Cluster #17 excluding P. asaccharolytica",
              `N PD`=sum(ra.mod$target_cluster[sample_data(ra.ps.s)$Case_status == 1] > 0),
              `N NHC`=sum(ra.mod$target_cluster[sample_data(ra.ps.s)$Case_status == 0] > 0),
              `RA in PD`=formatC(mean(ra.mod$target_cluster[sample_data(ra.ps.s)$Case_status == 1]),
                                 format='e',digits=1),
              `RA in NHC`=formatC(mean(ra.mod$target_cluster[sample_data(ra.ps.s)$Case_status == 0]),
                                  format='e',digits=1),
              Beta=round(summary(ra.lm)$coefficients[2,1],2),
              SE=round(summary(ra.lm)$coefficients[2,2],2),
              P=formatC(summary(ra.lm)$coefficients[2,4],format='e',digits=1),
              FC=FC,
              check.names=FALSE))

#### P. ASACCHAROLYTICA ONLY ####

# perform linear regression
ra.lm <- lm(log2.ra$Porphyromonas_asaccharolytica ~ Case_status + collection_method + seqs_scaled,
            data=data.frame(sample_data(ra.ps.s)))
```

```r

# coalesce results
FC <- paste(round(2^summary(ra.lm)$coefficients[2,1],2), ' [',
            round(2^confint(ra.lm)[2,1],2),'-',round(2^confint(ra.lm)[2,2],2),']',sep='')
mod.results <- rbind(mod.results,
data.frame(Grouping="P. asaccharolytica only",
`N PD`=sum(ra.mod$Porphyromonas_asaccharolytica[sample_data(ra.ps.s)$Case_status == 1] > 0),
`N NHC`=sum(ra.mod$Porphyromonas_asaccharolytica[sample_data(ra.ps.s)$Case_status == 0] > 0),
`RA in PD`=formatC(mean(ra.mod$Porphyromonas_asaccharolytica[sample_data(ra.ps.s)$Case_status == 1]),
                   format='e',digits=1),
`RA in NHC`=formatC(mean(ra.mod$Porphyromonas_asaccharolytica[sample_data(ra.ps.s)$Case_status == 0]),
                    format='e',digits=1),
Beta=round(summary(ra.lm)$coefficients[2,1],2),
SE=round(summary(ra.lm)$coefficients[2,2],2),
P=formatC(summary(ra.lm)$coefficients[2,4],format='e',digits=1),
FC=FC, check.names=FALSE))

# write results
# create worbook
wb <- createWorkbook()
# add worksheet, write data, and format output
addWorksheet(wb, 'Cluster 17')
writeData(wb, 'Cluster 17', mod.results, keepNA=TRUE, colNames=TRUE)
setColWidths(wb, 'Cluster 17', cols=seq_len(ncol(mod.results)),
             widths=c(24, rep(7,7), 13)) ### format cells
addStyle(wb, 'Cluster 17', cols=seq_len(ncol(mod.results)),
         rows=1:(nrow(mod.results)+1), gridExpand=TRUE, style=center, stack=TRUE)
addStyle(wb, 'Cluster 17', cols=seq_len(ncol(mod.results)),
         rows=1, style=bold, stack=TRUE) ### font
addStyle(wb, 'Cluster 17', cols=seq_len(ncol(mod.results)),
         rows=c(1,2,(nrow(mod.results)+2)), ### borders
         gridExpand=TRUE, style=horizontal_border_med, stack=TRUE)
# convert numbers from strings back to numbers
convertNum(mod.results, wb, 'Cluster 17', TRUE)
# save workbook
saveWorkbook(wb,
  'PDShotgunAnalysis_out/6.Secondary_analyses/Cluster_17_PDvsNHC.xlsx',
  overwrite=TRUE)
```

R session information

```r
#### R SESSION INFO ####

sessionInfo()
```

```
## R version 4.0.3 (2020-10-10)
## Platform: x86_64-pc-linux-gnu (64-bit)
## Running under: Red Hat Enterprise Linux
##
## Matrix products: default
## BLAS/LAPACK: /data/rc/apps/rc/software/OpenBLAS/0.2.20-GCC-6.4.0-2.28/lib/libopenblas_haswellp-r0.2.2
##
## locale:
```

```
##  [1] LC_CTYPE=en_US.UTF-8       LC_NUMERIC=C
##  [3] LC_TIME=en_US.UTF-8        LC_COLLATE=en_US.UTF-8
##  [5] LC_MONETARY=en_US.UTF-8    LC_MESSAGES=en_US.UTF-8
##  [7] LC_PAPER=en_US.UTF-8       LC_NAME=C
##  [9] LC_ADDRESS=C               LC_TELEPHONE=C
## [11] LC_MEASUREMENT=en_US.UTF-8 LC_IDENTIFICATION=C
##
## attached base packages:
## [1] grid      stats     graphics  grDevices utils     datasets  methods
## [8] base
##
## other attached packages:
##  [1] igraph_1.2.6      Maaslin2_1.8.0    ANCOMBC_1.6.2     vcd_1.4-9
##  [5] pairwiseCI_0.1-27 coin_1.4-2        survival_3.2-13   MCPAN_1.1-21
##  [9] vegan_2.5-7       lattice_0.20-45   permute_0.9-7     ggrepel_0.9.1
## [13] ggvenn_0.1.9      ggfortify_0.4.14  ggh4x_0.2.1       ggplot2_3.3.6
## [17] scales_1.2.1      gridExtra_2.3     data.table_1.14.2 foreach_1.5.2
## [21] openxlsx_4.2.5    tibble_3.1.8      phyloseq_1.34.0   readxl_1.3.1
## [25] reshape2_1.4.4    dplyr_1.0.9
##
## loaded via a namespace (and not attached):
##   [1] backports_1.4.1     Hmisc_4.6-0         plyr_1.8.6
##   [4] splines_4.0.3       TH.data_1.1-0       lpsymphony_1.18.0
##   [7] digest_0.6.29       htmltools_0.5.2     fansi_1.0.3
##  [10] magrittr_2.0.3      checkmate_2.0.0     cluster_2.1.2
##  [13] doParallel_1.0.17   Biostrings_2.58.0   matrixStats_0.61.0
##  [16] MCMCpack_1.6-1      sandwich_3.0-1      jpeg_0.1-9
##  [19] colorspace_2.0-3    rbibutils_2.2.7     xfun_0.29
##  [22] crayon_1.5.0        jsonlite_1.8.0      libcoin_1.0-9
##  [25] biglm_0.9-2.1       Exact_3.1           zoo_1.8-9
##  [28] iterators_1.0.14    ape_5.6-1           glue_1.6.2
##  [31] gtable_0.3.0        zlibbioc_1.36.0     XVector_0.30.0
##  [34] MatrixModels_0.5-0  Rhdf5lib_1.12.1     DEoptimR_1.0-10
##  [37] BiocGenerics_0.36.1 abind_1.4-5         SparseM_1.81
##  [40] mvtnorm_1.1-3       DBI_1.1.2           rngtools_1.5.2
##  [43] Rcpp_1.0.8          htmlTable_2.4.0     magic_1.6-0
##  [46] foreign_0.8-82      proxy_0.4-26        Formula_1.2-4
##  [49] stats4_4.0.3        getopt_1.20.3       htmlwidgets_1.5.4
##  [52] RColorBrewer_1.1-3  modeltools_0.2-23   pkgconfig_2.0.3
##  [55] nnet_7.3-17         utf8_1.2.2          tidyselect_1.1.2
##  [58] rlang_1.0.4         munsell_0.5.0       cellranger_1.1.0
##  [61] tools_4.0.3         cli_3.3.0           generics_0.1.3
##  [64] ade4_1.7-18         evaluate_0.15       biomformat_1.18.0
##  [67] stringr_1.4.0       fastmap_1.1.0       yaml_2.3.5
##  [70] mcmc_0.9-7          knitr_1.37          robustbase_0.93-9
##  [73] zip_2.2.0           purrr_0.3.4         rootSolve_1.8.2.3
##  [76] nlme_3.1-155        doRNG_1.8.2         quantreg_5.88
##  [79] mcprofile_1.0-1     compiler_4.0.3      rstudioapi_0.13
##  [82] png_0.1-7           e1071_1.7-9         pcaPP_1.9-74
##  [85] DescTools_0.99.44   stringi_1.7.6       gsl_2.1-7.1
##  [88] Matrix_1.4-0        nloptr_1.2.2.2      microbiome_1.12.0
##  [91] multtest_2.46.0     vctrs_0.4.1         pillar_1.8.1
##  [94] lifecycle_1.0.1     rhdf5filters_1.2.1  optparse_1.7.1
##  [97] Rdpack_2.1.4        lmtest_0.9-39       lmom_2.8
```

```
## [100] R6_2.5.1           latticeExtra_0.6-29 IRanges_2.24.1
## [103] gld_2.6.4          codetools_0.2-18    boot_1.3-28
## [106] energy_1.7-10      MASS_7.3-55         rhdf5_2.34.0
## [109] withr_2.5.0        multcomp_1.4-18     S4Vectors_0.28.1
## [112] mgcv_1.8-39        expm_0.999-6        parallel_4.0.3
## [115] quadprog_1.5-8     rpart_4.1.16        tidyr_1.2.0
## [118] coda_0.19-4        class_7.3-20        rmarkdown_2.11
## [121] Rtsne_0.15         Biobase_2.50.0      base64enc_0.1-3
```
