# Supplementary material for: Metagenomics of Parkinson’s disease implicates the gut microbiome in multiple disease mechanisms
Source: Nat Commun. 2022 Nov 15;13:6958. doi: 10.1038/s41467-022-34667-x (PMC9663292; doi:10.1038/s41467-022-34667-x)
Supplement: Supplementary file 7 — Reporting Summary [file 41467_2022_34667_MOESM7_ESM.pdf]

nature portfolio

Corresponding author(s): Haydeh Payami

Last updated by author(s): Oct 26, 2022

Reporting Summary

Nature Portfolio wishes to improve the reproducibility of the work that we publish. This form provides structure for consistency and transparency in reporting. For further information on Nature Portfolio policies, see our Editorial Policies and the Editorial Policy Checklist.

Statistics

For all statistical analyses, confirm that the following items are present in the figure legend, table legend, main text, or Methods section.

n/a	Confirmed	
☐	☒	The exact sample size (*n*) for each experimental group/condition, given as a discrete number and unit of measurement
☐	☒	A statement on whether measurements were taken from distinct samples or whether the same sample was measured repeatedly
☐	☒	The statistical test(s) used AND whether they are one- or two-sided<br>*Only common tests should be described solely by name; describe more complex techniques in the Methods section.*
☐	☒	A description of all covariates tested
☐	☒	A description of any assumptions or corrections, such as tests of normality and adjustment for multiple comparisons
☐	☒	A full description of the statistical parameters including central tendency (e.g. means) or other basic estimates (e.g. regression coefficient) AND variation (e.g. standard deviation) or associated estimates of uncertainty (e.g. confidence intervals)
☐	☒	For null hypothesis testing, the test statistic (e.g. *F*, *t*, *r*) with confidence intervals, effect sizes, degrees of freedom and *P* value noted<br>*Give P values as exact values whenever suitable.*
☒	☐	For Bayesian analysis, information on the choice of priors and Markov chain Monte Carlo settings
☒	☐	For hierarchical and complex designs, identification of the appropriate level for tests and full reporting of outcomes
☐	☒	Estimates of effect sizes (e.g. Cohen's *d*, Pearson's *r*), indicating how they were calculated

Our web collection on statistics for biologists contains articles on many of the points above.

Software and code

Policy information about availability of computer code

Data collection	no software used for data collection
Data analysis	ANCOMBC v 1.6.2; BBDuk and BBSplit v 38.92; ChocoPhlAn v 201901b; EMBL enterotype classifier v 2016; FastSpar v 1.0.0; Gephi v 0.9.2; ggfortify v 0.4.14; ggplot2 v 3.3.6; ggvenn v 0.1.9; Human reference genome v GRCh38.p13; HUMAnN v 3.0.0; HUMAnN utility tool humann_regroup_table v 3.0.0; igraph v 1.2.6; Maaslin2 v 1.8.0; MetaCyc v 24; MetaPhlAn v 3.0.14; pairwiseCI v 0.1.27; PROGENY v 9; R v 4.0.3; SILVA v 132; UniRef90 v 201901b; vcd v 1.4.9; vegan v 2.5.7; Microsoft Excel v 16.42

For manuscripts utilizing custom algorithms or software that are central to the research but not yet described in published literature, software must be made available to editors and reviewers. We strongly encourage code deposition in a community repository (e.g. GitHub). See the Nature Portfolio guidelines for submitting code & software for further information.

Data

Policy information about availability of data

All manuscripts must include a data availability statement. This statement should provide the following information, where applicable:

- Accession codes, unique identifiers, or web links for publicly available datasets
- A description of any restrictions on data availability
- For clinical datasets or third party data, please ensure that the statement adheres to our policy

The datasets generated and analyzed during the current study are available to the public with no restrictions at the NCBI Sequence Read Archive (SRA) under BioProject ID PRJNA834801 [https://www.ncbi.nlm.nih.gov/bioproject/834801]. Sequences on SRA are pre-QC with the exception that SRA removed human

sequences prior to making them public. “Source Data”, the post-QC and post taxonomic and functional profiling data generated here, is provided to the public with no restrictions at Zenodo [https://zenodo.org/record/7246185]. The human reference genome used for decontaminating metagenomic sequences (GRCh38.p13) is publicly available at NCBI under the GenBank assembly accession number GCA_000001405.28 [https://www.ncbi.nlm.nih.gov/assembly/GCA_000001405.28]. The ChocoPhlAn database (v30) used by MetaPhlAn3 for taxonomic profiling is publicly available for download from the MetaPhlAn databases FTP site [http://cmprod1.cibio.unitn.it/biobakery3/metaphlan_databases] or by running the command `metaphlan --install`. The ChocoPhlAn and UniRef90 reference databases (v201901b) used during functional profiling with HUMAnN3 are publicly available to download from the HUMAnN data FTP site [http://huttenhower.sph.harvard.edu/humann_data] or by using the `humann_databases` utility script. Pathway data for functional profiling of pathways by HUMAnN come packaged with the HUMAnN program and were derived from the publicly accessible MetaCyc reference database (v24) [https://metacyc.org/].huttenhower.sph.harvard.edu/humann_data] or by using the `humann_databases` utility script. Pathway data for functional profiling of pathways by HUMAnN come packaged with the HUMAnN program and were derived from the publicly accessible MetaCyc reference database (v24) [https://metacyc.org/].

Human research participants

Policy information about studies involving human research participants and Sex and Gender in Research.

Reporting on sex and gender

We followed SAGER Guidelines.
Self-reported biological sex was used.
We state in manuscript: biological sex (not gender).
Findings apply to both sexes.
Sex was considered in study design.
Sex was determined by self-report.
"Source data" file and raw data on NCBI SRA include disaggregated sex data on all subjects at individual level.
Consent has been obtained for sharing de-identified individual-level data.
Overall numbers are 380 males and 344 females.
Sex-based analyses have been reported in Methods and Results.

Population characteristics

Covariate-relevant characteristics (age, sex, GI health, medications, diet, weight loss) are provided in Table 1, and discussed in Results.
Past and current diagnosis of PD are provided in Figure 1 and discussed in Methods and Results. All cases had a diagnosis of PD at enrollment, those whose dx changed between enrollment and analysis were identified and excluded.
Past and current diagnosis of neurological disorders, exclusion criteria for controls, are provided in Methods and Figure 1.

Recruitment

670 individuals with PD and 316 NHC were enrolled at University of Alabama at Birmingham (UAB) between October 2018 and March 2020. All subjects were from the same geographic region, including the city of Birmingham and surrounding areas in the southern US, keeping confounding effect of geography on the microbiome to a minimum. The eligibility criteria to enroll as a case was diagnosis of PD and informed consent. Potential PD cases for enrollment were identified via systematic pre-screening of electronic medical records (EMR) of patients with an upcoming appointment in the Movement Disorder Clinic at UAB. Subjects were invited to enroll in the study after their clinic visit if the attending specialist confirmed PD diagnosis and the patient was willing to hear about the study. With each potential participant, in a private setting, our recruitment coordinator explained the study and their involvement, subject was given time to read the Informed Consent Form, to ask questions, and if they agreed to participate, sign the consent. During enrollment visit, subjects donated a blood or saliva sample, and took home two questionnaires and a stool collection kit to complete and return in the pre-stamped envelope via US Postal Service. The spouse or friend accompanying the patient was invited to enroll as control, to match for shared environmental effects. Additional control subjects were recruited from the community. Consent, enrolment, and data collection process for controls was the same as for patients if the control was met in person. For community volunteers, we first had a phone conversation to explain the study, then we mailed the Informed Consent Form to them with a phone number to call with any questions, those who returned a signed Informed Consent Form were sent a kit for saliva collection, a kit for stool collection, and two questionnaires to complete at home and return by mail. The eligibility criteria to enroll as a control was absence of PD, REM sleep behavior disorder, Alzheimer’s disease, dementia, multiple sclerosis, amyotrophic lateral sclerosis, ataxia, dystonia, autism, epilepsy, stroke, bipolar disorder, and schizophrenia by self-report (hence, neurologically healthy controls), preferably being over age 50 years, and informed consent. We tried not to enroll subjects who had participated in our prior microbiome studies to maintain cohort independence; however, 11 subjects were inadvertently double-enrolled. We ended the enrollment at the start of the COVID-19 pandemic in the southern US (March 2020) to avoid confounding by infection or stress of the pandemic.

Potential sources of bias and how they were dealt with:
(1) Preponderance of males among PD (disease feature) vs. preponderance of females in controls: data were re-analyzed adjusting for sex statistically; results were robust.
(2) Slightly younger ages of controls: data were re-analyzed while adjusting for age statistically; results were robust.
(3) We tested the distribution of 53 subject metadata elements (54 including case status) in cases vs. controls, and those that differed and were not intrinsic features of PD were identified as potential confounders: data were re-analyzed while adjusting for the potential confounders, results are reported in Table 2.
(4) Self-selection: Nearly 25% of subjects who had consented and committed to provide a stool sample never did. The distribution was the same in cases and controls, thus it would not have affected the results.

Ethics oversight

Study was approved by the Institutional Review Board (IRB) for Protection of Human subjects at the University of Alabama at Birmingham (UAB) and by the Human Research Protection Office (HRPO) of United States Department of Defense (funding agency).

Note that full information on the approval of the study protocol must also be provided in the manuscript.

Field-specific reporting

Please select the one below that is the best fit for your research. If you are not sure, read the appropriate sections before making your selection.

- [x] Life sciences
- [ ] Behavioural & social sciences
- [ ] Ecological, evolutionary & environmental sciences

For a reference copy of the document with all sections, see nature.com/documents/nr-reporting-summary-flat.pdf

Life sciences study design

All studies must disclose on these points even when the disclosure is negative.

Sample size

Sample size was pre-determined in a funded grant based on power calculation and feasibility. We aimed for maximum N that could be enrolled, with intensive effort, in a two-year period (2018-2020), stopping at the arrival of the COVID-19 pandemic. We achieved the largest N of any disease-microbiome study to date. Per power calculation, the sample size was sufficient to detect the magnitudes of effect sizes that we and others have seen for association of taxa with disease. Sample size was not sufficient for extremely rare taxa: for statistical purposes, we pre-set a minimum N=37 carriers (stringent compared to most studies) for a given taxa; taxa that had lower prevalence than 5% (N<37) were excluded from MWAS. Again, to my knowledge, no single microbiome disease study to date has had more power than this study.

Data exclusions

Exclusions have been outlined in Figure 1 and discussed in Methods. Exclusion criteria were pre-established. Exclusions were applied at four levels: subjects, metadata, metagenome sequences, taxa.

Subject exclusions: did not return stool sample, PD diagnosis changed, neurological disease in control, potential sample mix-up, stool collected during COVID-19 pandemic, low sequence count.

Metadata exclusion: data points that were not answered or were unclear were excluded while retaining the subject and the reliable data and sample.

Sequence exclusions: removed low quality sequences, human sequences, low complexity sequences (i.e., monomeric repeats), sequences <70bp (MetaPhlAn), too low alignment to marker gene (MetaPhlAn) and/or protein (HUMAnN) databases.

Taxa exclusions: taxa that were present in <5% of subjects (N<37) were excluded from statistical analysis.

Replication

All attempts at replication were successful.

Replication was implemented at two levels:
(1) Methodology. We conducted unbiased metagenome-wide association studies (MWAS) using two statistical methods (MaAsLin2 and ANCOM-BC) and focused on concordant findings to guard against methodological variation. We nominated a species or genus as PD-associated if it achieved significance by both MaAsLin2 and ANCOM-BC (i.e., false discovery rate (FDR)<0.05 by one and FDR<=0.1 by the other).
(2) Independent datasets. We have previously conducted and published MWAS on two independent cohorts of PD and NHC subjects using 16S rRNA amplicon sequencing (N1=333 and N2=507). Present dataset was also an MWAS on a third independent dataset of PD and NHC (N=724) but generated with deep shotgun sequencing. We successfully replicated every finding of 16S studies using shotgun on the third dataset and resolved them to species-level. Replication that were attempted included (a) results of MWAS (microorganisms associated with PD) and (b) results of network analysis (polymicrobial clusters formed by PD-associated microorganisms), and all were replicated successfully.

Other measures taken for reproducibility: QC was implemented at every step possible during enrollment and data collection (described in Methods). Metadata were double entered by two staff and errors identified and corrected. Bioinformatics and statistical analyses were conducted independently by a student and a post-doc and double-checked.

Randomization

Case and control samples were inter-mixed for plating to avoid batch effect during DNA isolation and sequencing.

Blinding

Metadata were collected using self-administered questionnaires to avoid investigator bias.

DNA extraction and sequencing was conducted at a metagenomics company that was blind to status of the samples.

Bioinformatics were conducted using pre-established QC and taxonomic assignment protocols that are independent of case status.

Statistical analyses were conducted using unbiased (investigator-blind) metagenome-wide association study and network analysis.

Reporting for specific materials, systems and methods

We require information from authors about some types of materials, experimental systems and methods used in many studies. Here, indicate whether each material, system or method listed is relevant to your study. If you are not sure if a list item applies to your research, read the appropriate section before selecting a response.

Materials & experimental systems

n/a	Involved in the study
☒	☐ Antibodies
☒	☐ Eukaryotic cell lines
☒	☐ Palaeontology and archaeology
☒	☐ Animals and other organisms
☒	☐ Clinical data
☒	☐ Dual use research of concern

Methods

n/a	Involved in the study
☒	☐ ChIP-seq
☒	☐ Flow cytometry
☒	☐ MRI-based neuroimaging
